# A complete series of 6-deoxy-monosubstituted tetraalkylammonium derivatives of α-, β-, and γ-cyclodextrin with 1, 2, and 3 permanent positive charges

**DOI:** 10.3762/bjoc.10.142

**Published:** 2014-06-18

**Authors:** Martin Popr, Simona Hybelbauerová, Jindřich Jindřich

**Affiliations:** 1 Department of Organic Chemistry, Faculty of Science, Charles University in Prague, Hlavova 8, 128 40, Prague 2, Czech Republic, Fax: +420 22195 1326; 2 Department of Teaching and Didactics of Chemistry, Faculty of Science, Charles University in Prague, Hlavova 8, 128 40, Prague 2, Czech Republic

**Keywords:** cationic, cyclodextrins, monosubstitution, regioselectivity, tetraalkylammonium derivatives

## Abstract

An efficient synthetic route toward the preparation of a complete series of monosubstituted tetraalkylammonium cyclodextrin (CD) derivatives is presented. Monotosylation of native CDs (α-, β-, γ-) at position 6 gave the starting material. Reaction of monotosylate (mono-Ts-CD) with 45% aqueous trimethylamine gave CDs substituted with one cationic functional group in a single step. Derivatives equipped with a substituent containing two cationic sites separated by an ethylene or a propylene linker were prepared by reacting mono-Ts-CD with neat *N*,*N*,*N*’-trimethylethane-1,2-diamine or *N*,*N*,*N*’-trimethylpropane-1,3-diamine and subsequent methylation by CH_3_I in good yields. Finally, analogues bearing a moiety with three tetraalkylammonium sites were synthesized by reacting mono-Ts-CD with bis(3-aminopropyl)amine and subsequent methylation. The majority of the presented reactions are very straightforward with a simple work-up, which avoids the need of chromatographic separation. Thus, these reactions are suitable for the multigram-scale production of monosubstituted cationic CDs.

## Introduction

Cyclodextrins (CDs) are cyclic oligosaccharides with the shape of a hollow truncated cone, first described in 1891 by Villiers [1]. Naturally occurring CDs are named α-, β- and γ-cyclodextrin and are composed of 6, 7 or 8 D-glucopyranose units, respectively. The most commonly utilized feature of native CDs is their ability to form noncovalent inclusion complexes with a wide range of guest molecules [2]. In the majority of industrial applications, natural CDs serve as encapsulation agents with a high affinity toward suitable lipophilic organic guests [3]. The main areas of use of native CDs and their derivatives are drug transportation [4] and solubilization [5], catalysis [6] and enzyme mimics [7].

Chemically modified CDs [5,8] are prepared in order to reach specific chemical and physical properties, e.g., solubility or their binding behavior [9]. Many different CD derivatives have been described in the literature, but most of the preparations suffer from the polydispersity of the products, low yields and poor selectivity due to the large number of hydroxy groups. Among the useful derivatizations are, for example, permethylation [10], acetylation [11] or hydroxypropylation [12] and perfacial substitution of all primary 6-OH hydroxy groups by iodine [13]. The products of these reactions are easily accessible in high amounts and are used in the pharmaceutical industry [14] or as precursors for further derivatizations.

Monosubstituted CDs, i.e., CDs with only one hydroxy group substituted, are the most difficult to prepare, but can find use in many applications. The selectivity of the substitution can be achieved by taking advantage of the different reactivity of primary and secondary hydroxy groups. Reactions employing one equivalent of derivatizing agent along with an excess of base lead to the 6^I^-*O*-substituted products, while the preparation of 2^I^-*O*-substituted derivatives [15] takes advantage of the highest acidity of 2-OH hydroxy groups. A widely used methodology that leads to a variety of 6^I^-*O*-substituted analogues is the reaction with *p*-toluenesulfonic chloride [16] or *p*-toluenesulfonic anhydride [17] and an excess of base, which affords 6^I^-*O*-tosyl-CD. This can be converted into the desired product by a reaction with an appropriate nucleophile.

Single-isomer permanently charged cationic CD derivatives proved to serve as excellent chiral selectors in capillary zone electrophoresis (CZE) [18–19] and as catalysts of chemical reactions [20–21]. The synthesis of some cationic derivatives of β-CD have already been described in the literature (namely 6^I^-(*N*,*N*,*N*-trimethylammonio)-6^I^-deoxy-β-cyclodextrin [21] and 6^I^-(*N*,*N*,*N*’,*N*‘,*N*‘-pentamethylethane-1,2-diammonio)-6^I^-deoxy-β-cyclodextrin) [22]. However, the literature lacks a detailed synthetic protocol, the optimization, or the full characterization of the products. Furthermore, α- and γ-CDs monofunctionalized with a tetraalkylammonium group have never been reported before. Therefore, we decided to develop robust reaction sequences for the preparation of complete sets (α-, β-, γ-) of single-isomer monosubstituted quaternary ammonium CD derivatives with 1, 2 or 3 permanent positive charges, suitable for high-scale production.

## Results and Discussion

**Figure 1:**
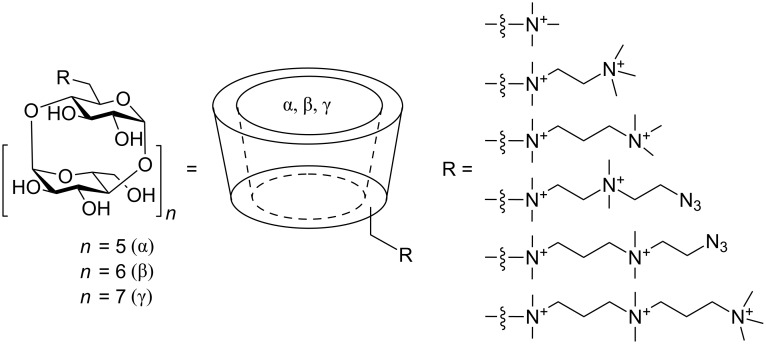
Schematic representation of the prepared sets of permanently charged CD derivatives.

This article presents the synthesis of a complete series of monosubstituted CD derivatives with a permanent positive charge (Figure 1).

### Synthesis of monotosylated CDs as the starting material

The most crucial and scope-limiting step of the reaction sequence was the introduction of a suitable leaving group in the position 6 of the native CD. For this purpose, the selective monotosylation of the free CD was chosen. Several methods for 6-O-tosylation have already been published [23–26]. Unfortunately, most of them lead to mixtures of mono- and multi-tosylated products, which cannot be separated by precipitation from acetone or crystalization from water. So we developed a new chromatography-free method for the purification of the reaction mixture by crystalization. Monosubstitution of β-CD was carried out by the reaction of *p*-toluenesulfonic anhydride in aqueous NaOH as described previously in literature [17]. The reaction yielded a mixture of monosubstituted product 6^I^-*O*-*p*-toluenesulfonyl-β-cyclodextrin (**1**), unreacted β-CD, and some highly-substituted derivatives, which needed to be separated in order to receive pure tosylate **1**. This fact is omitted in the original paper [17]. We used a strategy based on the repeated crystallization in a MeOH/H_2_O (1:1 v/v) mixture, which affords pure **1** after the third run in the satisfying overall yield of 26%. TLC and detection by carbonization proved to be a very simple yet very sensitive method to follow the purity of the tosylates.

For the monotosylation of α- and γ-CDs the reaction in water offers no advantage due to the high solubility of the products in aqueous solution (to precipitate out during the work-up) and the lower regioselectivity [27]. A conventional reaction with TsCl in pyridine [16] was used instead. Similarly as in the case of β-CD mentioned above, this reaction provided a mixture of products with several degrees of substitution. The recently published purification procedure [16] yielded just this mixture. The purification on a column of activated charcoal was also described [28]. We found the most convenient purification method to obtain 6^I^-*O*-*p*-toluenesulfonyl-α-cyclodextrin (**2**) and 6^I^-*O*-*p*-toluenesulfonyl-γ-cyclodextrin (**3**) is flash column chromatography on a reverse phase C18 column with a step gradient. The use of this method allowed for the recovery of about 40% of the starting α- or γ-CD by flushing the column with 10% MeOH. Pure monotosylated product is then eluted by 25% MeOH. This strategy affords compounds **2** and **3** in a very high purity and in sufficient yields of 32% and 50%, respectively (after subtraction of the recovered native CD).

### Synthesis of monotrimethylammonio CD derivatives

**Scheme 1:**
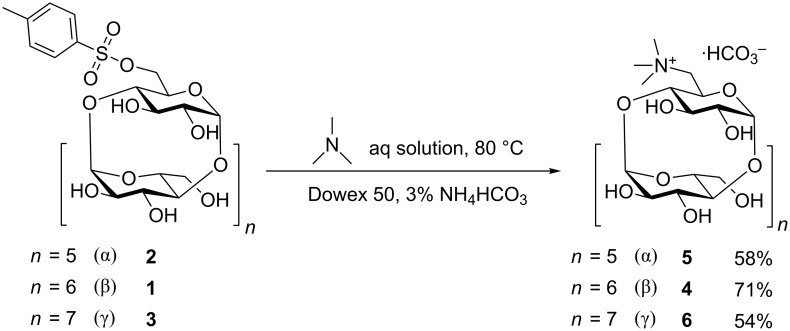
Synthesis of monotrimethylammonio-CD derivatives.

A procedure for the preparation of 6^I^-(*N*,*N*,*N*-trimethylammonio)-6^I^-deoxy-β-cyclodextrin bicarbonate (**4**) has been described in 1978 by Matsui et al. [21] and comprised the reaction of mono-Ts-β-CD with trimethylamine solution in DMF. Their procedure afforded the product **4** in only 42% yield, which may have been caused by the high amount of byproducts possibly formed by the decomposition of DMF or by the formylation side reactions. The authors recovered pure **4** after lengthy chromatographic separation on a carboxymethylcellulose column, involving the collection of 150 fractions. We decided to optimize the reaction conditions with the goal of improving the yield and avoiding the chromatography altogether. 45% v/v aqueous solution of trimethylamine, which is cheap and commercially available, was used (Scheme 1). Only two compounds were found in the reaction mixture – product **4** and byproduct 3,6-anhydro-β-cyclodextrin. The product **4** was easily separated on a short column of strong cation-exchange resin in a H^+^ cycle by flushing the column firstly with H_2_O to elute 3,6-anhydro-β-cyclodextrin and then with aqueous NH_4_HCO_3_ to elute the product. The yield of the reaction was 71%. Derivatives of α- and γ-cyclodextrin (**5** and **6**) with one tetralkylammonium group were prepared by the identical procedure in 58% and 54% yields, respectively (Scheme 1).

### Synthesis of CD derivatives monosubstituted with quaternary diamine

**Scheme 2:**
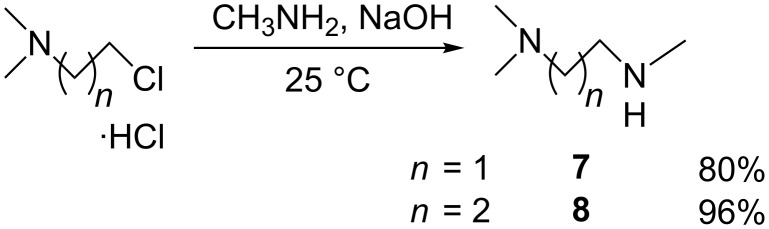
Preparation of diamines **7** and **8** as reagents for further synthesis [29].

**Scheme 3:**
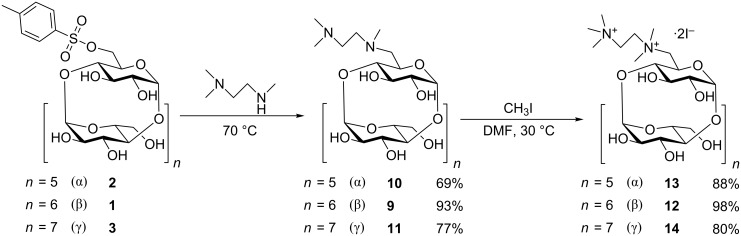
Synthesis of PEMEDA-CD derivatives.

6^I^-(*N*,*N*,*N*’,*N*‘,*N*‘-Pentamethylethane-1,2-diammonio)-6^I^-deoxy-β-cyclodextrin diiodide (PEMEDA-β-CD, **12**) was prepared by modification of the procedure described by Nzeadibe et al. [22], which neither provides the full NMR assignment of the intermediates and the product nor the yields of the reactions. In the first step, reagent *N*,*N*,*N*‘-trimethylethane-1,2-diamine (**7**) was easily prepared in one step by the published synthetic protocol [29], from inexpensive *N*-(2-chloroethyl)dimethylamine hydrochloride and MeNH_2_ (40% solution in H_2_O) (Scheme 2). In the next step, mono-Ts-β-CD reacts with **7** to obtain intermediate 6^I^-((2-(dimethylamino)-1-(methyl)ethyl)amino)-6^I^-deoxy-β-cyclodextrin (**9**) (Scheme 3). The advantage of this conversion is the absence of a solvent. Also, the unreacted **7** can be recycled from the reaction mixture by vacuum distillation. Pure intermediate **9** was then obtained in 93% yield by simple precipitation of the reaction mixture from propan-1-ol. In the final step, **9** was quaternized by methyl iodide in DMF. The reaction produced pure PEMEDA-β-CD after precipitation of the compound from acetone, without the need of any purification, in an excellent yield of 98%. PEMEDA derivatives of α- and γ-CDs were prepared by the procedure analogous to the preparation of PEMEDA-β-CD (Scheme 3). The only deviation was the purification of intermediates **10** and **11**, which cannot be precipitated from propan-1-ol. Instead, we employed precipitation from acetone with the subsequent separation of the byproduct (*p*-toluenesulfonic acid) on a short column of strong cation exchanger. This may be the reason of the lower yields of compounds **10** and **11**.

**Scheme 4:**
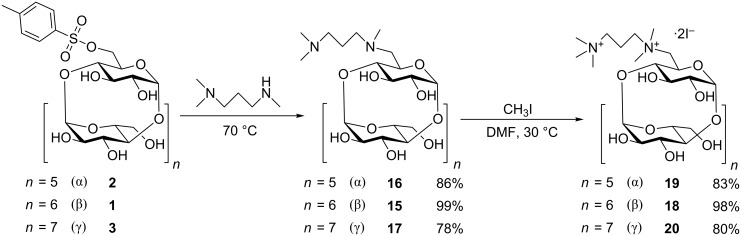
Synthesis of PEMPDA-CD derivatives.

We discovered that compound **12** and its analogues **13** and **14** are liable to decomposition by a Hofmann elimination in aqueous media, when subjected to higher temperatures or bases. The degradation can be monitored by TLC, where the decomposition product (olefin) was clearly visible. We decided to prepare another novel series of derivatives, where the two tetraalkylammonium groups are separated by a propylene linker instead of the ethylene one (Scheme 4). The same synthetic protocol as for the preparation of **12** and its α- and γ-analogues was employed, but with *N,N,N‘*-trimethylpropane-1,3-diamine (**8**). Products **18**, **19** and **20** with a linker between the charged nitrogen atoms (PEMPDA series) which is one methylene unit longer proved to be more stable and resistant toward the Hofmann elimination. Some preliminary measurements were performed and revealed a decomposition half-time about 2× longer for the series with a propylene linker. Again, yields of the products are very satisfactory, especially for β-CD derivatives.

The decomposition kinetics measurement was carried out in an NMR tube at 80 °C with 1 equivalent of NaOH as a base and a reaction time of 20 h. A ^1^H NMR spectrum was acquired every hour. Integrals of the signals of the H1 protons of CD were compared to integrals of the signals of the *N*-CH_3_ protons, which were disappearing in time due to the Hofmann elimination. Decomposition half-times were estimated to be 3 and 7 h for compounds **12** and **18**, respectively.

### Synthesis of CD derivatives monosubstituted with a quaternary diamine bearing an azidoethane function

**Scheme 5:**
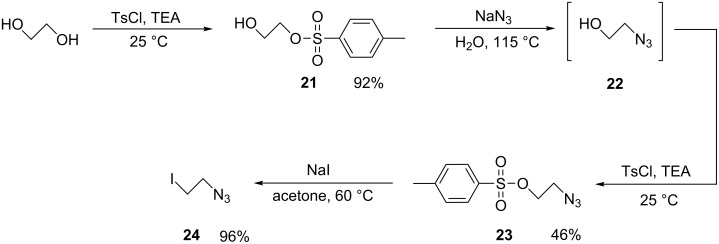
Synthesis of 1-azido-2-iodoethane.

Analogues of PEMEDA and PEMPDA-β-CD with an azidoethane function attached to the terminal quaternary ammonium nitrogen were prepared as a basis for further derivatization. The azide functional group is reactive and can be used, for example, for the attachment of various substituted acetylenes by a Huisgen 1,3-dipolar cycloaddition [30] (most popular ‘click reaction’) or the reduction to the amine. Our strategy involved the synthesis of the alkylation agent 1-azido-2-iodoethane (**24**) (Scheme 5) which would be used for the quaternization of the tertiary amine intermediate. The reaction sequence for the preparation of **24** involved a 4-step protocol. In the first step, one hydroxy group of ethylene glycol was substituted by tosylate to yield 92% of 2-hydroxyethyl-4-methylbenzenesulfonate (**21**). Next, compound **22** was prepared by a nucleophilic substitution with sodium azide. The product of this reaction was not isolated due to its possible explosive character, but was directly subjected to the tosylation to yield **23** [31]. Finally, compound **24** was prepared by means of the Finkelstein reaction (which was already published [32]) in 96% yield.

**Scheme 6:**
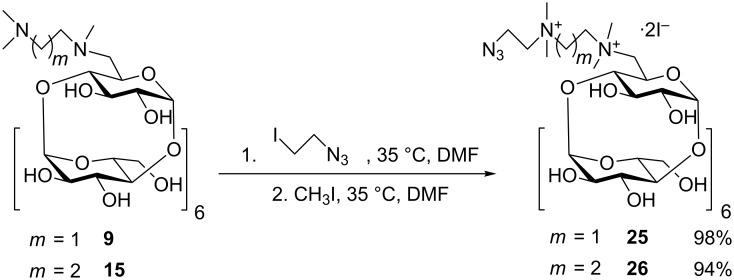
Synthesis of azidoethane-containing derivatives of PEMEDA and PEMPDA-β-CD.

We presumed that the alkylation of intermediate **9** by **24** in dry DMF would result in the product substituted on both nitrogens, but unfortunately the MS spectra revealed that only one nitrogen atom gets substituted. We tried different conditions (up to 50 equivalents of **24**, different sterically hindered bases, higher temperature) to achieve a conversion, but always only one azidoethane group was bound, possibly for steric reasons. So we decided to use a two-step procedure, which involved the attachment of **24** in the first step and the quaternization by MeI in the second step, without the need of the isolation of the intermediate (Scheme 6). This strategy afforded compounds **25** and **26** in 98% and 94% yields, respectively.

### Synthesis of CD derivatives monosubstituted with a quaternary triamine

**Scheme 7:**
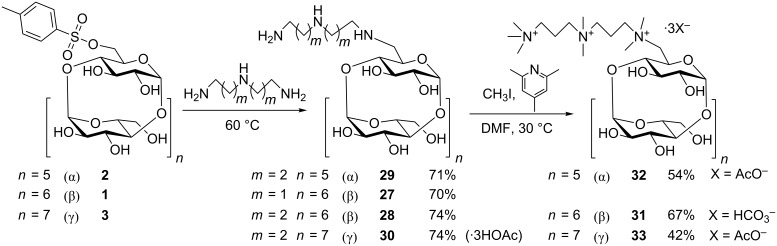
Synthesis of CD derivatives monosubstituted with a quaternary triamine moiety.

To complete the series of cationic CD derivatives, a set of CDs monosubstituted with permethylated linear triamine was prepared (Scheme 7). The dry mono-Ts-β-CD reacted in the first step with diethylenetriamine or bis(3-aminopropyl)amine in order to produce intermediates **27** and **28**, respectively. α- and γ- analogues **29** and **30** were prepared by an identical procedure in yields around 70%. Different routes were explored, and the best results were achieved by a modified procedure described by Tabushi et al. [33] (for β-CD only), which is a solvent-free reaction of the monotosylate with an excess of liquid triamine. The unreacted liquid triamine can be recovered from the reaction mixture by vacuum distillation and used repeatedly. Pure intermediates **27** and **28** were obtained after the elution with aqueous NH_4_OH, from a short column of strong cation exchanger in hydrogen form, in 70% and 74% yields, respectively. The derivative of γ-CD, compound **30**, contained some trace amounts of impurities and needed to be further purified on a silica gel column. In the following step, the installed triamine was quaternized by methylation with MeI. To achieve a full conversion, the use of a sterically hindered base was necessary. First, we attempted to methylate intermediate **27** containing a diethylenetriamine moiety. Different bases (K_2_CO_3_, 2,6-lutidine, 2,4,6-collidine and DBU) and temperatures (from rt to 50 °C) were tested, but we were not able to achieve a full conversion and the fraction of the product **27** in the reaction mixture was not stable either. Similarly, as in the case of PEMEDA derivatives, **27** is vulnerable toward decomposition by a Hofmann elimination. For this reason derivative **28**, which has bound bis(3-aminopropyl)amine in position 6 of β-CD, was prepared. In this derivative charged nitrogen atoms are separated by a longer propylene linker. Presumable, this was the reason that the methylation of this intermediate was more efficient, reached a full conversion after 20 h, and provided pure **31** after simple purification on cation-exchange column. Compounds **30**, **32** and **33** were obtained as triacetates after the purification on a silica gel column with a HOAc containing eluent.

## Conclusion

In summary, a complete series of permanently positively charged monosubstituted CD (α-, β-, γ-) derivatives were prepared. The synthesis was realized by the introduction of a substituent with one, two or three tetraalkylammonium groups on position 6 of native CD. Some reactions on β-CD were carried out by modification of already published procedures, but the majority of the products have not been described in the literature yet. Most of the products were obtained by simple conversions, in high yields and without the need of chromatographic purification. The complete characterization of the products is given, along with the unambiguous assignment of the positions of substituents by 2D NMR techniques. We believe that the products presented in this article will find their use as chiral selectors in CZE. They offer the possibility to choose the number of charges to tune the electrophoretic mobility. Furthermore, the diameter of the CD cavity can be modified so that the strength of the interaction with the isomers to be separated is adjustable to the task at hand. The study of the use of the prepared derivatives for chiral separations in CZE is currently ongoing.

## Supporting Information

File 1 File Name: S1.pdf.
Experimental part and copies of ^1^H and ^13^C NMR spectra of prepared compounds.
