# Supplementary material for: A complete series of 6-deoxy-monosubstituted tetraalkylammonium derivatives of α-, β-, and γ-cyclodextrin with 1, 2, and 3 permanent positive charges
Source: Beilstein J Org Chem. 2014 Jun 18;10:1390–6. doi: 10.3762/bjoc.10.142 (PMC4077373; doi:10.3762/bjoc.10.142)

Supporting Information

for

A complete series of 6-deoxy-monosubstituted tetraalkylammonium derivatives of α-, β-, and γ-cyclodextrin with 1, 2, and 3 permanent positive charges

Martin Popr[1], Simona Hybelbauerová[2] and Jindřich Jindřich[*1]

Address: [1]Department of Organic Chemistry, Faculty of Science, Charles University in Prague, Hlavova 8, 128 40, Prague 2, Czech Republic, Fax: +420 22195 1326 and [2]Department of Teaching and Didactics of Chemistry, Faculty of Science, Charles University in Prague, Hlavova 8, 128 40, Prague 2, Czech Republic

Email: Jindřich Jindřich[*] - jindrich@natur.cuni.cz

[*]Corresponding author

Experimental part and copies of ^{1}H and ^{13}C NMR spectra of prepared compounds

Content

General procedures and materials S2

Experimental part S3

References S30

^{1}H and ^{13}C NMR spectra of prepared compounds S31

Characterization of compounds by 2D NMR S85

2D NMR Spectra S87

General procedures and materials

^{1}H NMR spectra were acquired on Varian VNMRS 300 at 300 MHz and ^{13}C NMR at 75 MHz. For determination of the substituent position DEPT and 2D NMR measurements (H,H-COSY, HSQC and HMBC) were performed on Bruker AVANCE III at 600 MHz (^{1}H) and 125 MHz (^{13}C). Samples were dissolved in D_2O with a drop of *tert*-butanol or DMSO-d_6 with few drops of CD_3COOD. All chemical shift values (δ) are reported in ppm and coupling constants in Hz. Signals of tetramethylsilane (for ^{1}H NMR) and $CDCl_3$ (for ^{13}C NMR, $\delta = 77.0$ ppm) served as internal standards. Mass spectra were obtained on Bruker ESQUIRE 3000 ES-ion trap instrument with electrospray ionization (ESI) in positive mode. All samples were dissolved in methanol.

Infrared spectroscopy was performed on Thermo Nicolet AVATAR 370 FT-IR instrument. Samples were prepared as a suspension with KBr and measured via DRIFT method. HRMS–ESI spectra were acquired on Thermo Fisher Scientific LTQ Orbitrap XL instrument. Specific optical rotation was measured on Rudolph Research AUTOPOL III polarimeter. Melting points were estimated on Kofler apparatus NAGEMA-RAPIDO with temperature gradient 4 °C/min. Thin-layer chromatography (TLC) was performed on silica gel coated aluminium sheets DC-Alufolien Keisegel 60 F_{265} (Merck, Darmstadt, Germany). Dipping in 50% H_2SO_4 with subsequent carbonization by a heat gun was used for spot detection for all CD derivatives and by dipping in basic $KMnO_4$ for all non-CD derivatives. Fluka silica gel 60 (40–63 am; Fluka, Neu-Ulm, Germany) was used to perform preparative flash-column chromatography. Anhydrous DMF was prepared by distillation with P_2O_5 at reduced pressure and was stored over molecular sieves 3 Å under argon atmosphere. Anhydrous pyridine was prepared by standing over KOH for several days and subsequent distillation from CaH_2 under an atmosphere of Ar. Molecular sieves were activated in microwave oven Daewoo DMR-603 prior to use. Organic solvents were distilled prior to use. Other reagents were purchased from common commercial sources and used without further purification unless otherwise noted. β- and γ-CD were purchased from WAKO Chemicals (Germany). α-CD was purchased from Wacker Chemie.

Experimental part

6^{I}-*O*-*p*-Toluenesulfonyl-β-cyclodextrin (1). Compound **1** was prepared according to the published procedure [1] which was modified. The reaction yielded a mixture of isomers (unsubstituted β-CD, product **1**, and multiply substituted isomers). Pure **1** was obtained after repeated (3×) crystallization in 10-fold excess (m/v) of 50% MeOH. Overall yield of the reaction was 26% of pure monotosylated isomer **1**.

m.p. 170 °C (starts to decompose);

$[\alpha]^{25}_{D}$ +127.2° (DMSO);

IR (KBr): 3342 cm^{-1} ν(O-H), 2933 cm^{-1} ν(C-H), 1402 cm^{-1} δ(C-H), 1369 cm^{-1} δ(C-H), 1155 cm^{-1} ν(C-O), 1026 cm^{-1} ν(C-O).

^{1}H NMR (600 MHz, DMSO-d_6): δ = 7.75 (d, J=8.1 Hz, 1H, H-2'), 7.43 (d, J=8.1 Hz, 1H, H-3'), 5.81 – 5.63 (m, 14H, 7 × OH-2, 7 × OH-3), 4.84 - 4.76 (m, 7H, 7 × H-1), 4.49 – 4.34 (m, 6H, 6 × OH-6), 4.33 (d, J=10.3 Hz, 1H, H-$6a^{I}$), 4.19 (dd, J_1=11.0 Hz, J_2=6.4 Hz, 1H, H-$6b^{I}$), 3.71 – 3.20 (m, 49H, 7 × H-2, 7 × H-3, 7 × H-4, 7 × H-5, 12 × H-6, overlapped signal with H_2O), 2.43 (s, 3H, 3 × H-5') ppm.

HRMS: for $C_{49}H_{76}O_{37}S$ calcd: *m/z* 1288.3786 (for $[M + Na]^+$ calcd 1311.3678) , found 1311.3672 $[M + Na]^+$, Δ -0.44 ppm.

6^{I}-*O*-*p*-Toluenesulfonyl-α-cyclodextrin (2). Compound **2** was prepared according to the published procedure [2] which was modified. The reaction yielded a mixture of isomers (unsubstituted α-CD, product **2**, and multiply substituted isomers). Pure **2** was obtained after column chromatography on reverse phase C18 silica gel, with gradient elution (MeOH/H_2O mixture). Elution by 10% MeOH afforded about 40% of unreacted starting compound (α-CD) and fractions containing pure monosubstited **2** were eluted by 20–30% MeOH. Overall yield of the reaction was 32% (after subtracting the amount of recovered starting material).

m.p. 140 °C (starts to decompose);

$[\alpha]^{25}_{D}$ +116.1° (DMSO);

IR (KBr): 3324 cm^{-1} ν(O-H), 2926 cm^{-1} ν(C-H), 1413 cm^{-1} δ(C-H), 1362 cm^{-1} δ(C-H), 1159 cm^{-1} ν(C-O), 1039 cm^{-1} ν(C-O).

^{1}H NMR (600 MHz, DMSO-d_6): δ = 7.77 (d, J=8.8 Hz, 2H, H-2'), 7. 45 (d, J=8.2 Hz, 2H, H-3'), 5.46 (*br s*, 12H, 6 × OH-2, 6 × OH-3), 4.80 – 4.79 (4 × H-1), 4.72 (d, J=3.1 Hz, 1H, H-1^{I}), 4.66 (d, J=3.1 Hz, 1H, H-1), 4.31 – 4.26 (m, 2H, 2 × H-6^{I}), 3.87 – 3.84 (m, 1H, H-5^{I}), 3.79 – 3.24 (m, 36H, 6 × H-2, 6 × H-3, 6 × H-4, 5 × H-5, 10 × H-6, overlapped signal with H_2O), 3.17 (dd, J_1=10.00 Hz, J_2=3.3 Hz, 1H, H-2^{I}), 2.41 (s, 3H, H-5') ppm.

^{13}C NMR (125 MHz, DMSO-d_6): δ = 144.76 (C-4'), 132.42 (C-1'), 129.91 (2 × C-3'), 127.67 (2 × C-2'), 102.05 – 101.89 (5 × C-1), 101.50 (C-1^{I}), 82.07 – 81.71 (5 × C-4), 81.69 (C-4^{I}), 73.22 – 71.59 (6 × C-2, 6 × C-3, 5 × C-5), 69.63 (C-6^{I}), 68.87 (C-5^{I}), 59.91 – 59.76 (5 × C-6), 21.12 (C-5') ppm.

HRMS: for $C_{43}H_{66}O_{32}S$ calcd: *m/z* 1126.3258 (for $[M + Na]^+$ calcd 1149.3150) , found 1149.3145 $[M + Na]^+$, Δ -0.42 ppm.

6^{I}-*O*-*p*-Toluenesulfonyl-γ-cyclodextrin (3). Compound was prepared according to the procedure described for the synthesis of **2**. Overall yield of the reaction was 50% (after subtracting the amount of recovered starting material).

m.p. 160 °C (starts to decompose);

$[\alpha]^{25}_{D}$ +134.3° (DMSO);

IR (KBr): 3297 cm^{-1} ν(O-H), 2932 cm^{-1} ν(C-H), 2905 cm^{-1} ν(C-H), 2833 cm^{-1} ν(C-H), 1416 cm^{-1}, δ(C-H), 1353 cm^{-1} δ(C-H), 1156 cm^{-1} ν(C-O), 1081 cm^{-1} ν(C-O), 1024 cm^{-1} ν(C-O).

^{1}H NMR (600 MHz, DMSO-d_6): δ = 7.77 (bd, J=7.7 Hz, 2H, H-2’), 7.45 (bd, J=8.5 Hz, 2H, H-3’), 5.74 (*br s*, 16 H, 8 × OH-2, 8 × OH-3), 4.90 – 4.88 (*m*, 6H, 6 × H-1), 4.81 – 4.80 (m, 2H, 2 × H-1), 4.51 (*br* s, 7H, 7 × OH-6), 4.30 (d, J=9.7 Hz, 1H, H-6a^{I}), 4.20 (dd, J_1=11.9 Hz, J_2=4.4 Hz, 1H, H-6b^{I}), 3.75 – 3.72 (m, 1H, H-5^{I}), 3.63 – 3.24 (m, 53H, 8 × H-2, 8 × H-3, 8 × H-4,7 × H-5, 14 × H-6, overlapped signal with H_2O), 2.42 (s, 3H, H-5’) ppm.

^{13}C NMR (125 MHz, DMSO-d_6): δ = 144.80 (C-4’), 132.53 (C-1’), 129.95 (2 × C-3’), 127.55 (2 × C-2’), 102.20 (C-1), 101.67 – 101.52 (6 × C-1), 101.09 (C-1), 81.08 (C-4), 80.95 (C-4), 80.95 (C-4), 80.92 (C-4), 80.82 (C-4), 80.78 (C-4), 80.69 (C-4), 80.09 (C-4), 72.71 – 72.04 (8 × C-2, 8 × C-3, 7 × C-5), 69.44 (C-6^{I}), 68.96 (C-5^{I}), 59.95 (5 × C-6), 59.69 (C-6), 59.35 (C-6), 21.57 (C-5’) ppm.

HRMS: for $C_{55}H_{86}O_{42}S$ calcd: *m/z* 1450.4314 (for $[M + Na]^+$ calcd 1473.4207), found 1473.4203 $[M + Na]^+$, Δ -0.22 ppm.

***N,N,N‘*-Trimethylethane-1,2-diamine (7).** Compound **7** was prepared according to the published procedure [3], using *N*-(2-chlorethyl)dimethylamine hydrochloride and $MeNH_2$ (40% solution in H_2O). Pure **7** was obtained by distillation of $CHCl_3$ extracts. Yield of the reaction 80%. For $C_5H_{14}N_2$ calcd: *m/z* 102.1, found ESI MS: 102,9 $[M]^+$. NMR spectra are in agreement with the literature [3].

***N,N,N‘*-Trimethylpropane-1,3-diamine (8).** Compound was prepared according to the published procedure [4], using *N*-(2-chlorpropyl)dimethylamine and $MeNH_2$ (40% solution in H_2O). Pure **8** was obtained by distillation of $CHCl_3$ extracts. Yield of the reaction 96%. For $C_6H_{16}N_2$ calcd: *m/z* 116.1, found ESI MS: 116.2 $[M]^+$. NMR spectra are in agreement with the literature [4].

6^I-(*N,N,N*-Trimethylammonio)-6^I-deoxy-β-cyclodextrin bicarbonate (4). Dry 6^I-*O*-*p*-toluenesulfonyl-β-cyclodextrin (**1**, 0.50 g, 0.39 mmol) was placed in a sealed tube with an aqueous solution of trimethylamine (45 wt %) (3.80 mL, 31.20 mmol). The mixture was stirred at 80 °C overnight. TLC of the reaction mixture showed two spots, one correspoding to the product and second which was assigned to 3,6-anhydro-β-cyclodextrin. The mixture was easily separated on a column (15 mL) of strong cation exchange resin in hydrogen form (Dowex 50, mesh 20–50). The column was firstly washed with H_2O (200 mL) to remove the byproduct 3,6-anhydro-β-cyclodextrin and then with aqueous NH_4HCO_3 3 wt % (300 mL) to elute the product. Salts were removed by thermal decomposition realized by repeated vacuum evaporation with H_2O (4 × 50 mL). Pure product **4** with bicarbonate anion was obtained by precipitation from acetone as fine white powder. Yield 71% (0.32 g).

m.p. 220 °C (starts to decompose);

$[\alpha]^{25}_D$ +118.9° (H_2O);

IR (KBr): 3303 cm^{-1} ν(O-H), 2926 cm^{-1} ν(C-H), 2911 cm^{-1} ν(C-H), 2833 cm^{-1} ν(C-H), 1416 cm^{-1}, δ (C-H), 1362 cm^{-1} δ(C-H), 1299 cm^{-1} δ(C-H), 1156 cm^{-1} ν(C-O), 1081 cm^{-1} ν(C-O), 1033 cm^{-1} ν(C-O).

^{1}H NMR (600 MHz, D_2O): δ = 5.11 -5.01 (m, 7H, 7 × H-1), 4.38 (t, J=8.8 Hz, 1H, H-5^I), 4.04 (bt, J=9.7 Hz, 1H, H-3^I), 4.02– 3.45 (m, 39H, 7 × H-2, 6 × H-3, 6 × H-4, 6 × H-5, 14 × H-6), 3.51 (bt, J=9.1 Hz, 1H, H-4^I), 3.20 (s, 9H, 3 × N^+-CH_3) ppm.

^{13}C NMR (125 MHz, D_2O): δ = 166.90 (HCO_3^-), 103.71 (C-1), 103.42 (C-1), 103.36 (2 × C-1), 103.30 (C-1), 103.13 (C-1), 102.00 (C-1), 84.62 (C-4^I), 83.33 (C-4), 89.92 (C-4), 82.81 (C-4), 82.54 (C-4), 82.39 (C-4), 80.86 (C-4), 74.72 – 73.12 (7 × C-2, 7 × C-3, 6 × C-5), 68.77 (C-5^I), 67.95 (C-6^I), 62.44 (C-6), 62.01 (C-6), 61.86 (C-6), 61.83 (C-6), 61.69 (C-6), 61.67 (C-6), 55.88 (3 × N^+-CH_3) ppm.

HRMS: for $C_{45}H_{78}O_{34}N$ calcd: *m/z* 1176.4400, found 1176.4395 $[M]^+$, Δ -0.39 ppm.

6^{I}-(*N,N,N*-Trimethylammonio)-6^{I}-deoxy-α-cyclodextrin bicarbonate (5). Compound **5** was prepared by the procedure described for the synthesis of **4**. The reaction of starting compound **2** (0.50 g, 0.44 mmol) with aqueous trimethylamine (45 wt %) (4.42 mL, 35.20 mmol) gave the product **5** (0.26 g) as white powder in 58% yield.

m.p. 190 °C (starts to decompose);

$[\alpha]^{25}_{D}$ +121.3° (H_2O);

IR (KBr): 3303 cm^{-1} ν(O-H), 2929 cm^{-1} ν(C-H), 1476 cm^{-1}, δ (C-H), 1404 cm^{-1} δ(C-H), 1362 cm^{-1} δ(C-H), 1293 cm^{-1} δ(C-H), 1150 cm^{-1} ν(C-O), 1081 cm^{-1} ν(C-O), 1042 cm^{-1} ν(C-O).

^{1}H NMR (600 MHz, D_2O): δ = 5.14 (d, J = 2.9 Hz, 1H, H-1^{I}), 5.10 (m, 2H, 2 × H-1), 5.08 (d, J = 3.3 Hz, 1H, H-1), 5.05 (d, J = 3.4 Hz, 1H, H-1), 5.03 (d, J = 3.4 Hz, 1H, H-1), 4.42 (dt, J_1 = 9.1 Hz, J_2 = 2.6 Hz, 1H, H-5^{I}), 4.10 – 3.49 (m, 35H, 6 × H-2, 6 × H-3, 6 × H-4, 5 × H-5, 12 × H-6), 3.24 (s, 9H, 3 × N-CH_3) ppm.

^{13}C NMR (125 MHz, D_2O): δ = 162.54 (HCO_3^-), 103.63 (C-1), 103.37 (C-1), 103.25 (C-1), 103.20 (C-1), 103.11 (C-1), 102.87 (C-1), 85.26 (C-4), 83.58 (C-4), 83.31 (C-4), 83.12 (C-4), 83.04 (C-4), 82.70 (C-4), 75.39 – 73.17 (6 × C-2, 6 × C-3, 5 × C-5), 69.86 (C-5^{I}), 68.38 (C-6^{I}), 63.00 (C-6), 62.75 (C-6), 62.64 (C-6), 62.43 (C-6), 62.26 (C-6), 56.43 (3 × N-CH_3) ppm.

HRMS: for $C_{39}H_{68}O_{29}N$ calcd: *m/z* 1014.3872, found 1014.3869 $[M]^+$, Δ -0.24 ppm.

6^{I}-(*N,N,N*-Trimethylammonio)-6^{I}-deoxy-γ-cyclodextrin bicarbonate (6). Compound **6** was prepared by the procedure described for the synthesis of **4**. The reaction of starting compound **3** (0.50 g, 0.34 mmol) with aqueous trimethylamine (45 wt %) (3.40 mL, 27.20 mmol) gave the product **5** (0.25 g) as white powder in 54% yield.

m.p. 200 °C (starts to decompose);

$[\alpha]^{25}_{D}$ +134.9° (H_2O);

IR (KBr): 3318 cm^{-1} ν(O-H), 2932 cm^{-1} ν(C-H), 1476 cm^{-1}, δ (C-H), 1410 cm^{-1} δ(C-H), 1374 cm^{-1} δ(C-H), 1293 cm^{-1} δ(C-H), 1159 cm^{-1} ν(C-O), 1081 cm^{-1} ν(C-O), 1030 cm^{-1} ν(C-O).

^{1}H NMR (600 MHz, D_2O): δ = 5.17 – 5.10 (m, 8H, 8 × H-1), 4.51 (ddd, J_1=9.9 Hz, J_2=6.6 Hz, J_3=3.5 Hz, 1H, H-5^I), 4.06 (t, J=9.3 Hz, 1H, H-3^I), 3.99 – 3.57 (m, 44H, 8 × H-2. 7 × H-3, 6 × H-4, 7 × H-5, 16 × H-6), 3.55 – 3.49 (m, 2H, H-4, H-4^I), 3.23 (s, 9H, 3 × N-CH_3) ppm.

^{13}C NMR (125 MHz, D_2O): δ = 167.40 (HCO_3^-), 104.01 (C-1), 103.84 (C-1), 103.67 (2 × C-1), 103.62 (C-1), 103.42 (C-1), 103.36 (C-1^I), 101.61 (C-1^I), 84.47 (C-4^I), 83.34 (C-4), 82.71 (C-4), 82.47 (C-4), 82.37 (C-4), 82.32 (C-4), 82.10 (C-4), 79.81 (C-4), 75.13 – 73.57 (8 × C-2, 8 × C-3, 7 × C-5), 68.83 (C-5^I), 68.62 (C-6^I), 63.00 (C-6), 62.39 (2 × C-6), 62.24 (C-6), 62.17 (C-6), 62.13 (C-6), 62.03 (C-6), 56,32 (3 × N-CH_3) ppm.

HRMS: for $C_{51}H_{88}O_{39}N$ calcd: *m/z* 1338.4928, found 1338.4923 $[M]^+$, Δ -0.39 ppm.

6^I-((2-(Dimethylamino)-1-(methyl)ethyl)amino)-6^I-deoxy-β-cyclodextrin (9). Compound **9** was prepared according to the published procedure [5] which was modified. Dry monotosylate **1** (1.50 g, 1.165 mmol) was dissolved in neat **7** (3.57 g, 4,5 mL, 34.950 mmol) with stirring, under inert atmosphere of argon. After complete dissolution (10 min) the reaction mixture was heated to 70 °C and stirred overnight under a weak stream of argon. The mixture changed the color to dark brown after completion. Unreacted **7** was then recovered by vacuum distillation at rt. The foamy brown residue was dissolved in minimum amount of H_2O (4 mL) and was precipitated by dropwise addition to *n*-propanol (60 mL). The suspension was then refluxed for 90 min, cooled and vacuum-filtered on a glass frit. The fine white precipitate was dried at rt overnight and finally in vacuo at 70 °C. The reaction yielded 1.31 g (93%) of **9** as a white powder.

m.p. 230 °C (starts to decompose);

$[\alpha]^{25}_D$ +134.2° (DMSO);

IR (KBr): 3283 cm^{-1} ν(O-H), 2930 cm^{-1} ν(C-H), 2824 cm^{-1} ν(C-H), 2786 cm^{-1} ν(C-H), 1457 cm^{-1} δ(C-H), 1364 cm^{-1} δ(C-H), 1154 cm^{-1} ν(C-O), 1076 cm^{-1} ν(C-O), 1026 cm^{-1} ν(C-O).

^{1}H NMR (600 MHz, DMSO-*d$_6$*): δ = 5.94 – 5.64 (m, 14H, 7 × OH-2, 7 × OH-3), 4.87 – 4.79 (m, 7H, 7 × H-1), 4.72 – 4.43 (m, 6H, 6 × OH-6), 3.75 – 3.28 (m, 46H, 7 × H-2, 7 × H-3, 6 × H- 4, 7 × H-5, 12 × H-6, overlapped signal with H_2O), 3.17 (bt, J = 9.4 Hz, 1H, H-4^{I}), 2.69 (m, 1H, H-6a^{I}), 2.45 (m, 2H, H-1a', H-6b^{I}, overlapped signal with DMSO), 2.38 (m, 1H, H-1b'), 2.26 (m, 2H, H-2'), 2.17 (s, 3H, N-1-CH_3), 2.07 (s, 6H, 2 × N-2-CH_3) ppm.

^{13}C NMR (125 MHz, DMSO-*d$_6$*): δ = 102.14 – 101.54 (7 × C-1), 84.11 (C-4^{I}), 81.53 – 79.16 (6 × C-4), 73.33 – 71.89 (7 × C-2, 7 × C-3, 7 × C-5), 70.28 (C-5^{I}), 59.83 – 59.70 (6 × C-6), 58.36 (C-6^{I}), 56.96 (C-2'), 55.89 (C-1'), 45.39 (2 × N-2-CH_3), 43.22 (N-1-CH_3) ppm.

HRMS: for $C_{47}H_{82}O_{34}N_2$ calcd: *m/z* 1218.4749 (for $[M + H]^+$ calcd 1219.4822), found 1219.4818 $[M + H]^+$, Δ -0.31 ppm.

6^{I}((2-(Dimethylamino)-1-(methyl)ethyl)amino)-6^{I}-deoxy-α-cyclodextrin (10). Compound **10** was prepared by the procedure for preparation of **9** which was modified. Dry monotosylate **2** (0.20 g, 0.178 mmol) was dissolved in neat **7** (0.62 g, 0.8 mL, 5.32 mmol) with stirring, under inert atmosphere of argon. After complete dissolution (10 min) the reaction mixture was heated to 70 °C and stirred overnight under a weak stream of argon. The mixture changed the color to dark brown after completion. Unreacted **7** was then recovered by vacuum distillation at rt. The foamy brown residue was dissolved in minimum amount of H_2O (1.0 mL) and was precipitated by dropwise addition to acetone (30 mL). The mixture was then refluxed for 1 h, cooled and vacuum-filtered on a glass frit. The crude white solid was superficially dried, dissolved in H_2O (20 mL) and applied to a column (10 mL) of strong cation exchanger resin in hydrogen form (Dowex 50, mesh 20–50). The column was firstly washed with H_2O (100 mL) to remove TsOH and then with 1 M NH_4OH (100 mL) to elute

the product. This solution was evaporated under reduced pressure. The solid residue was dissolved in minimum amount of H_2O and precipitated by addition of acetone (30 mL). The white precipitate was collected on a glass frit by vacuum filtration and was dried at rt overnight and finally in vacuo at 70 °C. The reaction yielded 0.14 g of **10** as fine white powder in 69% yield.

m.p. 210 °C (starts to decompose);

$[\alpha]^{25}_{D}$ +114.2° (H_2O);

IR (KBr): 3312 cm^{-1} ν(O-H), 2935 cm^{-1} ν(C-H), 2899 cm^{-1} ν(C-H), 2830 cm^{-1} ν(C-H), 2794 cm^{-1} ν(C-H), 1455 cm^{-1} δ(C-H), 1416 cm^{-1} δ(C-H), 1368 cm^{-1} δ(C-H), 1332 cm^{-1} δ(C-H), 1150 cm^{-1} ν(C-O), 1081 cm^{-1} ν(C-O), 1039 cm^{-1} ν(C-O).

^{1}H NMR (600 MHz, D_2O): δ = 5.06 – 5.03 (m, 6H, 6 × H-1), 3.99 – 3.80 (m, 22H, 6 × H-3, 6 × H-5, 10 × H-6), 3.63 – 3.56 (m, 11H, 6 × H-2, 5 × H-4), 3.40 (bt, J=9.2 Hz, 1H, H-4^{I}), 2.90 (d, J=13.0 Hz, 1H, H-6a^{I}), 2.71 – 2.58 (m, 5H, H-6b^{I}, 2 × H-1', 2 × H-2'), 2.29 – 2.28 (m, 9H, N-1-$CH_{3,}$ 2 × N-2-$CH_{3)}$ ppm.

^{13}C NMR (125 MHz, D_2O): δ = 103.21 (C-1), 103.16 (C-1), 103.14 (C-1), 103.11 (C-1), 103.05 (C-1), 102.94 (C-1), 85.82 (C-4^{I}), 82.92 (2 × C-4), 82.83 (C-4), 82.78 (C-4), 82.67 (C-4), 75.06 – 73.34 (6 × C-2, 6 × C-3, 5 × C-5), 71.61 (C-5^{I}), 62.18 – 62.02 (5 × C-6), 59.47 (C-6^{I}), 56.35 (C-1'), 55.77 (C-2'), 45.83 (2 × N-2-CH_3), 43.93 (N-1-CH_3) ppm.

HRMS: for $C_{41}H_{73}O_{29}N_2$ calcd: *m/z* 1057.4294, found 1057.4291 $[M]^+$, Δ -0.25 ppm.

6^{I}-((2-(Dimethylamino)-1-(methyl)ethyl)amino)-6^{I}-deoxy-γ-cyclodextrin (11). Compound **11** was prepared by the procedure described for the synthesis of **10**. Reaction of starting compound **3** (0.20 g, 0.138 mmol) with diamine **7** (0.42 g, 0.54 mL, 4.13 mmol) gave the product **11** (0.15 g) as white powder in 77% yield.

m.p. 220 °C (starts to decompose);

$[\alpha]^{25}_D$ +139.3° (H_2O);

IR (KBr): 3309 cm^{-1} ν(O-H), 2929 cm^{-1} ν(C-H), 2824 cm^{-1} ν(C-H), 2779 cm^{-1} ν(C-H), 1458 cm^{-1} δ(C-H), 1422 cm^{-1} δ(C-H), 1365 cm^{-1} δ(C-H), 1156 cm^{-1} ν(C-O), 1078 cm^{-1} ν(C-O), 1033 cm^{-1} ν(C-O).

^1H NMR (600 MHz, D_2O): δ = 5.21 – 5.10 (m, 8H, 8 × H-1), 4.04 (t, J=9.6 Hz, 1H, H-5^I), 3.98 – 3.82 (m, 29H, 8 × H-3, 7 × H-5, 14 × H-6), 3.68 – 3.58 (m, 15H, 8 × H-2, 7 × H-4), 3.40 (t, J=10.0 Hz, 1H, H-4^I), 2.89 (d, J=13.6 Hz, 1H, H-$6a^I$), 2.72 – 2.59 (m, 5H, H-$6b^I$, 2 × H-1', 2 × H-2'), 2.34 – 2.32 (s, 9H, N-1-CH_3, 2 × N-2-CH_3) ppm.

^{13}C NMR (125 MHz, D_2O): δ = 103.66 – 101.97 (8 × C-1), 83.96 (C-4^I), 82.43 (2 × C-4), 82.37 (C-4), 82.30 (C-4), 82.19 (C-4), 82.03 (C-4), 80.66 (C-4), 75.30 – 73.70 (8 × C-2, 8 × C-3, 7 × C-5), 71.11 (C-5^I), 62.24 - 61.96 (7 × C-6), 59.44 (C-6^I), 56.91 (C-2'), 55.35 (C-1'), 46.20 (2 × N-2-CH_3) , 44.49 (N-1-CH_3) ppm.

HRMS: for $C_{53}H_{93}O_{39}N_2$ calcd: *m/z* 1381.5350, found 1381.5344 $[M]^+$, Δ -0.41 ppm.

6^I-((3-(Dimethylamino)-1-(methyl)propyl)amino)-6^I-deoxy-β-cyclodextrin (15). Compound **15** was prepared by the procedure described for the synthesis of **9**. The reaction of starting compound **1** (2.00 g, 1.55 mmol) with diamine **8** (4.80 g, 6.0 mL, 41.4 mmol) gave the product **15** (2.18 g) as white powder in 99% yield.

m.p. 220 °C (starts to decompose);

$[\alpha]^{25}_D$ +127.3° (DMSO);

IR (KBr): 3303 cm^{-1} ν(O-H), 2935 cm^{-1} ν(C-H), 2797 cm^{-1} ν(C-H), 1414 cm^{-1} δ(C-H), 1332 cm^{-1} δ(C-H), 1155 cm^{-1} ν(C-O), 1079 cm^{-1} ν(C-O), 1029 cm^{-1} ν(C-O).

^{1}H NMR (600 MHz, DMSO-d_6): δ = 5.93 – 5.64 (m, 14H, 7 × OH-2, 7 × OH-3), 4.88 – 4.80 (m, 7H, 7 × H-1), 4.52 – 4.46 (m, 6H, 6 × OH-6), 3.81 – 3.30 (m, 39H, 7 × H-2, 7 × H-3, 6 × H-4, 7 × H-5, 12 × H-6) 3.19 (bt, J-9.2 Hz, 1H, H-4^I), 2.63 (m, 1H, H-6a^I), 2.44 – 2.38 (m, 2H, H-1a', H-6b^I, overlapped signal with DMSO), 2.25 – 2.06 (m, 12H, H-1b', 2 × H-3', N-1-CH_3, 2 × N-2-CH_3), 1.45 (m, 2H, 2 × H-2') ppm.

^{13}C NMR (125 MHz, DMSO-d_6) δ = 102.16 – 101.55 (7 × C-1), 84.13 (C-4^I), 81.55 – 81.06 (6 × C-4), 73.31 – 71.84 (7 × C-2, 7 × C-3, 6 × C-5), 70.44 (C-5^I), 59.80 – 59.57 (6 × C-6), 58.02 (C-6^I), 57.08 (C-3'), 55.81 (C-1'), 45.16 (2 ×,N-2-CH_3), 43.21 (N-1-CH_3), 24.93 (C-2') ppm.

HRMS: for $C_{48}H_{85}O_{34}N_2$ calcd: *m/z* 1232.4905 (for $[M + H]^+$ calcd 1233.4978), found 1233.4974 $[M + H]^+$, Δ -0.34 ppm.

6^I-((3-(Dimethylamino)-1-(methyl)propyl)amino)-6^I-deoxy-α-cyclodextrin (16). Compound **16** was prepared by the procedure described for the synthesis of **10**. The reaction of starting compound **2** (0.20 g, 0.178 mmol) with diamine **8** (0.69 g, 0.78 mL, 5.32 mmol) gave the product **16** (0.16 g) as white powder in 86% yield.

m.p. 210 °C (starts to decompose);

$[\alpha]^{25}_D$ +90.9° (H_2O);

IR (KBr): 3291 cm^{-1} ν(O-H), 2923 cm^{-1} ν(C-H), 2830 cm^{-1} ν(C-H), 1449 cm^{-1} δ(C-H), 1329 cm^{-1} δ(C-H), 1293 cm^{-1} δ(C-H), 1159 cm^{-1} ν(C-O), 1036 cm^{-1} ν(C-O).

^{1}H NMR (600 MHz, D_2O):): δ = 5.07 – 5.04 (m, 6H, 6 × H-1), 4.01 – 3.82 (m, 22H, 6 × H-3, 6 × H-5, 10 × H-6), 3.65 – 3.58 (m, 11H, 6 × H-2, 5 × H-4), 3.40 (bt, J=9.4 Hz, 1H, H-4^I), 2.85 (m, 1H, H-6a^I), 2.72 (dd, J_1=14.1, J_2=9.4 Hz, 1H, H-6b^I), 2.55 (bt, J=7.6 Hz, 2H, H-3'), 2.49 (m, 2H, H-1'), 2.38 (s, 6H, 2 × N-2-CH_3), 2.26 (s, 3H, N-1-CH_3), 1.74 (m, 2H, 2 × H-2') ppm.

^{13}C NMR (125 MHz, D_2O): δ = 103. 40 – 103.05 (5 × C-1), 103.05 (C-1 ^{I}), 86.09 (C-4 ^{I}), 83.18 (C-4), 83.11 (2 × C-4), 82.96 (C-4), 82.91 (C-4), 75.26 – 73.53 (6 × C-2 , 6 × C-3, 5 × C-5), 71.62 (C-5^{I}), 62.51– 62.22 (5 × C-6), 59.64 (C-6^{I}), 58.55 (C-3'), 57.58 (C-1'), 45.49 (2 × N-2-CH_3), 43.48 (N-1-CH_3), 24.33 (C-2') ppm.

HRMS: for $C_{42}H_{74}O_{29}N_2$ calcd: *m/z* 1070.4377 (for $[M + H]^+$ calcd 1071.4450), found 1071.4448 $[M + H]^+$, Δ -0,17 ppm.

6^{I}-((3-(Dimethylamino)-1-(methyl)propyl)amino)-6^{I}-deoxy-γ-cyclodextrin (17). Compound **17** was prepared by the procedure described for the synthesis of **10**. The reaction of starting compound **3** (0.30 g, 0.207 mmol) with diamine **8** (0.72 g, 0.90 mL, 6.20 mmol) gave the product **17** (0.23 g) as white powder in 78% yield.

m.p. 210 °C (starts to decompose);

$[\alpha]^{25}_D$ +132.6° (H_2O);

IR (KBr): 3318 cm^{-1} ν(O-H), 2938 cm^{-1} ν(C-H), 2821 cm^{-1} ν(C-H), 1458 cm^{-1} δ(C-H), 1410 cm^{-1} δ(C-H), 1368 cm^{-1} δ(C-H), 1329 cm^{-1} δ(C-H), 1156 cm^{-1} ν(C-O), 1081 cm^{-1} ν(C-O), 1030 cm^{-1} ν(C-O).

^{1}H NMR (600 MHz, D_2O):): δ = 5.20 - 5.08 (m, 8H, 8 × H-1), 4.04 (bt, J=9.6 Hz, 1H, H-5^{I}), 3.96 – 3.82 (m, 29H, 8 × H-3, 7 × H-5, 14 × H-6), 3.69 – 3.58 (m, 15H, 8 × H-2, 7 × H-4), 3.40 (bt, J=9.2 Hz, 1H, H-4^{I}), 2.88 (d, J=13.2 Hz, 1H, H-6a^{I}), 2.67 (dd, J_1=13.8 Hz, J_2=9.9 Hz, 1H, H-6b^{I}), 2.57 – 2.44 (m, 4H, 2 × H-1', 2 × H-3'), 2.33 (s, 6H, 2 × N-2-CH_3), 2.30 (s, 3H, N-1-CH_3), 1.72 – 1.67 (m, 2H, 2 × H-2') ppm.

^{13}C NMR (125 MHz, D_2O): δ = 103.70 (2 × C-1), 103.61 (C-1), 103.52 (2 × C-1), 103.41 (C-1), 103.37 (C-1), 102.10 (C-1^{I}), 84.52 (C-4^{I}), 82.43 (3 × C-4), 82.28 (C-4), 82.26 (C-4), 82.21 (C-4), 80.55 (C-4), 75.28 – 73.70 (m, 8 × C-2, 8 × C-3, 7 × C-5), 70.99 (C-5^{I}), 62.23 (2 × C-

6), 62.17 (C-6), 62.14 (C-6), 62.09 (2 × C-6),,61.96 (C-6), 58.72 (C-6^I), 58.72 (C-3’), 56.09 (C-1’), 45.88 (2 × N-2-CH_3), 44.21 (N-1-CH_3), 24.29 (C-2’) ppm.

HRMS: for $C_{54}H_{94}O_{39}N_2$ calcd: *m/z* 1394.5434 (for $[M + H]^+$ calcd 1395.5507), found 1395.5503 $[M + H]^+$, Δ -0.22 ppm.

6^I-(*N,N,N’,N‘,N‘*-Pentamethylethane-1,2-diammonio)-6^I-deoxy-β-cyclodextrin diiodide (12). Starting compound **9** (0.50 g, 0.41 mmol) was dissolved in dry DMF (5.0 mL) with stirring. MeI (0.87 g, 0.38 mL, 6.16 mmol) was then added dropwise via syringe. The reaction mixture was stirred for 18 h under innert atmosphere of argon at 30 °C. The reaction mixture changed the color from colorless to light yellow. Completeness of the reaction was checked by TLC (MeOH/HOAc-1% solution of NH_4OAc in H_2O 10:1:9). The solvent was removed in vacuo, the solid residue was dissolved in H_2O (2 mL) and precipitated from acetone (25 mL). The precipitate was filtered on a glass frit and dried in vacuo. The reaction afforded product **12** (0.60 g) in the diiodide form, as white powder in excellent yield (98%).

m.p. 230 °C (starts to decompose);

$[\alpha]^{25}_D$ +104.3° (H_2O);

IR (KBr): 3312 cm^{-1} ν(O-H), 2926 cm^{-1} ν(C-H), 1419 cm^{-1} δ(C-H), 1365 cm^{-1} δ(C-H), 1329 cm^{-1} δ(C-H), 1296 cm^{-1} δ(C-H), 1159 cm^{-1} ν(C-O), 1078 cm^{-1} ν(C-O), 1030 cm^{-1} ν(C-O).

^1H NMR (600 MHz, D_2O):): δ = 5.19 (d, J = 2.3 Hz, 1H, H-1^I), 5.14 (d, J = 3.4 Hz, 1H, H-1), 5.10 – 5.03 (m, 5H, 5 × H-1), 4.39 (m, 1H, H-5^I), 4.16 – 3.56 (m, 44H, 7 × H-2, 7 × H-3, 6 × H-4, 6 × H-5, 14 × H-6, 2 × H-1’, 2 × H-2’), 3.47 (bt, J = 9.7 Hz, 1H, H-4), 3.38 (s, 3H, N-1-CH_3), 3.35 (s, 3H, N-1-CH_3), 3.29 (s, 9H, 3 × N-2-CH_3) ppm.

^{13}C NMR (125 MHz, D_2O): δ = 104.15 (C-1), 103.92 (2 × C-1), 103.85 (C-1), 103.72 (2 × C-1), 102.86 (C-1 ^{I}), 84.98 (C-4 ^{I}), 83.73 (C-4), 83.21 (C-4), 83.15 (C-4), 82.99 (C-4), 82.89 (C-

4), 81.67 (C-4), 75.18 – 73.31 (7 × C-2 , 7 × C-3, 6 × C-5), 69.63(C-5^{I}), 66.33 (C-6^{I}), 63.03–62.07 (6 × C-6), 60.32 (C-1'), 59.52 (C-2'), 55.83 (3 × N-2-CH_3), 54.91 (N-1-CH_3), 54.85 (N-1-CH_3) ppm.

HRMS: for $C_{49}H_{88}O_{34}N_2$ calcd: *m/z* 1248.5028 (for $[M]^{2+}$ calcd 624.2604), found 624.2604 $[M]^{2+}$, Δ -0.08 ppm.

6^{I}-(*N,N,N',N',N'*-Pentamethylethane-1,2-diammonio)-6^{I}-deoxy-α-cyclodextrin diiodide (13). Compound **13** was prepared by the procedure described for the synthesis of **12**. The reaction of starting compound **10** (0.050 g, 0.047 mmol) with MeI (0.11 g, 0.04 mL, 0.710 mmol) gave the product **13** (0.056 g) as white powder in 88% yield.

m.p. 220 °C (starts to decompose);

$[\alpha]^{25}_{D}$ +92.5° (H_2O);

IR (KBr): 3336 cm^{-1} ν(O-H), 2932 cm^{-1} ν(C-H), 1488 cm^{-1} δ(C-H), 1416 cm^{-1} δ(C-H), 1329 cm^{-1} δ(C-H), 1293 cm^{-1} δ(C-H), 1156 cm^{-1} ν(C-O), 1078 cm^{-1} ν(C-O), 1036 cm^{-1} ν(C-O).

^{1}H NMR (600 MHz, D_2O):): δ = 5.16 (d, J=2.9 Hz, 1H, H-1^{I}), 5.08 – 4.99 (m, 5H, 5 × H-1), 4.87 (bt, J=9.4 Hz, 1H, H-5^{I}), 4.34 – 3.57 (m, 38H, 6 × H-2, 6 × H-3, 5 × H-4, 5 × H-5, 12 × H-6, 2 × H-1', 2 × H-2'), 3.47 (bt, J=9.2 Hz, 1H, H-4), 3.42 (s, 3H, N-1-CH_3), 3.37 (s, 3H, N-1-CH_3), 3.30 (s, 9H, 3 × N-2-CH_3) ppm.

^{13}C NMR (125 MHz, D_2O): δ = 103.67 (C-1), 103.52 (C-1), 103.40 (2 × C-1), 103.37 (C-1), 103.20 (C-1), 85.32 (C-4^{I}), 83.83 (C-4), 83.21 (C-4), 83.21 (C-4), 83.09 (C-4), 82.94 (C-4), 75.38 – 73.27 (6 × C-2, 6 × C-3, 5 × C-5), 70.25 (C-5^{I}), 66.80 (C-6^{I}), 63.18 - 62.43 (5 × C-6), 60.02 (C-2'), 59.68 (C-1'), 55.94 (N-1-CH_3), 55.90 (3 × N-2-CH_3), 55.46 (N-1-CH_3) ppm.

HRMS: for $C_{43}H_{78}O_{29}N_2$ calcd: *m/z* 1086.4679 (for $[M]^{2+}$ calcd 543.2340), found 543.2340 $[M]^{2+}$, Δ 0.02 ppm.

6^{I}-(*N,N,N',N',N'*-Pentamethylethane-1,2-diammonio)-6^{I}-deoxy-γ-cyclodextrin diiodide (14). Compound **14** was prepared by the procedure described for the synthesis of **12**. The reaction of starting compound **11** (0.117 g, 0.085 mmol) with MeI (0.18 g, 0.08 mL, 1.271 mmol) gave the product **14** (0.112 g) as white powder in 80% yield.

m.p. 230 °C (starts to decompose);

$[\alpha]^{25}_{D}$ +107.2° (H_2O);

IR (KBr): 3330 cm^{-1} ν(O-H), 2935 cm^{-1} ν(C-H), 1410 cm^{-1} δ(C-H), 1377 cm^{-1} δ(C-H), 1329 cm^{-1} δ(C-H), 1299 cm^{-1} δ(C-H), 1156 cm^{-1} ν(C-O), 1081 cm^{-1} ν(C-O), 1027 cm^{-1} ν(C-O).

^{1}H NMR (600 MHz, D_2O):): δ = 5.25 (d, J=3.0 Hz, 1H, H-1^{I}), 5.17 - 5.10 (m, 7H, 7 × H-1), 4.65 (bt, J=9.6 Hz, 1H, H-5^{I}), 4.15 – 3.60 (m, 50H, 8 × H-2, 8 × H-3, 7 × H-4, 7 × H-5, 16 × H-6, 2 × H-1', 2 × H-2'), 3.50 (bt, J=9.4 Hz, 1H, H-4), 3.37 – 3.36 (m, 6H, 2 × N-1-CH_3),s 3.30 (s, 9H, 3 × N-2-CH_3) ppm.

^{13}C NMR (125 MHz, D_2O): δ = 103.77 (C-1), 103.64 (C-1), 103.56 (2 × C-1), 103.39 (2 × C-1), 103.35 (C-1), 101.64 (C-1^{I}), 84.36 (C-4^{I}), 82.90 (C-4), 82.37 (C-4), 82.33 (C-4), 82.25 (2 ×C-4), 82.09 (C-4), 79.65 (C-4), 75.10 – 73.45 (8 × C-2, 8 × C-3, 7 × C-5), 68.84 (C-5^{I}), 67.09 (C-6^{I}), 63.19 (C-6), 62.41 – 62.18 (6 × C-6), 60.25 (C-1'), 59.67 (C-2'), 55.94 (3 × N-CH_3), 54.94 (N-CH_3), 54.49 (N-CH_3) ppm.

HRMS: for $C_{55}H_{98}O_{39}N_2$ calcd: *m/z* 1410.5736 (for $[M]^{2+}$ calcd 705.2868), found 705.2868 $[M]^{2+}$, Δ 0.03 ppm.

6^{I}-(*N,N,N',N',N'*-Pentamethylpropane-1,3-diammonio)-6^{I}-deoxy-β-cyclodextrin diiodide (18). Compound **18** was prepared by the procedure described for the synthesis of **12**. The reaction of starting compound **15** (5.00 g, 4.053 mmol) with MeI (8.63 g, 3.80 mL, 60.803 mmol) gave the product **18** (6.21 g) as white powder in 98% yield.

m.p. 240 °C (starts to decompose);

$[\alpha]^{25}_{D}$ +106.4° (H_2O);

IR (KBr): 3326 cm^{-1} ν(O-H), 2926 cm^{-1} ν(C-H), 1473 cm^{-1} δ(C-H), 1407 cm^{-1} δ(C-H), 1332 cm^{-1} δ(C-H), 1159 cm^{-1} ν(C-O), 1078 cm^{-1} ν(C-O), 1033 cm^{-1} ν(C-O).

^{1}H NMR (600 MHz, D_2O):): δ = 5.13 – 5.02 (m, 7H, 7 × H-1, 4.50 – 4.44 (m, 1H, H-5^{I}), 4.08 – 3.35 (m, 45H, 7 × H-2, 7 × H-3, 7 × H-4, 6 × H-5, 14 × H-6, 2 × H-1', 2 × H3'), 3.23 – 3.22 (m, 6H, 2 × N-1-CH_3), 3.18 (s, 9H, 3 × N-2-CH_3), 2.39 (m, 2H, 2 × H-2') ppm.

^{13}C NMR (125 MHz, D_2O): δ = 103.85 – 103.31 (6 × C-1), 102.31 (C-1 ^{I}), 84.82 (C-4 ^{I}), 83.47 (C-4), 82.91(2 × C-4), 82.75 (C-4), 82.61 (C-4), 81.12 (C-4), 74.90 – 73.20 (7 × C-2 , 7 × C-3, 6 × C-5), 68.99 (C-5^{I}), 66.51 (C-6^{I}), 64.42 (C-1'), 63.98 (C-3'), 62.73– 61.95 (6 × C-6), 54.84 (3 × N-2-CH_3), 53.70 (N-1-CH_3), 53.00 (N-1-CH_3), 18.80 (C-2') ppm.

HRMS: for $C_{50}H_{90}O_{34}N_2$ calcd: *m/z* 1262.5364 (for $[M]^{2+}$ calcd 631.2682), found 631.2682 $[M]^{2+}$, Δ 0.07 ppm.

6^{I}-(*N,N,N',N',N'*-Pentamethylpropane-1,3-diammonio)-6^{I}-deoxy-α-cyclodextrin diiodide (19). Compound **19** was prepared by the procedure described for the synthesis of **12**. The reaction of starting compound **16** (0.050 g, 0.047 mmol) with MeI (0.10 g, 0.05 mL, 0.700 mmol) gave the product **19** (0.052 g) as white powder in 83% yield.

m.p. 240 °C (starts to decompose);

$[\alpha]^{25}_{D}$ +92.3° (H_2O);

IR (KBr): 3327 cm^{-1} ν(O-H), 2926 cm^{-1} ν(C-H), 1482 cm^{-1} δ(C-H), 1329 cm^{-1} δ(C-H), 1293 cm^{-1} δ(C-H), 1159 cm^{-1} ν(C-O), 1078 cm^{-1} ν(C-O), 1030 cm^{-1} ν(C-O).

^1H NMR (600 MHz, D_2O):): 5.14 – 5.00 (m, 6H, 6 × H-1), 4.77 (overlapped signal with H_2O, 1H, H-5^I), 4.29 – 3.75 (m, 23H, 6 × H-3, 5 × H-5, 12 × H-6), 3.66 – 3.46 (m, 16H, 6 × H-2, 6 × H-4, 2 × H-1', 2 × H-3'), 3.31 (s, 3H, N-1-CH_3), 3.28 (s, 3H, N-1-CH_3), 3.20 (s, 9H, 3 × N-2-CH_3), 2.41 (m, 2H, H-2') ppm.

^{13}C NMR (125 MHz, D_2O): δ = 103.70 (C-1), 103.46 (C-1), 103.37 (C-1), 103.37 (C-1), 103.37 (C-1), 103.23 (C-1), 85.36 (C-4^I), 83.61 (C-4), 83.23 (C-4), 83.15 (C-4), 83.10 (C-4), 83.04 (C-4), 75.42 – 73.33 (6 × C-2, 6 × C-3, 5 × C-5), 70.12 (C-5^I), 67.45 (C-6^I), 64.41 (C-1'), 64.35 (C-3'), 62.98 (C-6), 62.90 (C-6), 62.61 (C-6), 62.49 (C-6), 62.42 (C-6), 55.16 (3 × N-2-CH_3), 54.67 (N-1-CH_3), 54.30 (N-1-CH_3), 19.19 (C-2') ppm.

HRMS: for $C_{44}H_{80}O_{29}N_2$ calcd: *m/z* 1100.4836 (for $[M]^{2+}$ calcd 550.3418), found 550.2418 $[M]^{2+}$, Δ 0.07 ppm.

6^I-(*N,N,N',N',N'*-Pentamethylpropane-1,3-diammonio)-6^I-deoxy-γ-cyclodextrin diiodide (20). Compound **20** was prepared by the procedure described for the synthesis of **12**. The reaction of starting compound **17** (0.100 g, 0.072 mmol) with MeI (0.15 g, 0.07 mL, 1.075 mmol) gave the product **20** (0.082 g) as white powder in 80% yield.

m.p. 240 °C (starts to decompose);

$[\alpha]^{25}_D$ +117.6° (H_2O);

IR (KBr): 3330 cm^{-1} ν(O-H), 2935 cm^{-1} ν(C-H), 1479 cm^{-1} δ(C-H), 1404 cm^{-1} δ(C-H), 1374 cm^{-1} δ(C-H), 1335 cm^{-1} δ(C-H), 1156 cm^{-1} ν(C-O), 1078 cm^{-1} ν(C-O), 1030 cm^{-1} ν(C-O).

^1H NMR (600 MHz, D_2O):): δ = 5.20 – 5.10 (m, 8H, 8 × H-1), 4.59 (t, J=9.5 Hz, 1H, H-5^I), 4.09 (t, J=9.3 Hz, 1H, H-3^I), 4.01 – 3.43 (m, 50 H, 8 × H-2. 7 × H-3, 8 × H-4, 7 × H-5, 16 × H-6, 2 × H-1', 2 × H-3'). 3.27 (s, 6H, 2 × N-CH_3), 3.22 (s, 9H, 3 × N-CH_3), 2.45 – 2.39 (m, 2H, 2 × H-2') ppm.

^{13}C NMR (125 MHz, D_2O): δ = 103.91 (C-1), 103.74 (C-1), 103.64 (C-1), 103.60 (C-1), 103.41 (2 × C-1), 103.34 (C-1), 101.61(C-1^I), 84.49 (C-4^I), 83.11 (C-4), 82.44 (C-4), 82.36 (C-4), 82.28 (C-4), 82.26 (C-4), 82.01 (C-4), 79.58 (C-4), 75.15 – 73.41 (8 × C-2, 8 × C-3, 7 × C-5), 68.76 (C-5^I), 67.47 (C-6^I), 64.39 (C-1'*), 64.33 (C-3'*), 63.10 (C-6), 62.44 (2 × C-6), 62.29 (C-6), 62.24 (C-6), 62.21 (C-6), 62.17 (C-6), 55.27 (3 × N-2-CH_3), 54.05 (N-1-CH_3), 53.53 (N-1-CH_3), 19.24 (C-2') ppm. (signals * could be interchanged)

HRMS: for $C_{56}H_{100}O_{39}N_2$ calcd: *m/z* 1424.5892 (for $[M]^{2+}$ calcd 712.2946), found 712.2945 $[M]^{2+}$, Δ -0.13 ppm.

2-Hydroxyethyl-*p*-toluenesulfonate (21). *p*-Toluenesulfonyl chloride (10.0 g, 0.052 mmol) was added to ethylene glycol (180 mL, 3.23 mmol) with stirring. The mixture was stirred for 30 min at rt. Triethylamine (7.24 mL, 0.052 mmol) was then added dropwise in the course of 5 min and the reaction mixture's temperature has raised spontaneously to approximately 40 °C. Course of the reaction was monitored by TLC (hexane/EtOAc 1:1) and detected with (UV light 254 nm and subsequent dipping in solution of 0.5% $KMnO_4$ and 4% NaOH in H_2O). After 1 h the reaction was complete. The mixture was transferred to a separatory funnel with H_2O (400 mL) and $CHCl_3$ (400 mL). After shaking, the organic phase was separated and the H_2O phase was extracted with another 200 mL of $CHCL_3$. Organic extracts were merged, dried with anhydrous $MgSO_4$ and evaporated in vacuo. The reaction afforded product **21** (10.04 g) as clear colorless viscous liquid in 92% yield.

for $C_9H_{12}O_4S$ calcd: *m/z* 216.1, ESI-MS found 239.0 $[M + Na]^+$.

NMR spectra are in agreement with published data[6]

2-Azidoethanol (22). The compound was prepared according to the procedure published in literature [7]. Compound **21** (11.73 g, 0.054 mmol) was suspended in H_2O (30 mL) and sodium azide (4.24 g, 0.065 mmol) was added. The reaction mixture was refluxed at 115 °C.

After 2 h all the components of the reaction were dissolved. The reaction was complete after 20 h, which was determined by TLC (hexane/ethyl acetate 1:1). The reaction mixture was saturated by anhydrous $MgSO_4$ and the product extracted into CH_2Cl_2 (3 × 10 mL), which was previously purified by distillation with P_2O_5 (to remove ethanol which is used as stabilizing agent). CH_2Cl_2 extracts were merged and dried with anhydrous $MgSO_4$. The product was obtained as CH_2Cl_2 solution and wasn't further isolated and characterized due to its potentially explosive character and was used directly in next reaction step. TLC (hexane-ethyl acetate 1:1) of the solution showed one spot with R_f = 0.5.

2-Azidoethyl-4-methylbenzensulfonate (23). The compound was prepared according to the published procedure [7], using the CH_2Cl_2 solution of **22** as starting material. Product was purified on silica gel column with isocratic elution by hexane/ethyl acetate 5:1. Yield of the reaction was 46%. NMR spectra are in agreement with literature [7].

1-Azido-2-iodoethane (24). The compound was prepared by Finkelstein reaction according to the published procedure [8], using compound **23** as starting material. Product was purified on silica gel column with isocratic elution by hexane/ethyl acetate 100:1. Yield of the reaction was 96%. NMR spectra are in agreement with literature [8].

6^I-(*N'*-(2-Azidoethyl)-*N,N,N',N'*-tetramethylethane-1,2-diammonio)-6^I-deoxy-β-cyclodextrin diiodide (25). Compound **9** (0.10 g, 0.082 mmol) was dissolved in dry DMF (1.5 mL) with stirring and 1-azido-2-iodoethane (**24**, 0.32 g, 0.16 mL, 1.641 mmol) was added dropwise. The mixture was stirred overnight at 35 °C under an inert atmosphere of argon. The reaction mixture changed the color to yellow and MeI (0.32 g, 0.08 mL, 1.230 mmol) was added dropwise. The mixture was then stirred at 35 °C for further 20 h. Completeness of the reaction was checked by TLC (MeOH/HOAc-1% solution of NH_4OAc in H_2O 10:1:9). The solvent was removed in vacuo and the residue codistilled with H_2O (3 × 2 mL). Then the solid

residue was dissolved in a minimum amount of H_2O/EtOH mixture (1.0 mL) and precipitated from acetone (10 mL). The precipitate was vacuum-filtered and dried in a desiccator over NaOH. Pure compound **25** (0.11 g) was obtained as white powder in 98% yield.

m.p. 200 °C (starts to decompose);

$[\alpha]^{25}_{D}$ +106.2° (H_2O);

IR (KBr): 3330 cm^{-1} ν(O-H), 2929 cm^{-1} ν(C-H), 2107 cm^{-1} ν(N_3), 1413 cm^{-1} δ(C-H), 1362 cm^{-1} δ(C-H), 1329 cm^{-1} δ(C-H), 1293 cm^{-1} δ(C-H), 1159 cm^{-1} ν(C-O), 1078 cm^{-1} ν(C-O), 1033 cm^{-1} ν(C-O).

^{1}H NMR (600 MHz, D_2O):): δ = 5.18 (d, J = 2.8 Hz, 1H, H-1^{I}), 5.13 – 5.03 (m, 6H, 6 × H-1), 4.43 (bt, J = 9.5 Hz, 1H, H-5^{I}), 4.12 – 3.57 (m, 48H, 7 × H-2, 7 × H-3, 6 × H-4, 6 × H-5, 14 × H-6, 2 × H-1’, 2 × H-2’, 2 × H-3’, 2 × H-4’), 3.47 (bt, J = 9.5 Hz, 1H, H-4), 3.38 – 3.36 (m, 6H, 2 × N-1-CH_3), 3.31 – 3.30 (m, 6H, 2 × N-2-CH_3) ppm.

^{13}C NMR (125 MHz, D_2O): δ = 104.10 - 103.64 (6 × C-1), 102.75 (C-1 ^{I}), 84.95 (C-4 ^{I}), 83.70 (C-4), 83.14 (C-4), 83.08 (C-4), 82.93 (C-4), 82.83 (C-4), 81.57 (C-4), 75.13 – 73.31 (7 × C-2, 7 × C-3, 6 × C-5), 69.54 (C-5^{I}), 66.53 (C-6^{I}), 65.02 (C-3’), 63.04 – 62.19 (6 × C-6), 60.15 (C-1’), 58.39 (C-2’), 54.82 (N-1-CH_3), 54.77 (N-1-CH_3), 54.17 (N-2-CH_3), 54.06 (N-2-CH_3), 46.39 (C-4’) ppm.

HRMS: for $C_{50}H_{89}O_{34}N_5$ calcd: *m/z* 1303.5383 (for $[M]^{2+}$ calcd 651.7689), found 651.7690 $[M]^{2+}$, Δ 0.07 ppm.

6^{I}-(*N’*-(2-Azidoethyl)-*N,N,N‘,N‘*-pentamethylpropane-1,3-diammonio)-6^{I}-deoxy-β-cyclodextrin diiodide (26). Compound **26** was prepared by the procedure described for the

synthesis of **25**. The reaction of starting compound **15** (0.50 g, 0.406 mmol) with 1-azido-2-iodoethane (**24**, 1.60 g, 0.80 mL, 8.114 mmol) and MeI (0.86 g, 0.38 mL, 6.086 mmol) afforded product **26** (0.604 g) as white powder in 94% yield.

m.p. 210 °C (starts to decompose);

$[\alpha]^{25}_D$ +97.7° (H_2O);

IR (KBr): 3281 cm^{-1} ν(O-H), 2929 cm^{-1} ν(C-H), 2110 cm^{-1} ν(N_3), 1458 cm^{-1} δ(C-H), 1410 cm^{-1} δ(C-H), 1387 cm^{-1} δ(C-H), 1368 cm^{-1} δ(C-H), 1153 cm^{-1} ν(C-O), 1077 cm^{-1} ν(C-O), 1028 cm^{-1} ν(C-O).

^{1}H NMR (600 MHz, D_2O):): δ = 5.15 – 5.03 (m, 7H, 7 × H-1), 4.33 (bt, J = 9.8 Hz, 1H, H-5^I), 4.07 – 3.47 (m, 49H, 7 × H-2, 7 × H-3, 7 × H-4, 6 × H-5, 14 × H-6, 2 × H-1’, 2 × H-3’, 2 × H-4’, 2 × H-5’), 3.29 (s, 3H, N-1-CH_3), 3.27 (s, 3H, N-1-CH_3), 3.23 (s, 6H, 2 × N-2-CH_3), 2.42 (m, 2H, 2 × H-2’) ppm.

^{13}C NMR (125 MHz, D_2O): δ = 104.25 (C-1), 103.95 (C-1), 103.95 (C-1), 103.85 (C-1), 103.82 (C-1), 103.68 (C-1), 102.84 (C-1 ^{I}), 85.13 (C-4 ^{I}), 83.74 (C-4), 83.26 (C-4), 83.23 (C-4), 82.95 (C-4), 82.79 (C-4), 81.61 (C-4), 75.20 – 73.43 (7 × C-2 , 7 × C-3, 6 × C-5), 69.47(C-5^I), 66.93 (C-6^I), 64.57 (C-1’), 64.43 (C-4’), 62.96– 62.22 (C-3’, 6 × C-6), 53.95 (N-1-CH_3), 53.78 (N-1-CH_3), 53.57 (N-2-CH_3), 53.52 (N-2-CH_3), 46.46 (C-5’), 18.91 (C-2’) ppm.

.

HRMS: for $C_{51}H_{91}O_{34}N_5$ calcd: *m/z* 1317.5540 (for $[M]^{2+}$ calcd 658,7767), found 658.7767 $[M]^{2+}$, Δ -0.03 ppm.

6^I-((2-((2-Aminoethyl)amino)ethyl)amino)-6^I-deoxy-β-cyclodextrin (27). Compound was prepared according to the published procedure [9] which was modified. Dry tosylate **1** (0.30

g, 0.233 mmol) was added portionwise to diethylenetriamine (3.60 g, 34.938 mmol) over 15 min to let totally dissolve. Then the temperature was raised to 60 °C and the reaction was stirred under an inert atmosphere of Ar for 18 h. The reaction was worked up by subsequent evaporation of unreacted amine under reduced pressure and by precipitating the solid residue from acetone (30 mL). The precipitate was filtrated in vacuo, superficially dried and introduced to 20 mL column packed with strong cation exchanger in H^+ form. The column was eluted first with H_2O (100 mL) to remove TsOH, then with 1% NH_4OH (50 mL) to remove byproducts and then with 3% NH_4OH (100 mL) to elute the product. The eluate was evaporated in vacuo, dissolved in H_2O (2 mL), added dropwise to EtOH (30 mL) with stirring and refluxed overnight. The mixture was evaporated and precipitated from acetone (30 mL). The precipitate was dried in vacuo to obtain pure **27** (0.20 g) as white powder in 70% yield.

m.p. 220 °C (starts to decompose);

$[\alpha]^{25}_{D}$ +130.9° (H_2O);

IR (KBr): 3318 cm^{-1} ν(O-H), 2926 cm^{-1} ν(C-H), 2836 cm^{-1} ν(C-H), 1455 cm^{-1} δ(C-H), 1419 cm^{-1} δ(C-H), 1365 cm^{-1} δ(C-H), 1293 cm^{-1} δ(C-H), 1156 cm^{-1} ν(C-O), 1081 cm^{-1} ν(C-O), 1036 cm^{-1} ν(C-O).

^{1}H NMR (600 MHz, D_2O):): δ = 5.07 – 5.05 (m, 7H, 7 × H-1), 3.95 – 3.82 (m, 26H, 7 × H-3, 7 × H-5, 12 × H-6), 3.65 – 3.55 (m, 13H, 7 × H-2, 6 × H-4), 3.45 (bt, J=9.2 Hz, 1H, H-4^I), 3.07 (m, 1H, H-6a^I), 2.89 – 2.72 (m, 9H, H-6b^I, 2 × H-1', 2 × H-2', 2 × H-3', 2 × H-4') ppm.

^{13}C NMR (125 MHz, D_2O): δ = 103.84 – 103.59 (7 × C-1), 85.48 (C-4^I), 83.07 – 82.94 (6 × C-4), 74.97 – 74.82 (7 × C-3), 73.91 – 73.77 (7 × C-2, 6 × C-5), 72.13 (C-5^I), 62.11 – 62.02 (6 × C-6), 51.07 (C-6^I), 50.43 (C-3'), 49.47 (C-1'), 49.23 (C-2'), 40.92 (C-4') ppm.

HRMS: for $C_{46}H_{82}O_{34}N_3$ calcd: *m/z* 1220.4774, found 1220.4774 $[M]^+$, Δ 0.17 ppm.

6^{I}-((3-((3-Aminopropyl)amino)propyl)amino)-6^{I}-deoxy-β-cyclodextrin (28). Compound **28** was prepared by the procedure described for the synthesis of **27**. The reaction of starting compound **1** (0.50 g, 0.388 mmol) with bis(3-aminopropyl)amine (10.18 g, 10.80 mL, 77.616 mmol) gave the product **28** (0.36 g) as white powder in 74% yield.

m.p. 220 °C (starts to decompose);

$[\alpha]^{25}_{D}$ +119.5° (H_2O);

IR (KBr): 3312 cm^{-1} ν(O-H), 2932 cm^{-1} ν(C-H), 1458 cm^{-1} δ(C-H), 1419 cm^{-1} δ(C-H), 1371 cm^{-1} δ(C-H), 1326 cm^{-1} δ(C-H), 1156 cm^{-1} ν(C-O), 1087 cm^{-1} ν(C-O), 1039 cm^{-1} ν(C-O).

^{1}H NMR (600 MHz, D_2O):): δ = 5.06 – 5.04 (m, 7H, 7 × H-1), 3.95 – 3.80 (m, 26H, 7 × H-3, 7× H-5, 12 × H-6), 3.64 – 3.54 (m, 13H,7 × H-2, 6 × H-4), 3.41 (t, J=9.4 Hz, 1H, H-4^{I}), 3.05 – 3.03 (m, 1H, H-$6a^{I}$), 2.79 – 2.58 (m, 9H, H-$6b^{I}$, 2 × H-1’, 2 × H-3’, 2× H-4’, 2 × H-6’), 1.71 – 1.65 (m, 4H, 2 × H-2’, 2 × H-5’) ppm.

^{13}C NMR (125 MHz, D_2O): δ = 103.50 -103.45 (6 × C-1), 103.11 (C-1^{I}), 85.24 C-4^{I}), 82.75 – 82.72 (5 × C-4), 82.39 (C-4), 74.70 – 73.48 (7 × C-2, 7 × C-3, 6 × C-5), 71.78 (C-5^{I}), 61.82 - 61.67 (6 × C-6), 50.83 (C-6^{I}), 48.20* (C-3’), 48.07* (C-4’), 47.56 (C-1’), 39.93 (C-6’), 31.37 (C-5’), 29.46 (C-2’) ppm. (signals * could be interchanged)

HRMS: for $C_{46}H_{85}O_{34}N_3$ calcd: *m/z* 1247.5014(for $[M+H]^+$ calcd 1248.5063), found 1248.5066 $[M+H]^+$, Δ 0.25 ppm.

6^{I}-((3-((3-Aminopropyl)amino)propyl)amino)-6^{I}-deoxy-α-cyclodextrin (29). Compound **29** was prepared by the procedure described for the synthesis of **27**. The reaction of starting compound **2** (0.30 g, 0.266 mmol) with bis(3-aminopropyl)amine (6.99 g, 7.50 mL, 53.239 mmol) gave the product **29** (0.21 g) as white powder in 71% yield.

m.p. 220 °C (starts to decompose);

$[\alpha]^{25}_{D}$ +37.9° (H_2O);

IR (KBr): 3318 cm^{-1} ν(O-H), 2923 cm^{-1} ν(C-H), 1413 cm^{-1} δ(C-H), 1359 cm^{-1} δ(C-H), 1329 cm^{-1} δ(C-H), 1153 cm^{-1} ν(C-O), 1084 cm^{-1} ν(C-O), 1033 cm^{-1} ν(C-O).

^{1}H NMR (600 MHz, D_2O):): δ = 5.06 – 5.04 (m, 6H, 6 × H-1), 4.00 – 3.85 (m, 22H, 6 × H-3, 6 × H-5, 10 × H-6), 3.64 – 3.56 (m, 11H, 6 × H-2, 5 × H-4) , 3.43 (bt, J=8.9 Hz, 1H, H-4^{I}), 3.11 – 3.08 (m, 1H, H-$6a^{I}$), 2.90 (bt, J=7.1 Hz, 1H, H-4'), 2.82 – 2.60 (m, 8H, 1 × H-$6b^{I}$, 2 × H-1', 2 × H-3', H-4', 2 × H-6'), 1.77 – 1.69 (m, 4H, 2 × H-2', 2 × H-5') ppm.

^{13}C NMR (125 MHz, D_2O): δ = 103.35 – 103.13 (6 × C-1), 85.72 (C-4^{I}), 83.16 – 80.07 (4 × C-4), 75.24 – 73.57 (6 × C-2, 6 × C-3, 5 × C-5), 72.78 (C-5^{I}), 62.38 – 62.28 (5 × C-6), 51.56 (C-6^{I}), 48.78 (C-1',*), 48.27 (C-3',*), 47.75 (C-4',*), 40.04 (C-6'), 30.56 (C-5'), 29.53 (C-2') ppm. (signals * could be interchanged)

HRMS: for $C_{42}H_{75}O_{29}N_3$ calcd: *m/z* 1085.4486 (for $[M+H]^+$ calcd 1086.4560), found 1086.4547 $[M+H]^+$, Δ -1.09 ppm.

6^{I}-((3-((3-aminopropyl)amino)propyl)amino)-6^{I}-deoxy-γ-cyclodextrin triacetate (30). Compound **30** was prepared by the procedure described for the synthesis of **27**. The reaction of starting compound **3** (0.40 g, 0.276 mmol) with bis(3-aminopropyl)amine (5.43 g, 5.80 mL, 41.368 mmol) gave product **30** (0.29 g) as white powder in 74% yield. The product was purified on silica gel column with isocratic elution (MeOH/HOAc-1% solution of NH_4OAc in H_2O 10:0,1:9) and was obtained in the form of triacetate salt.

m.p. 210 °C (starts to decompose);

$[\alpha]^{25}_{D}$ +135.8° (H_2O);

IR (KBr): 3267 cm^{-1} ν(O-H), 2929 cm^{-1} ν(C-H), 2836 cm^{-1} ν(C-H), 1556 cm^{-1} ν (C=O acetate) 1407 cm^{-1} δ(C-H), 1374 cm^{-1} δ(C-H), 1162 cm^{-1} ν(C-O), 1081 cm^{-1} ν(C-O), 1036 cm^{-1} ν(C-O).

^{1}H NMR (600 MHz, D_2O):): δ = 5.19 (d, J=2.9 Hz, 1H, H-1^I), 5.13 – 5.11 (m, 7H, 7 × H-1), 4.09 (t, J=9.2 Hz, 1H, H-5^I), 3.98 – 3.51 (m, 29H, 8 × H-3, 7 × H-5, 14 × H-6), 3.69 – 3.56 (m, 16H, 8 × H-2, 8 × H-4), 3.45 (d, J=12.6 Hz, 1H, H-$6a^I$), 3.24 (t, J=9.2 Hz, 1H, H-$6b^I$), 3.15 – 3.06 (m, 8H, 2 × H-1’, 2 × H-3’, 2 × H-4’, 2 × H-6’), 2.12 – 2.07 (m, 4H, 2 × H-2’, 2 × H-5’), 1.92 (s, 9H, 3 × CH_3COOH) ppm.

^{13}C NMR (125 MHz, D_2O): δ = 183.46 (CH_3**C**OOH), 103.62 -103.46 (7 × C-1), 102.79 (C-1^I), 84.63 (C-4^I), 82.51 (C-4), 82.47 (C-4), 82.40 (C-4), 82.35 (C-4), 82.33 (C-4), 82.30 (C-4), 81.22 (C-4), 74.88 – 73.69 (8 × C-2, 8 × C-3, 7 × C-5), 70.10 (C-5^I), 62.53 – 62.15 (7 × C-6), 50.51 (C-6^I), 47.47 (C-1’,*), 47.01 (C-3’,*), 46.71 (C-4’,*), 38.59 (C-6’), 25.94 (C-2’, **), 25.38 (C-5’,**), 25.04 (**C**H_3COOH) ppm. (signals *,** could be interchanged)

HRMS: for $C_{54}H_{95}O_{39}N_3$ calcd: *m/z* 1409.5543 (for $[M+H]^+$ calcd 1410.5616), found 1410.5624 $[M+H]^+$, Δ 0.58 ppm.

6^I-(*N*,*N*,*N*‘,*N*‘-Tetramethyl-*N*‘-(3-(trimethylammonio)propyl)propane-1,3-diammonium)-6^I-deoxy-β-cyclodextrin tribicarbonate (31). Dry triamine derivative **28** (0.080 g, 0.064 mmol) was dissolved in dry DMF (4 mL) and heated at 60 °C for 30 min to prevent self-inclusion. After cooling down to rt, 2,4,6-collidine (0.411 g, 0.45 mL, 3.848 mmol) and MeI (0.910 g, 0.40 mL, 6.410 mmol) were added. The reaction mixture was stirred for 20 h under argon at 30 °C. The reaction mixture changed the color to yellow upon completion. Work-up consisted of evaporating the solvent and unreacted reagents in vacuo, precipitating the yellow solid residue from acetone (40 mL). The precipitate was centrifuged (5000 RPM, 5 min) and dried. The product was purified on column packed with weak anion

exchanger (Amberlite CG 50) in NH_4^+ form, which was firstly washed with H_2O and then with aqueous ammonia (3–10%) and then with aqueous NH_4HCO_3 (3–10%). Pure **31** tribicarbonate (0.058 g) was obtained as white powder in 67% yield.

m.p. 200 °C (starts to decompose);

$[\alpha]^{25}_D$ +103.5° (H_2O);

IR (KBr): 3285 cm^{-1} ν(O-H), 2926 cm^{-1} ν(C-H), 2836 cm^{-1} ν(C-H), 1565 cm^{-1} ν (C=O acetate), 1476 cm^{-1} δ(C-H), 1404 cm^{-1} δ(C-H), 1338 cm^{-1} δ(C-H), 1156 cm^{-1} ν(C-O), 1078 cm^{-1} ν(C-O), 1036 cm^{-1} ν(C-O).

^1H NMR (600 MHz, D_2O):): δ = 5.13 (d, J=3.8 Hz, 1H, H-1), 5.12 (d, J=3.1 Hz, 1H, H-1^I), 5.09 (d, J=3.1 Hz, 1H, H-1), 5.07 (d, J=3.5 Hz, 1H, H-1), 5.04 (d, J=3.3 Hz, 1H, H-1), 5.02 (d, J= 3.8 Hz, 2H, 2 × H-1), 5.01 (d, J=3.3 Hz, 1H, H-1), 4.52 (t, J=9.5 Hz, 1H, H-5^I), 4.08 (t, J=9.2 Hz, 1H, H-3^I), 4.02 – 3.42 (m, 48 H, 7 × H-2. 6 × H-3, 7 × H-4, 6 × H-5, 14 × H-6, 2 × H-1’, 2 × H-3’, 2 × H-4’, 2 × H-6’), 3.27 (s, 3H, N-CH_3), 3.26 (s, 3H, N-CH_3), 3.21 (s, 6H, 2 × N-CH_3), 3.19 (s, 9H, 3 × N-CH_3), 2.46 – 2.36 (m, 4H, 2 × H-2’, 2 × H-5’) ppm.

^{13}C NMR (125 MHz, D_2O): δ = 103.78 (C-1), 103.41 (2 × C-1), 103.34 (C-1), 103.19 (C-1), 103.15 (C-1), 102.08 (C-1^I), 84.63 (C-4^I), 83.28 (C-4), 82.61 (C-4), 82.59 (C-4), 82.47 (C-4), 82.33 (C-4), 80.85 (C-4), 74.78 – 73.05 (7 × C-2, 7 × C-3, 6 × C-5), 68.86 (C-5^I), 66.88 (C-6^I), 64.20 C-1’), 63.84 (C-6’), 62.86 (C-3’*), 62.65 (C-4’*, C-6), 62.17 (2 × C-6), 62.03 (C-6), 61.88 (2 × C-6), 54.87 (3 × N-CH_3), 53.73 (N-CH_3), 53.32 (N-CH_3), 52.24 (N-CH_3), 52.20 (N-CH_3), 18.77 (C-2’*), 18.62 (C-5’*) ppm.

HRMS: for $C_{55}H_{102}O_{34}N_3$ calcd: *m/z* 1348.6328 (for $[M]^{3+}$ calcd 449.5443), found 449.5443 $[M]^{3+}$, Δ 0.11 ppm.

6^I-(*N*,*N*,*N*‘,*N*‘-Tetramethyl-*N*‘-(3-(trimethylammonio)propyl)propane-1,3-diammonium)-6^I-deoxy-α-cyclodextrin triacetate (32). Compound **32** was prepared by the procedure described for the synthesis of **31**, which differs only in the purification step. The product was purified on silica gel column with eluent (MeOH/HOAc-1% solution of NH_4OAc in H_2O 10:0,1:9). Fractions containing pure **32** were merged, evaporated in vacuo and codistilled with H_2O (4 × 5 mL) to eliminate ammonium acetate. The reaction of starting compound **29** (0.10 g, 0.092 mmol) with 2,4,6-collidine (0.591 g, 0.64 mL, 5.524 mmol) and MeI (1.307 g, 0.57 mL, 9.207 mmol) gave the product **32** triacetate (0.068 g) as white powder in 54% yield.

m.p. 200 °C (starts to decompose);

$[\alpha]^{25}_D$ +101.2° (H_2O);

IR (KBr): IR (KBr): 3249 cm^{-1} ν(O-H), 2926 cm^{-1} ν(C-H), 2839 cm^{-1} ν(C-H), 1580 cm^{-1} ν (C=O acetate), 1488 cm^{-1} δ(C-H), 1404 cm^{-1} δ(C-H), 1341 cm^{-1} δ(C-H), 1156 cm^{-1} ν(C-O), 1084 cm^{-1} ν(C-O), 1039 cm^{-1} ν(C-O).

^1H NMR (600 MHz, D_2O):): δ = 5.18 (d, J=2.7 Hz, 1H, H-1), 5.11 – 5.03 (m, 5H, 5 × H-1), 4.49 (dd, J_1=9.4 Hz, J_2=9.4 Hz, 1H, H-5^I), 4.12 – 3.43 (m, 43H, 6 × H-2, 6 × H-3, 6 × H-4, 5 × H-5, 12 × H-6, 2 × H-1’, 2 × H-3’, 2 × H-4’, 2 × H-6’), 3.28 (s, 3H, N-1-CH_3), 3.25 (s, 3H, N-1-CH_3), 3.20 (s, 15H, 2 × N-2-CH_3, 3 × N-3-CH_3), 2.45 – 2.36 (m, 4H, 2 × H-2’, 2 × H-5’), 1.92 (s, 9H, 3 × CH_3COO^-) ppm.

^{13}C NMR (125 MHz, D_2O): δ = 183.29 (CH_3**C**OO^-), 103.69 (C-1), 103.41 (C-1), 103.27 (C-1), 103.22 (2 × C-1), 103.06 (C-1), 85.28 (C-4^I), 83.70 (C-4), 83.31 (C-4), 83.24 (C-4), 83.18 (C-4), 82.79 (C-4), 75.39 – 73.17 (6 × C-2, 6 × C-3, 5 × C-5), 69.94 (C-5^I), 66.91 – 62.42 (6 × C-6, C-1’, C-3’, C-4’, C-6’), 55.17 (3 × N-3-CH_3), 53.86 (N-1-CH_3), 53.56 (N-1-CH_3), 52.31 (N-2-CH_3), 52.18 (N-2-CH_3), 25.18 (**C**H_3COO^-), 18.98 (C-2’, *), 18.76 (C-4’,*) ppm. (signals * could be interchanged)

HRMS: for $C_{49}H_{92}O_{29}N_3$ calcd: *m/z* 1186.5800 (for $[M]^{3+}$ calcd 395.5267), found 395.5267 $[M]^{3+}$, Δ 0.07 ppm.

6^I-(*N,N,N‘,N‘*-Tetramethyl-*N‘*-(3-(trimethylammonio)propyl)propane-1,3-diammonium)-6^I-deoxy-γ-cyclodextrin triacetate (33). Compound **33** was prepared by the procedure described for the synthesis of **32**. The reaction of starting compound **30** (0.20 g, 0.071 mmol) with 2,4,6-collidine (0.455 g, 0.49 mL, 4.254 mmol) and MeI (1.01 g, 0.44 mL, 7.091 mmol) gave the product **33** acetate (0.045 g) as white powder in 42% yield.

m.p. 190 °C (starts to decompose);

$[\alpha]^{25}_D$ +93.1° (H_2O);

IR (KBr): 3150 cm^{-1} ν(O-H), 2929 cm^{-1} ν(C-H), 2896 cm^{-1} ν(C-H), 2839 cm^{-1} ν(C-H), 1556 cm^{-1} ν(C=O acetate), 1404 cm^{-1} δ(C-H), 1371 cm^{-1} δ(C-H), 1329 cm^{-1} δ(C-H), 1156 cm^{-1} ν(C-O), 1084 cm^{-1} ν(C-O), 1030 cm^{-1} ν(C-O).

^{1}H NMR (600 MHz, D_2O):): δ = 5.24 (bs, 1H, H-1^I), 5.18 - 5.10 (m, 7H, 7 × H-1), 4.58 (t, J=9.1 Hz, 1H, H-5^I), 4.09 (t, J=9.4 Hz, 1H, H-3^I), 4.01 – 3.44 (m, 54H, 8 × H-2, 7 × H-3, 8 × H-4, 7 × H-5, 16 × H-6, 2 × H-1’, 2 × H-3’, 2 × H-4’, 2 × H-6’), 3.26 – 3.21 (m, 21H, 2 × N-1-CH_3, 2 × N-2-CH_3, 3 × N-3-CH_3), 2.41 (bs, 4H, 2 × H-2’, 2 × H-5’), 1.92 (s, 9H, 3 × CH_3COO^-) ppm.

^{13}C NMR (125 MHz, D_2O): δ = 183.36 (CH_3**C**OO^-), 103.75 – 103.22 (7 × C-1), 101.57 (C-1^I), 84.42 (C-4^I), 82.82 (C-4), 82.38 (C-4), 82.31 (2 × C-4), 82.22 (C-4), 82.13 (C-4), 79.42 (C-4), 75.12 – 73.47 (8 × C-2, 7 × C-3, 7 × C-5), 68.71 (C-5^I), 66.92 (C-6^I), 64.74 – 62.21 (7 × C-6, C-1’, C-3’, C-4’, C-6’), 55.19 (3 × N-3-CH_3), 54.00 (N-1-CH_3), 53.22 (N-1-CH_3), 52.31 (N-2-CH_3), 52.22 (N-2-CH_3), 25.22 (**C**H_3COO^-), 19.00 (C-2’, *), 18.78 (C-4’,*) ppm. (signals * could be interchanged)

HRMS: for $C_{61}H_{112}O_{39}N_3$ calcd: *m/z* 1510.6857 (for $[M]^{3+}$ calcd 503.5619), found 503.5620 $[M]^{3+}$, Δ 0.07 ppm.

References

1. Zhong, N.; Byun, H.-S.; Bittman, R. *Tetrahedron Lett.* **1998**, *39*, 2919–2920.
2. Tang, W.; Ng, S.-C. *Nat Protoc.* **2008**, *3*, 691–697.
3. De Costa, B. R.; Radesca, L.; Di Paolo, L.; Bowen, W. D. *J. Med. Chem.* **1992**, *35*, 38–47.
4. Shawali, A. S. A. S.; Biechler, S. S.; Biechler, S. S. *J. Am. Chem. Soc.* **1967**, *89*, 3020–3026.
5. Nzeadibe, K.; Vigh, G. *Electrophoresis* **2007**, *28*, 2589–2605.
6. Choudary, B. M.; Chowdari, N. S.; Kantam, M. L. *Tetrahedron* **2000**, *56*, 7291–7298.
7. Demko, Z. P.; Sharpless, K. B. *Org. Lett.* **2001**, *3*, 4091–4094.
8. Khoukhi, M.; Vaultier, M.; Carrié, R. *Tetrahedron Lett.* **1986**, *27*, 1031–1034.
9. Tabushi, I.; Shimizu, N.; Sugimoto, T.; Shiozuka, M.; Yamamura, K. *J. Am. Chem. Soc.* **1977**, *99*, 7100–7102.

^{1}H and ^{13}C NMR spectra of prepared compounds

^{1}H NMR of compound **1** (600 MHz, DMSO-d_6)

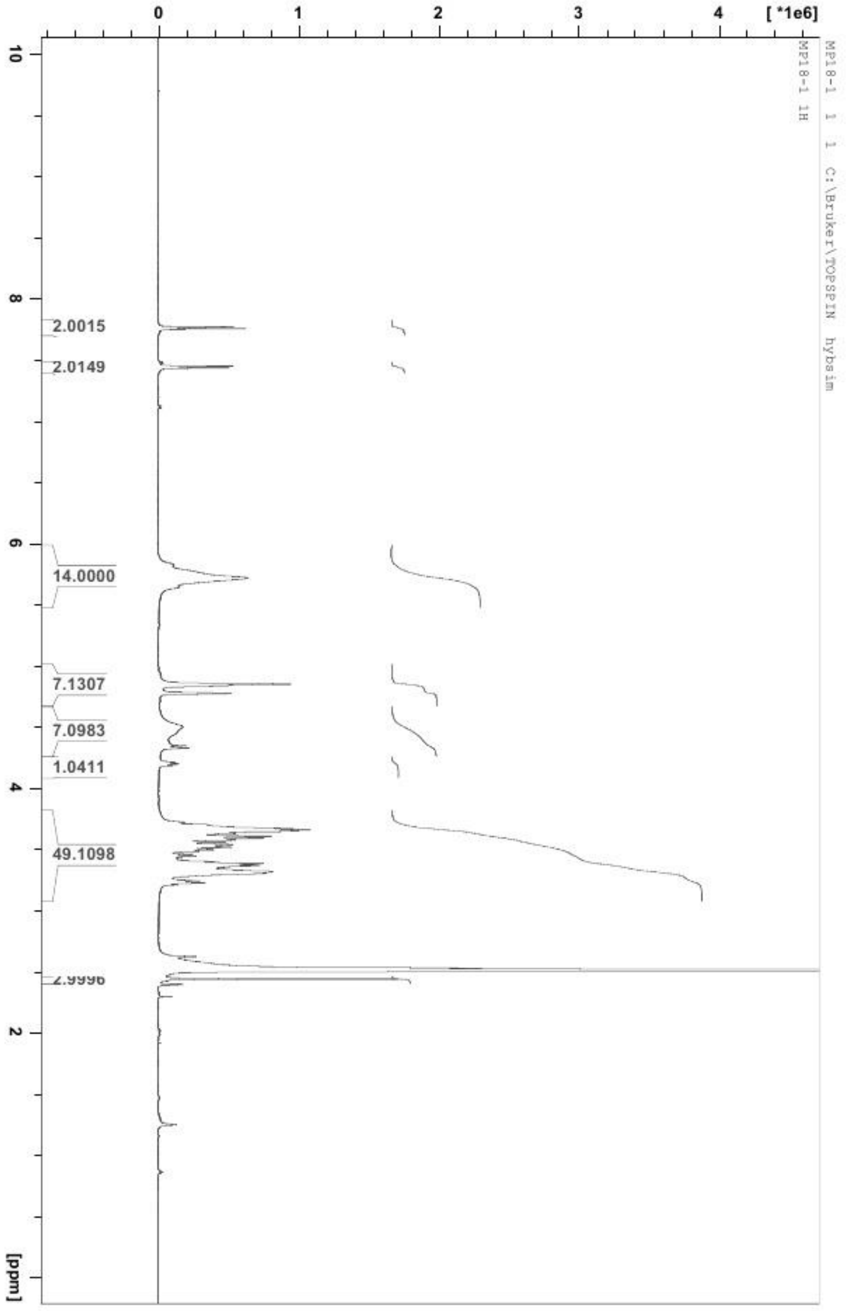

^{13}C NMR of compound **1** (150 MHz, DMSO-d_6)

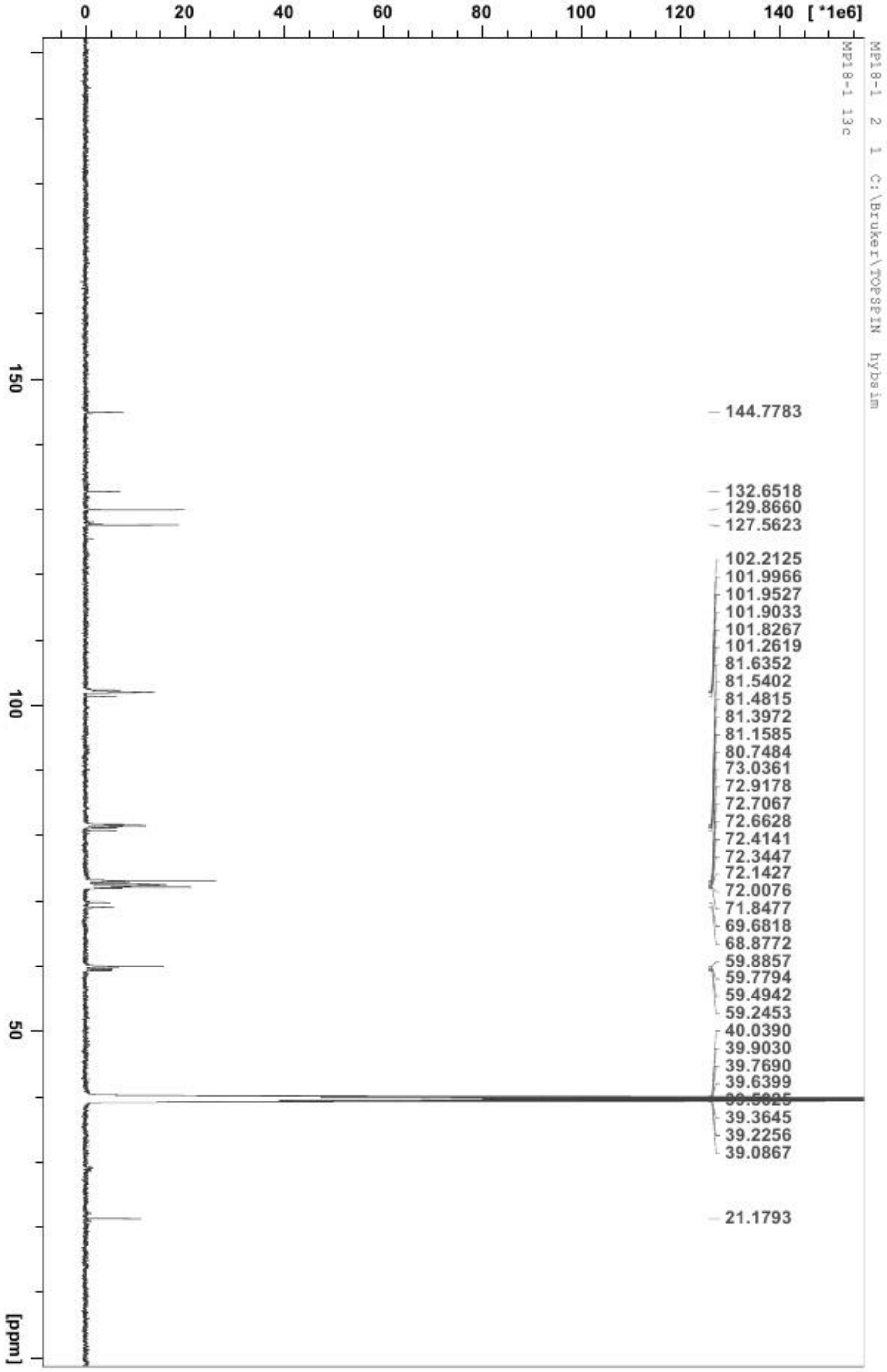

^{1}H NMR of compound **2** (600 MHz, DMSO-d_6)

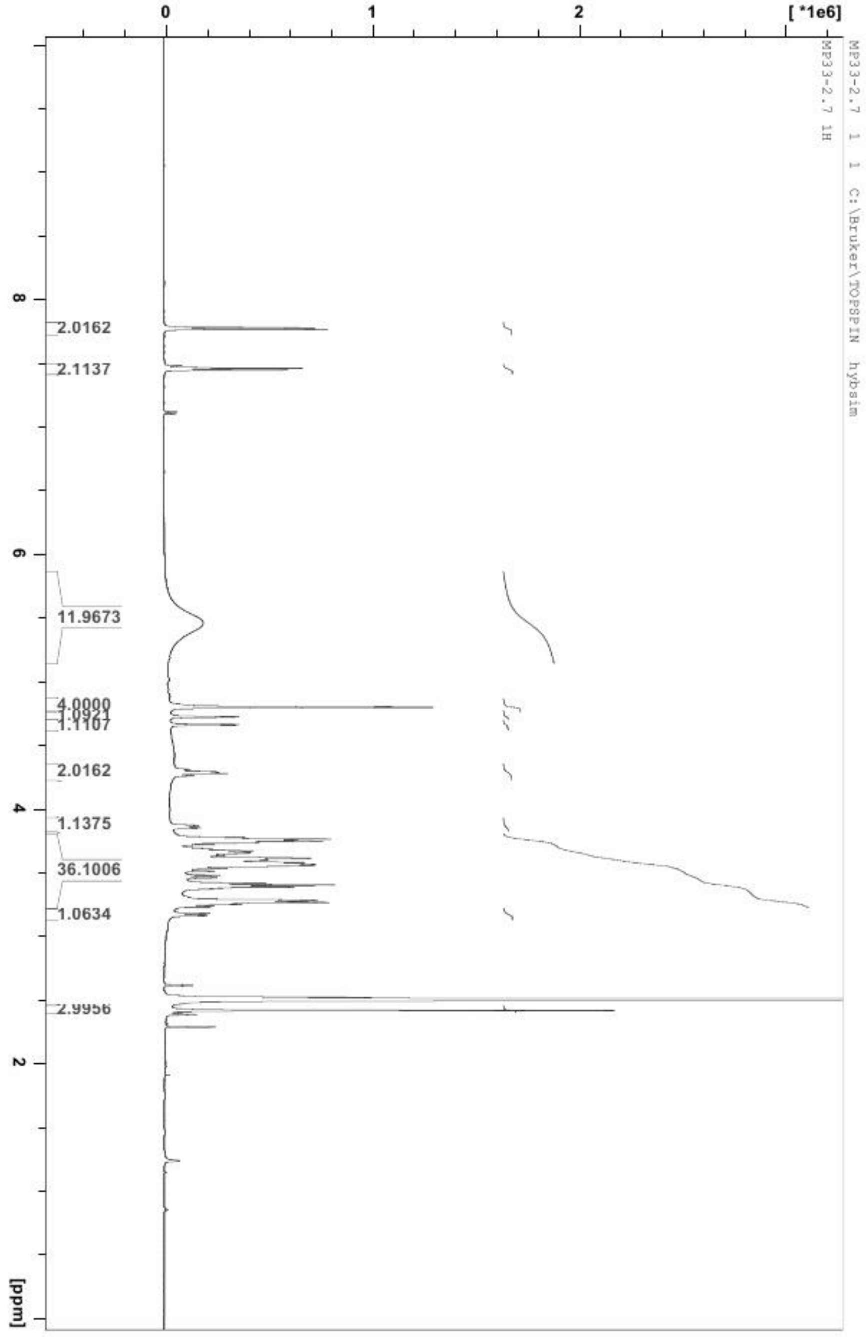

^{13}C NMR of compound **2** (150 MHz, DMSO-d_6)

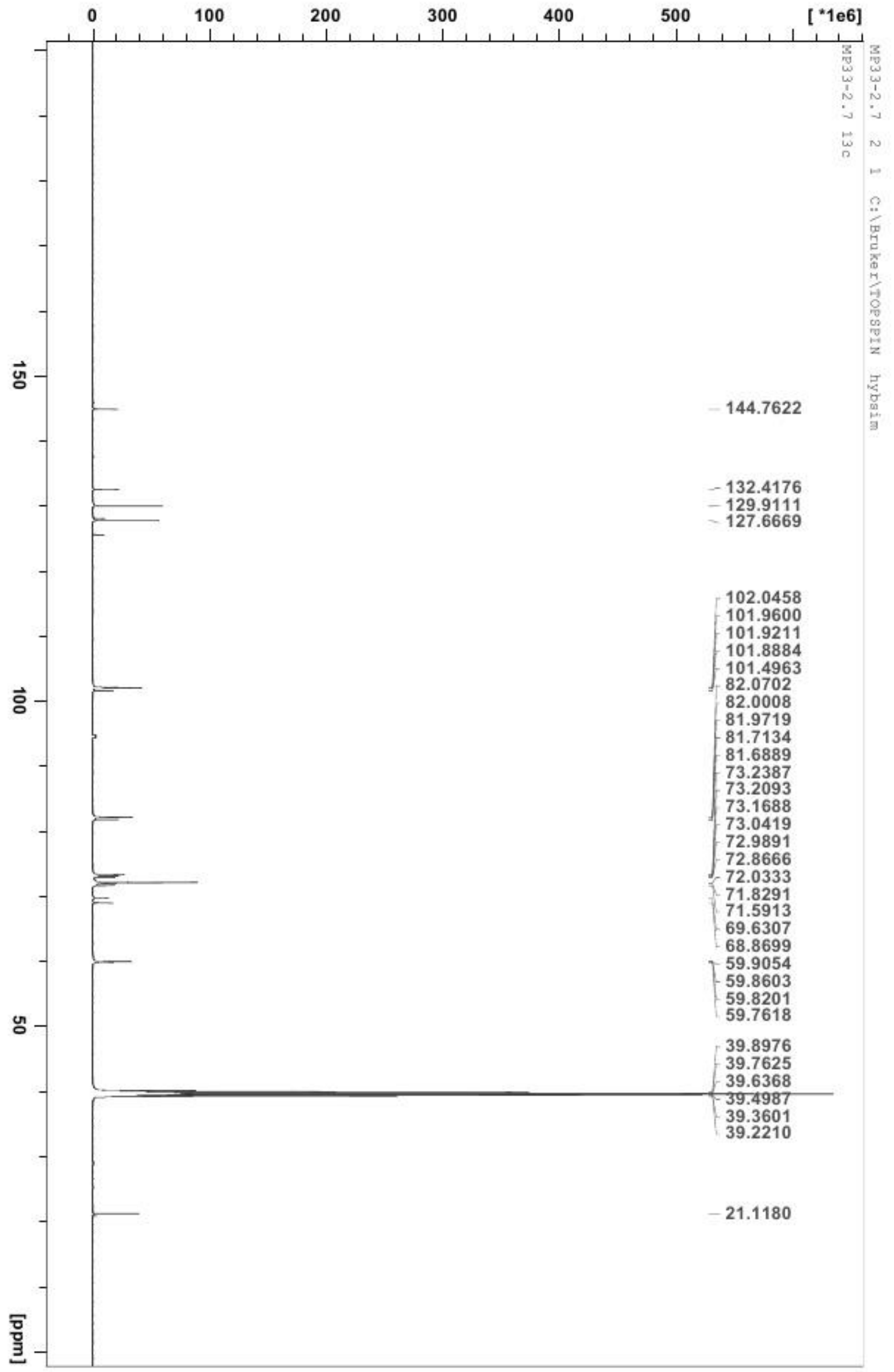

^{1}H NMR of compound **3** (600 MHz, DMSO-d_6)

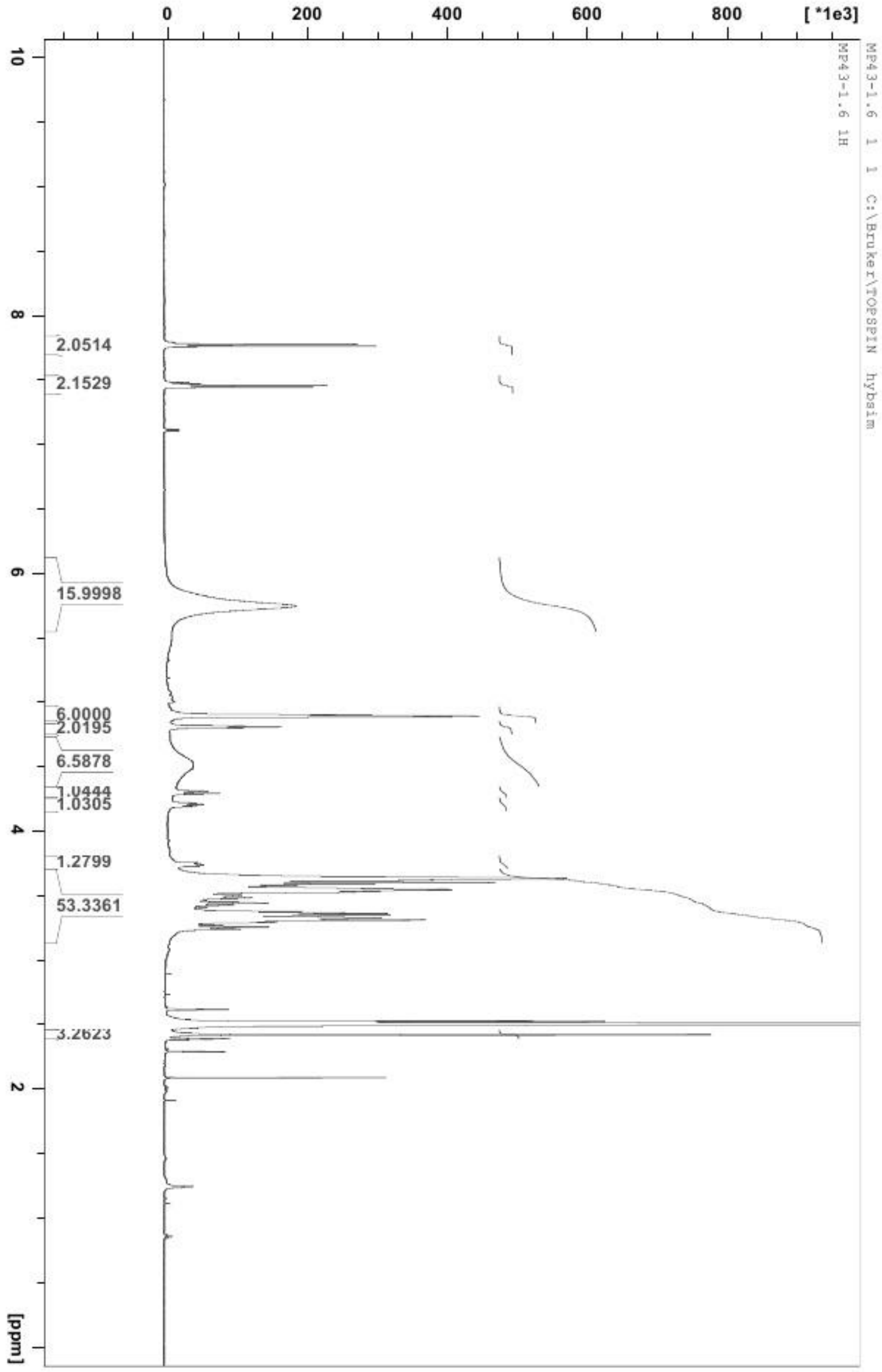

^{13}C NMR of compound **3** (150 MHz, DMSO-d_6)

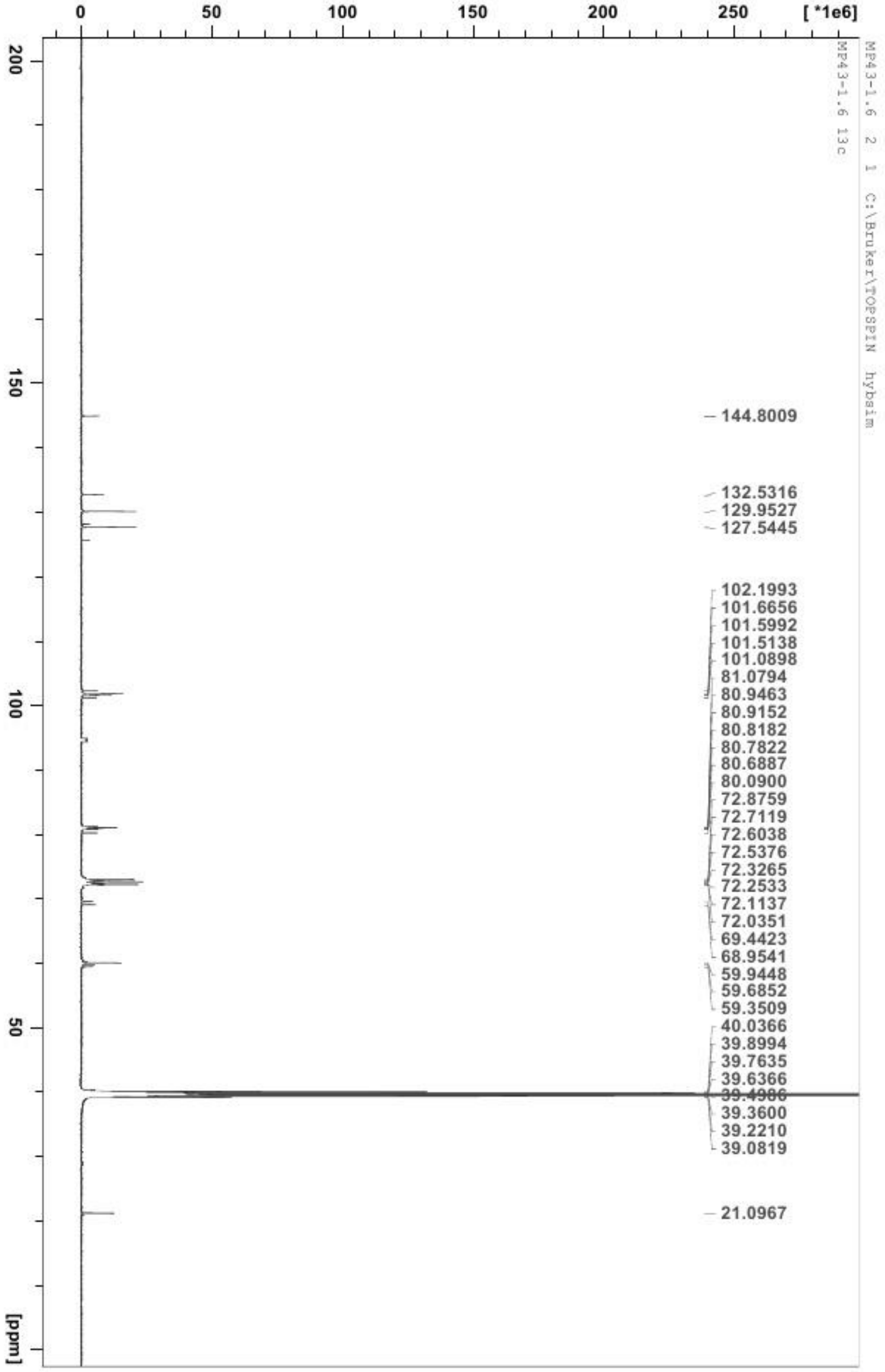

^{1}H NMR of compound **4** (600 MHz, D_2O)

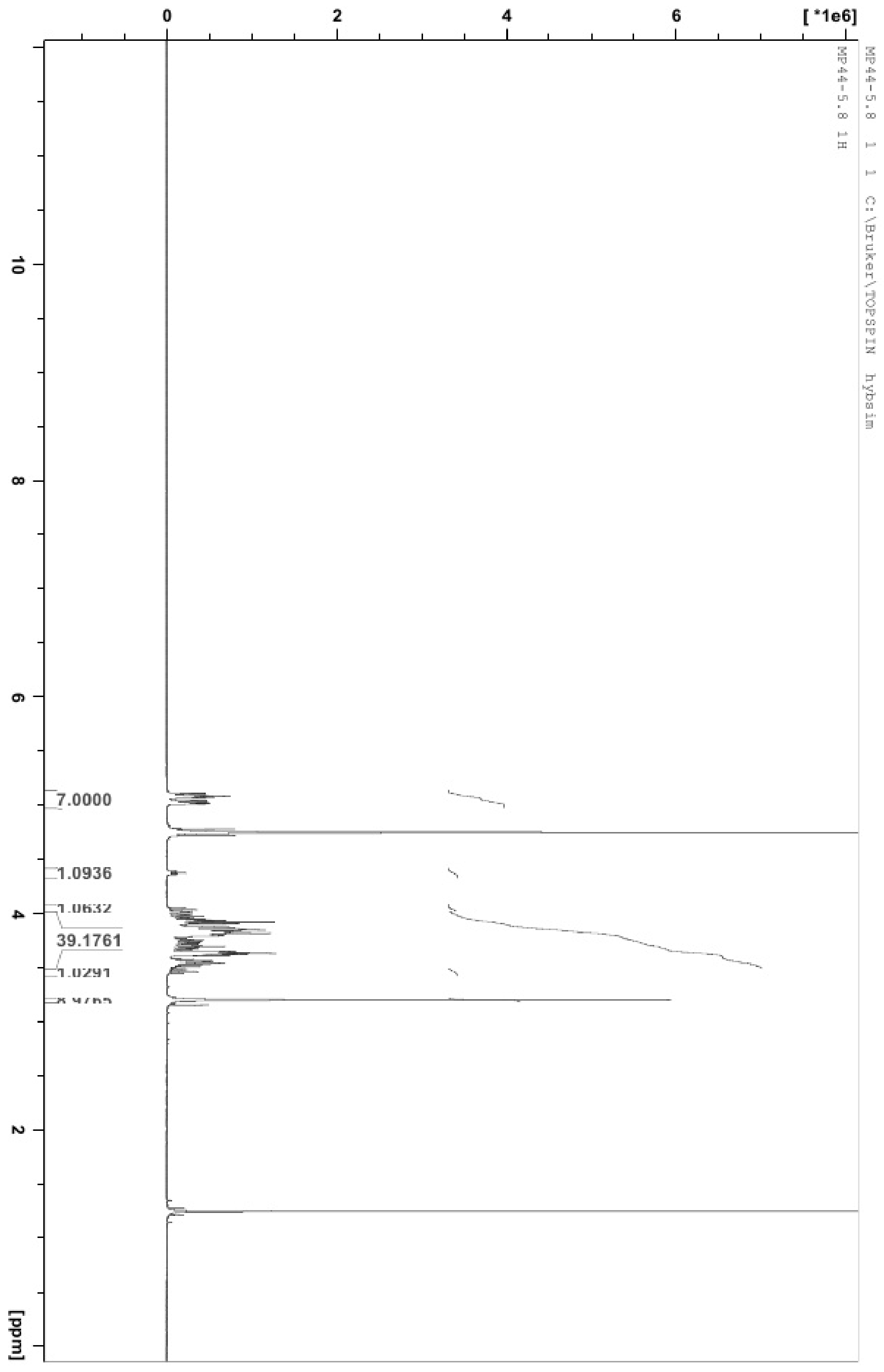

^{13}C NMR of compound **4** (150 MHz, D_2O)

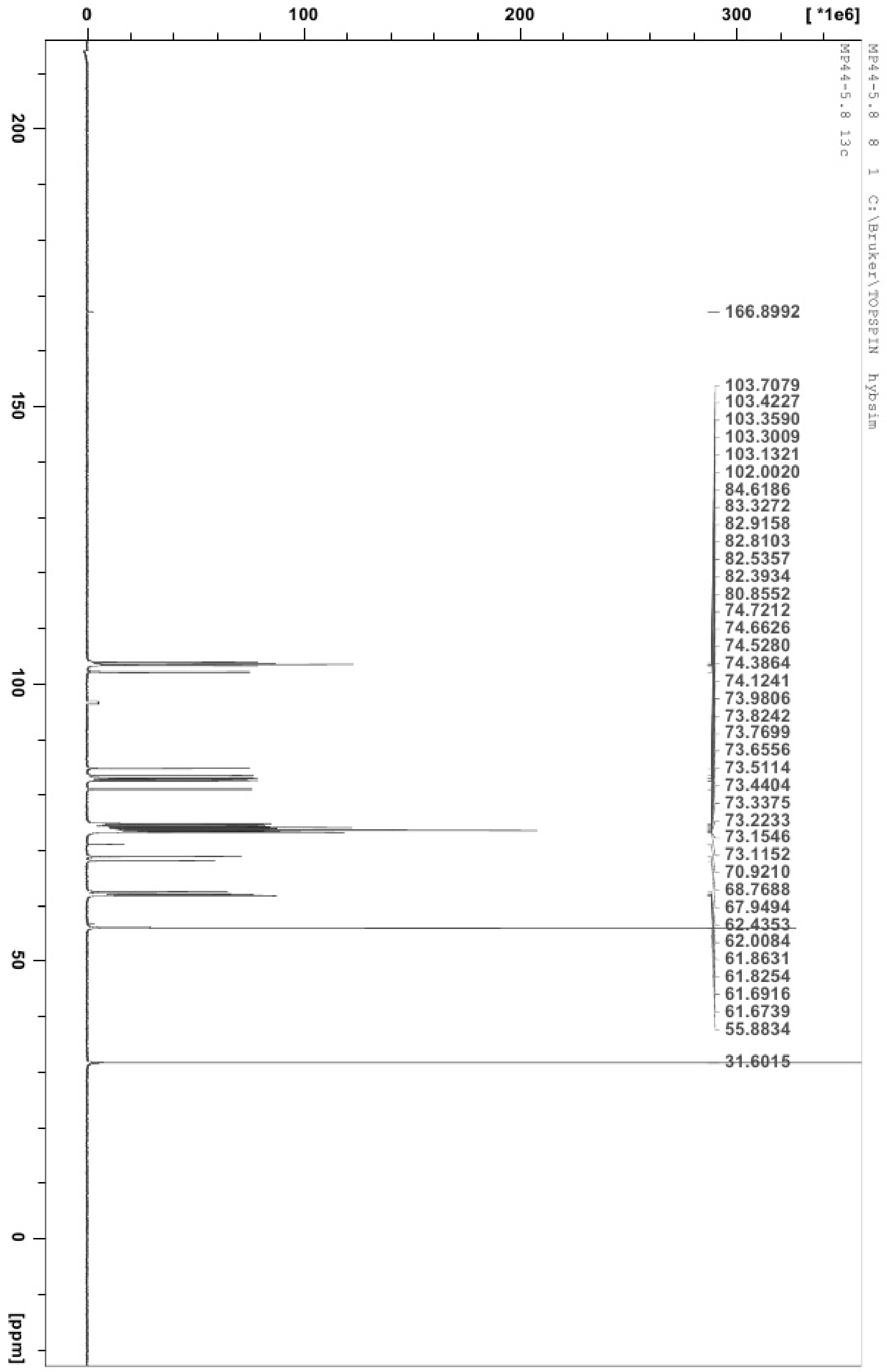

^{1}H NMR of compound **5** (600 MHz, D_2O)

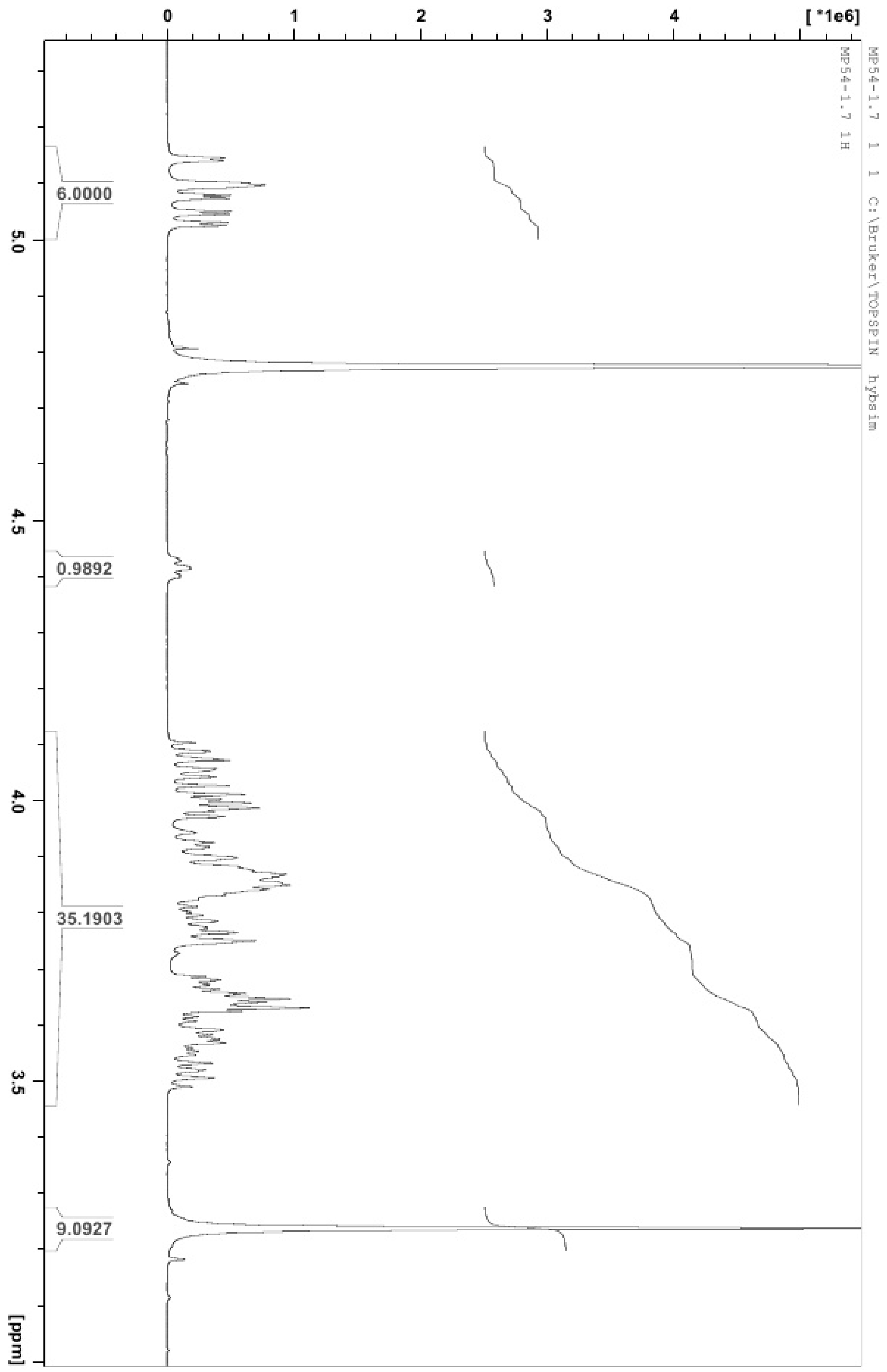

^{13}C NMR of compound **5** (150 MHz, D_2O)

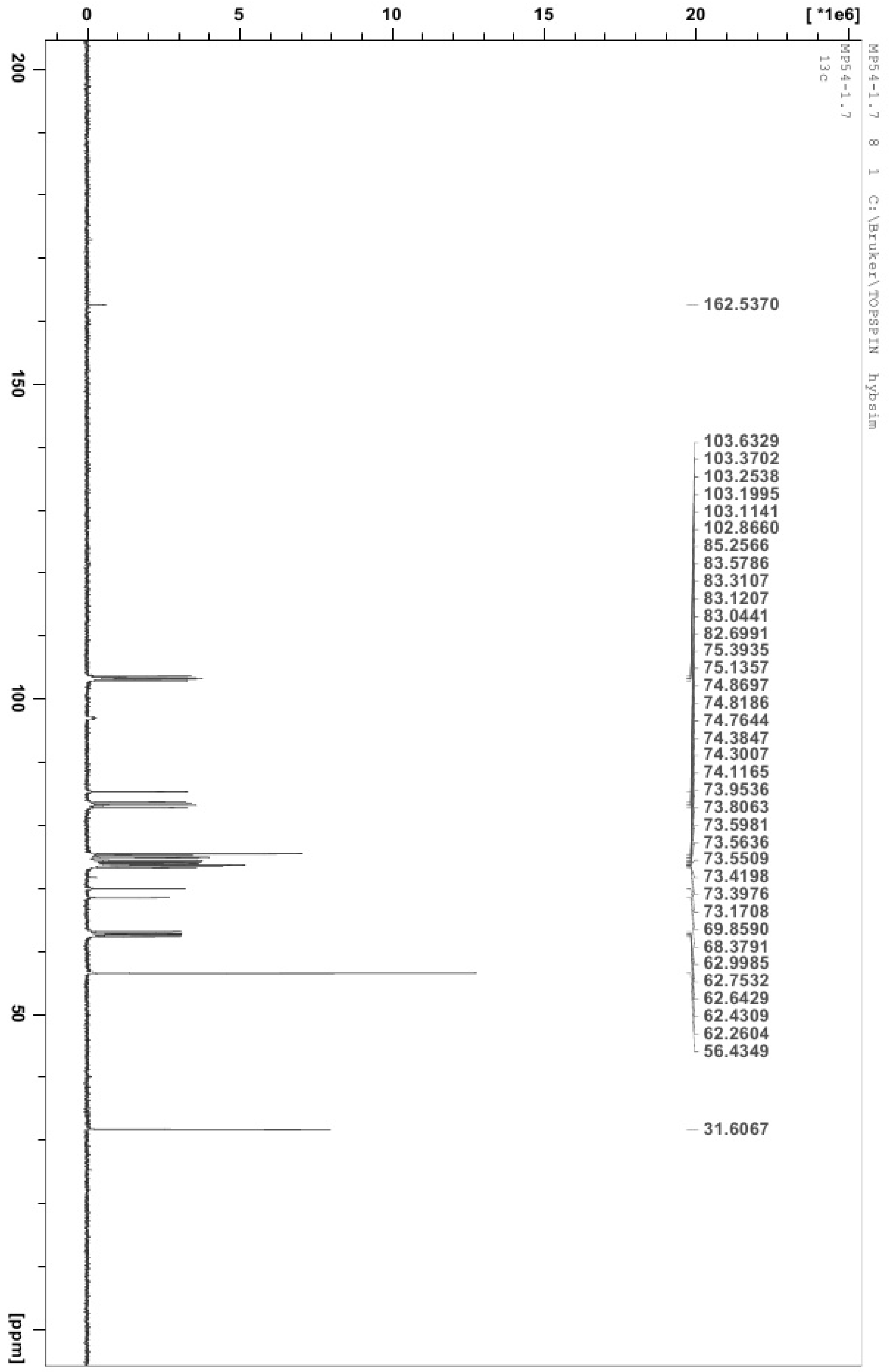

^{1}H NMR of compound **6** (600 MHz, D_2O)

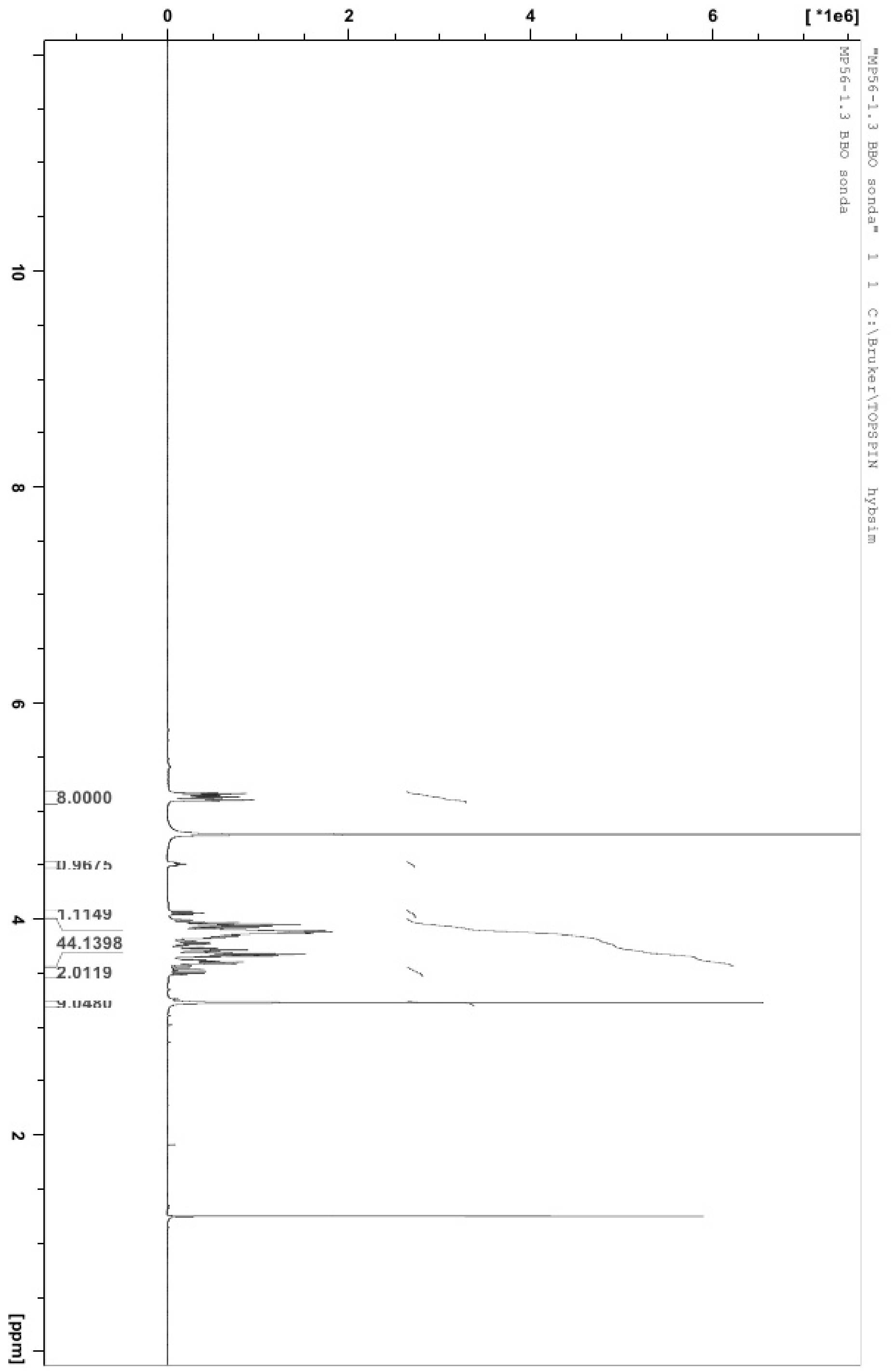

^{13}C NMR of compound **6** (150 MHz, D_2O)

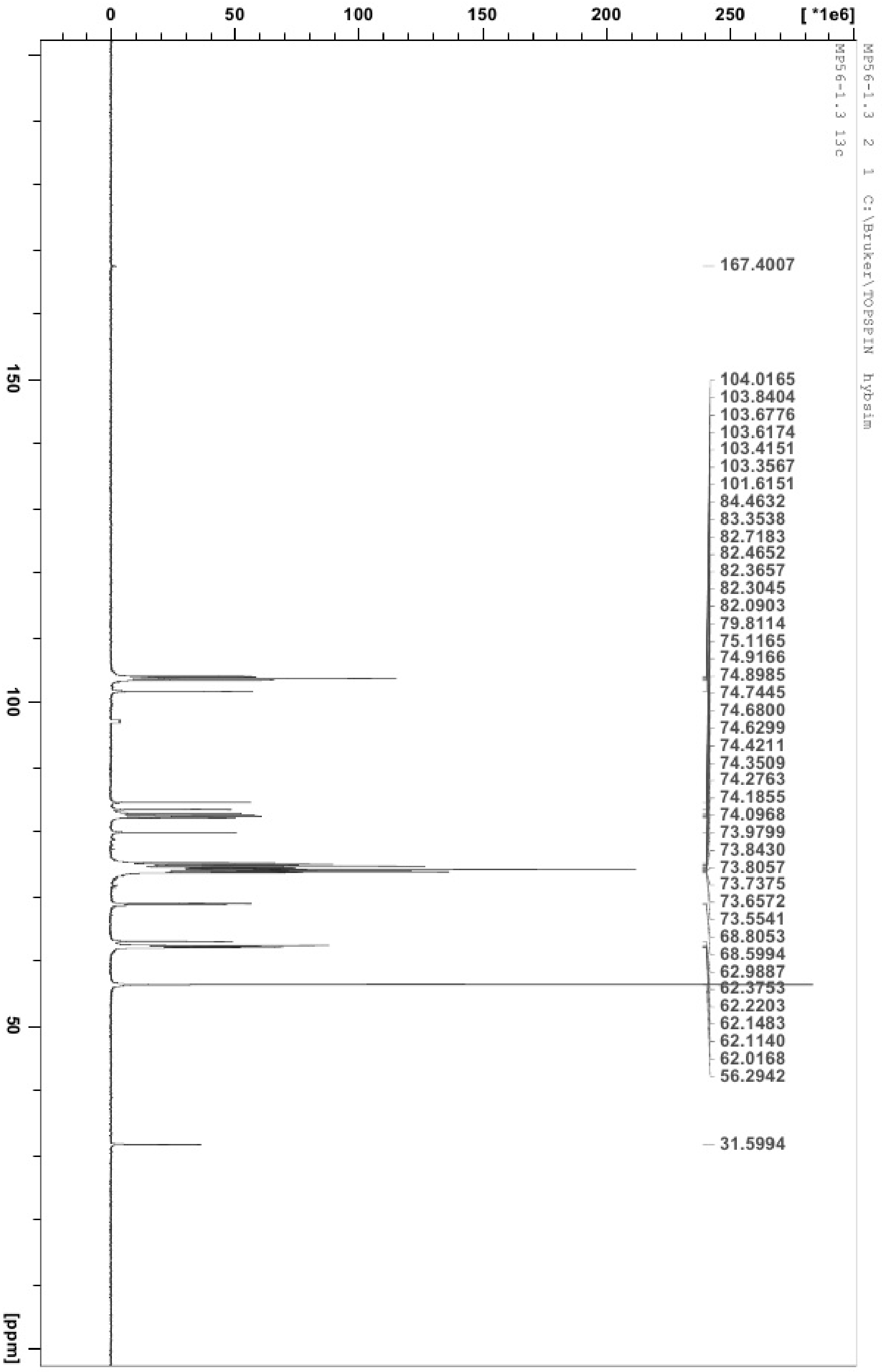

^{1}H NMR of compound **9** (600 MHz, DMSO-d_6)

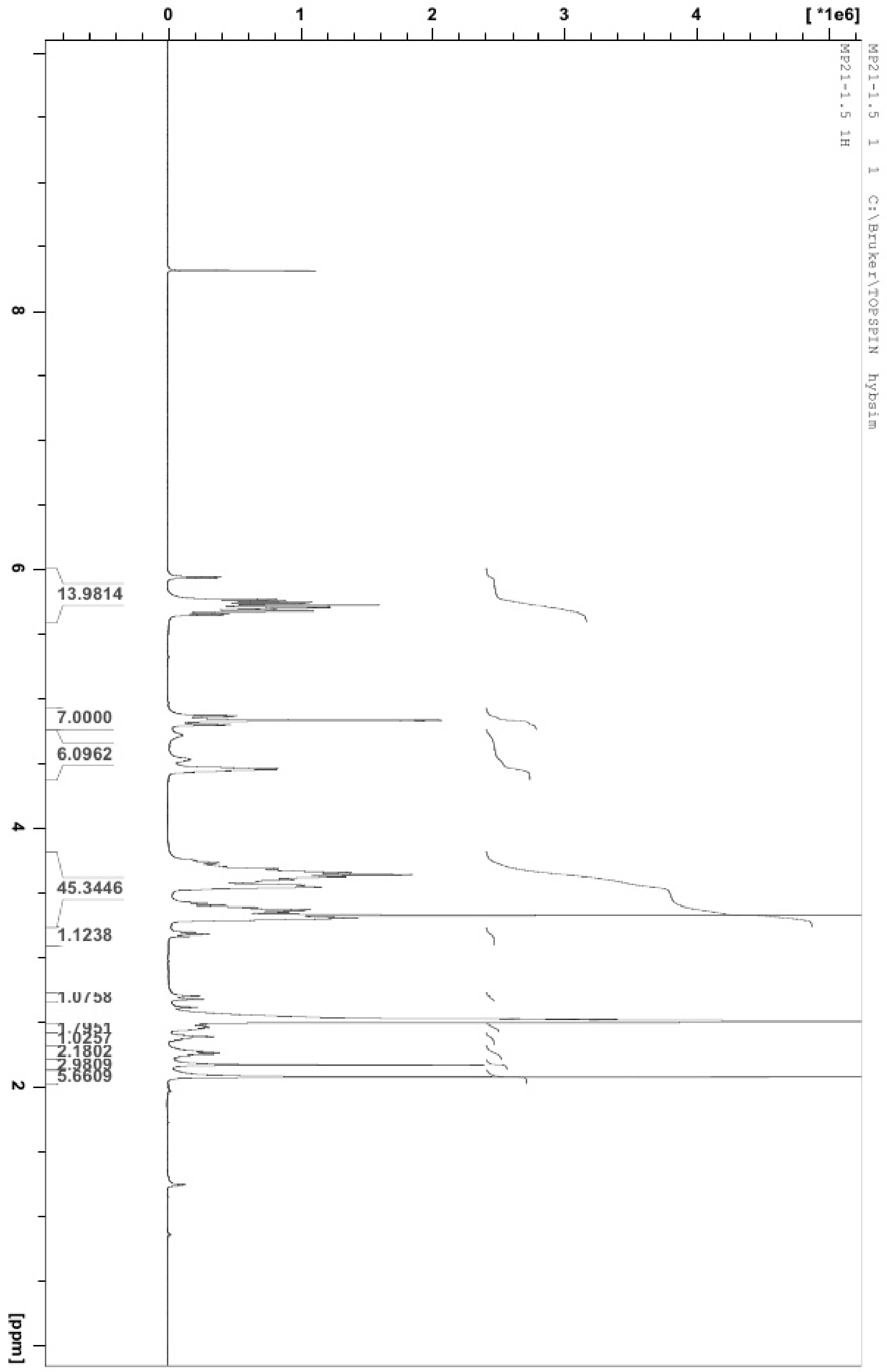

^{13}C NMR of compound **9** (150 MHz, DMSO-d_6)

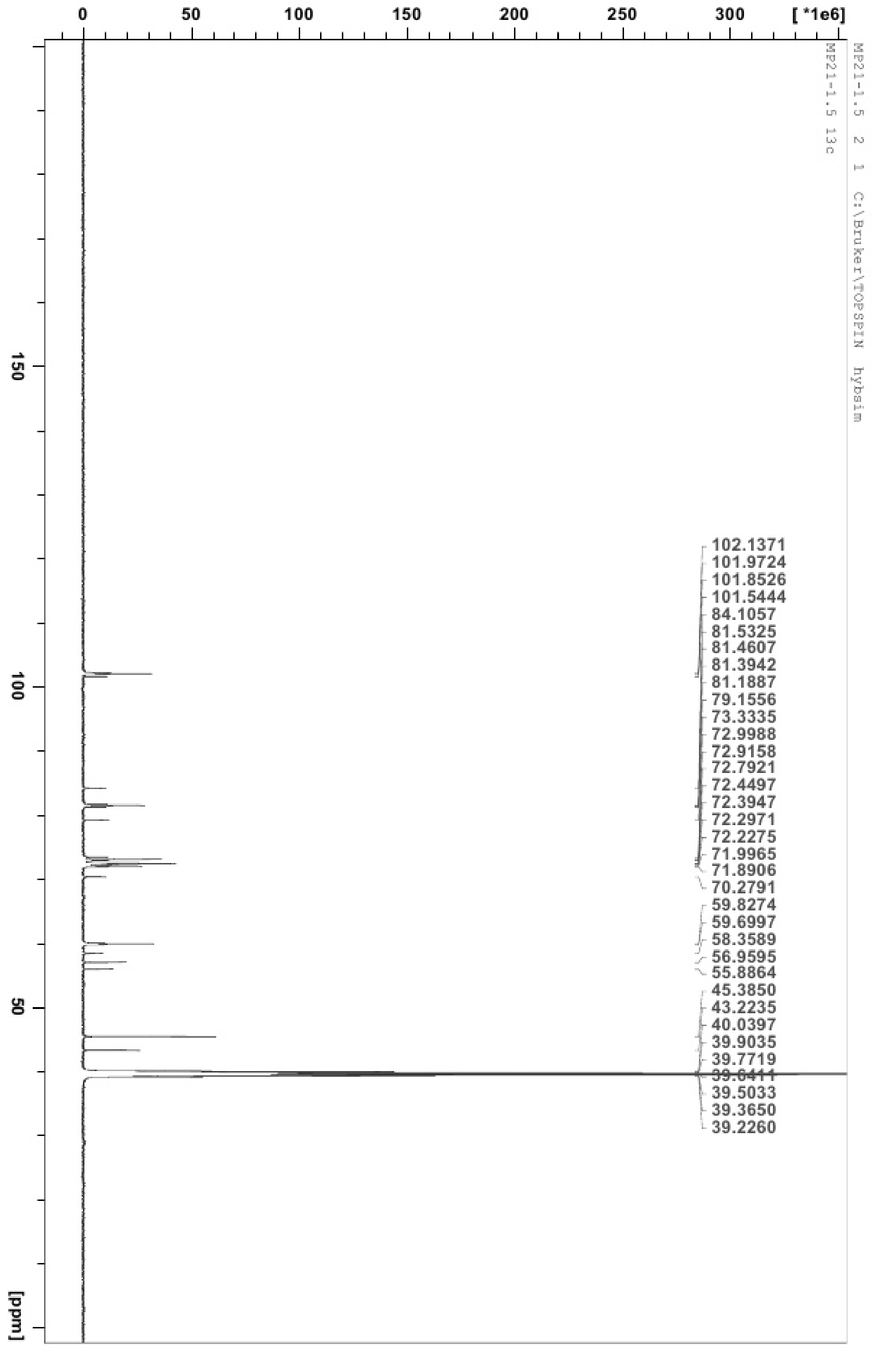

^{1}H NMR of compound **10** (600 MHz, D_2O)

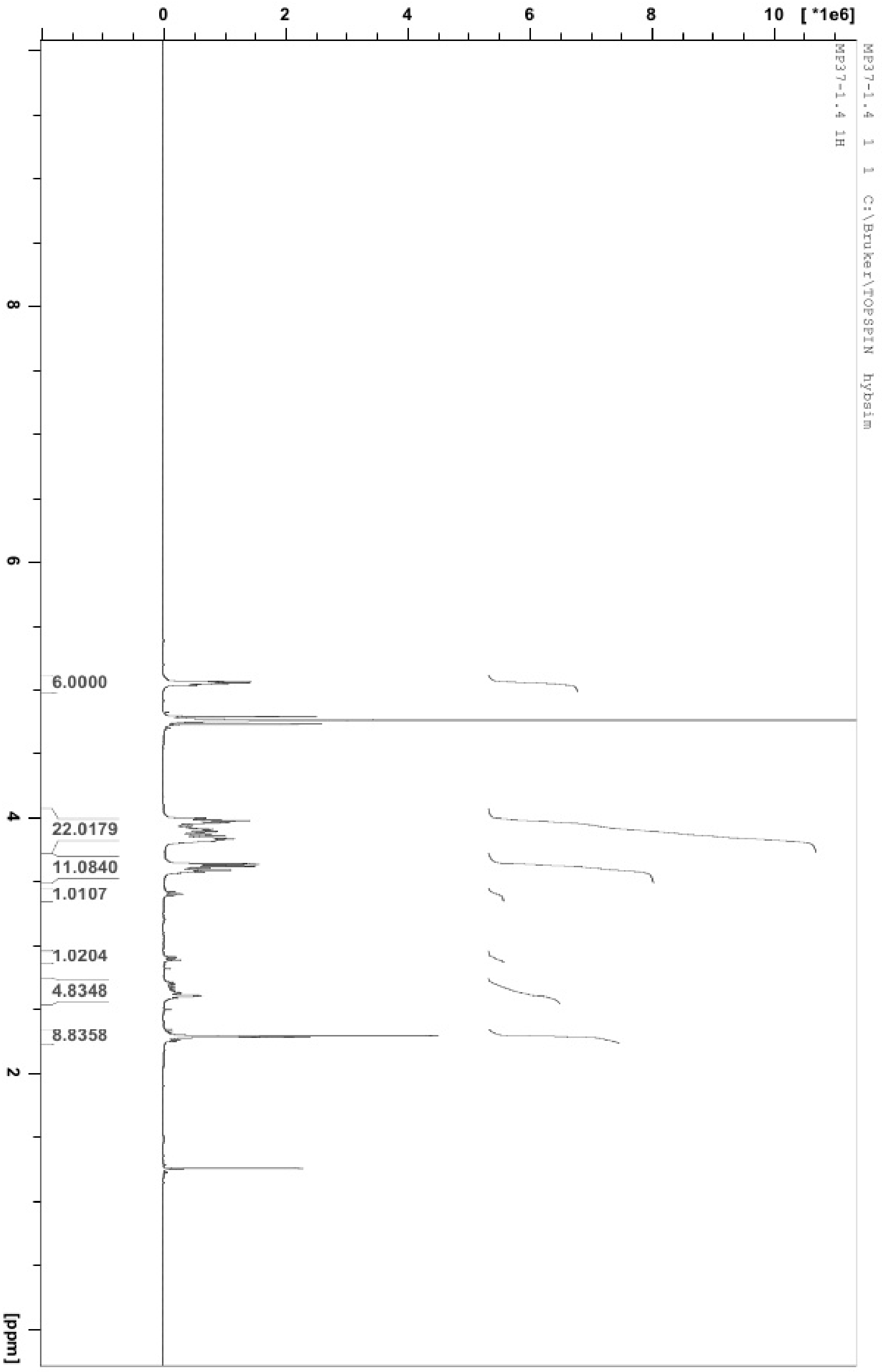

^{13}C NMR of compound **10** (150 MHz, D_2O)

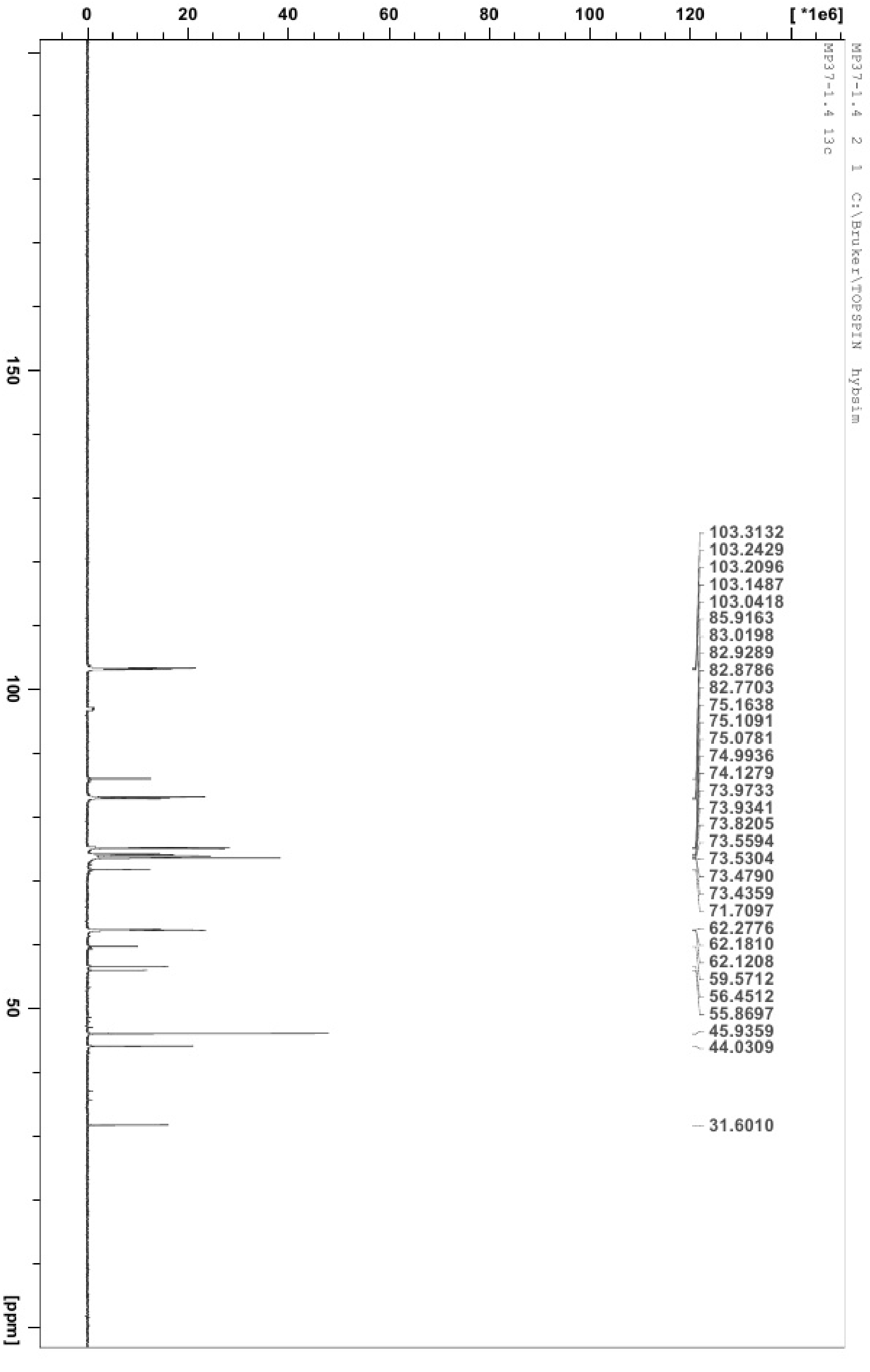

^{1}H NMR of compound **11** (600 MHz, D_2O)

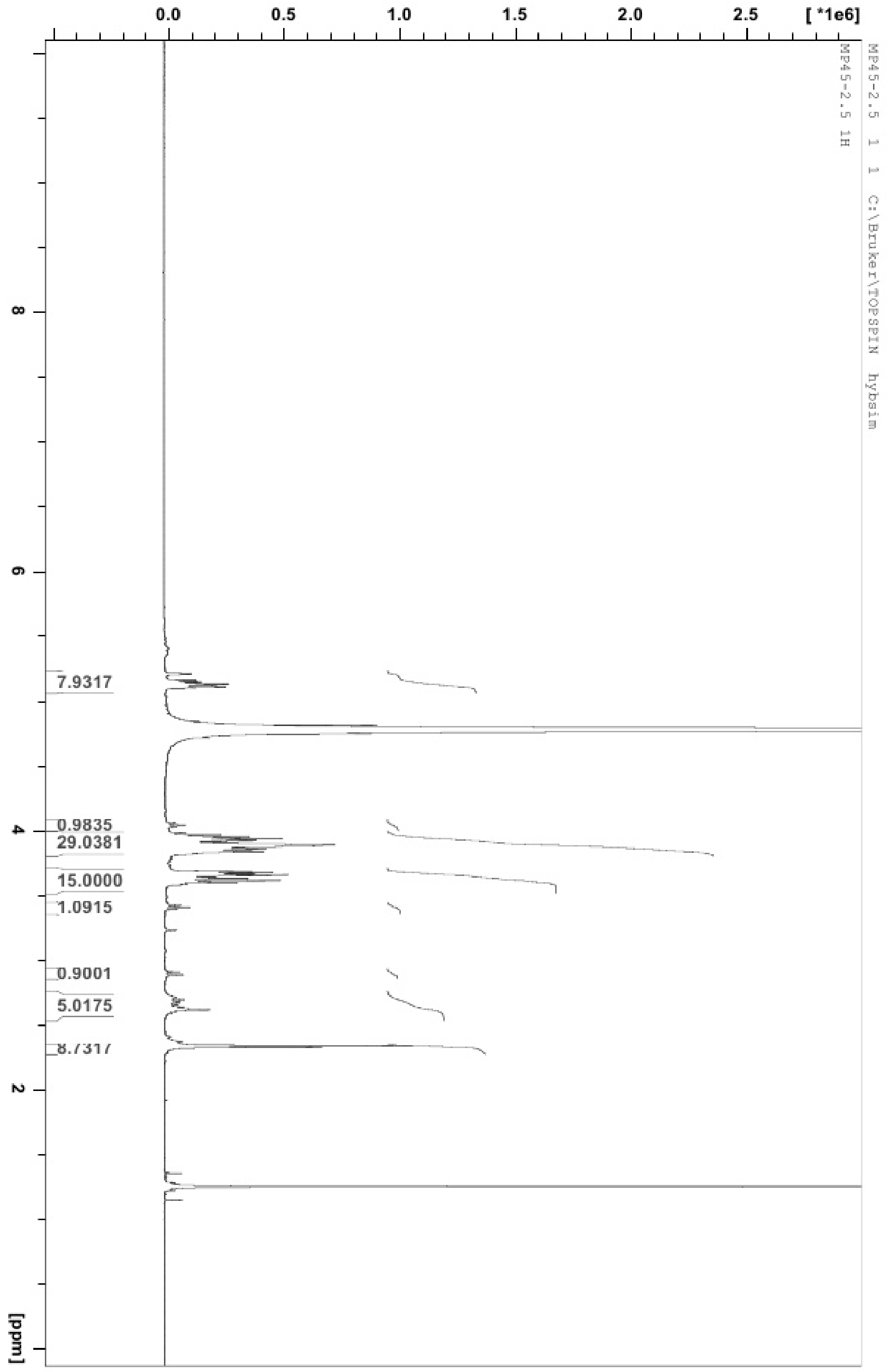

^{13}C NMR of compound **11** (150 MHz, D_2O)

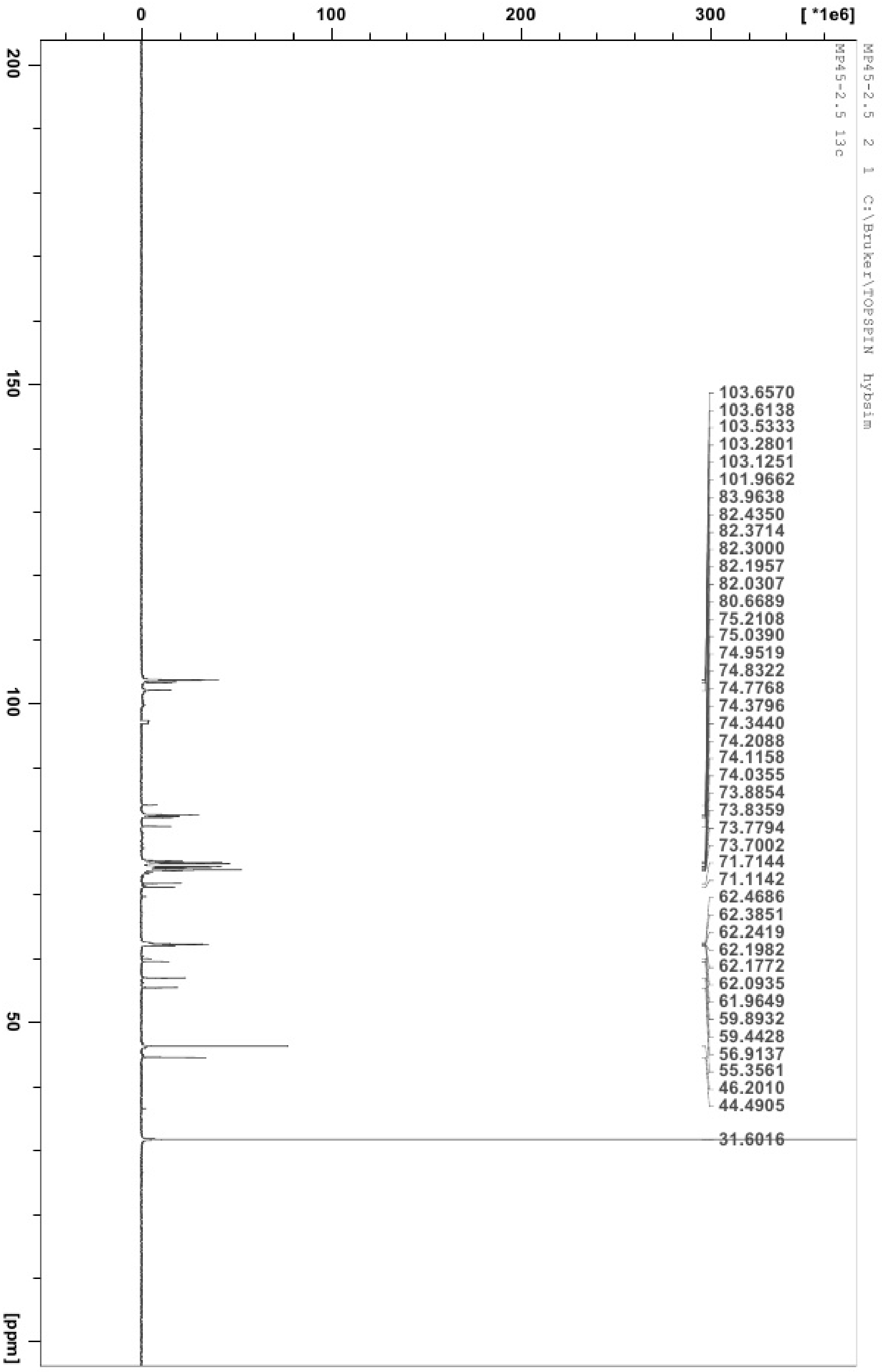

^{1}H NMR of compound **12** (600 MHz, D_2O)

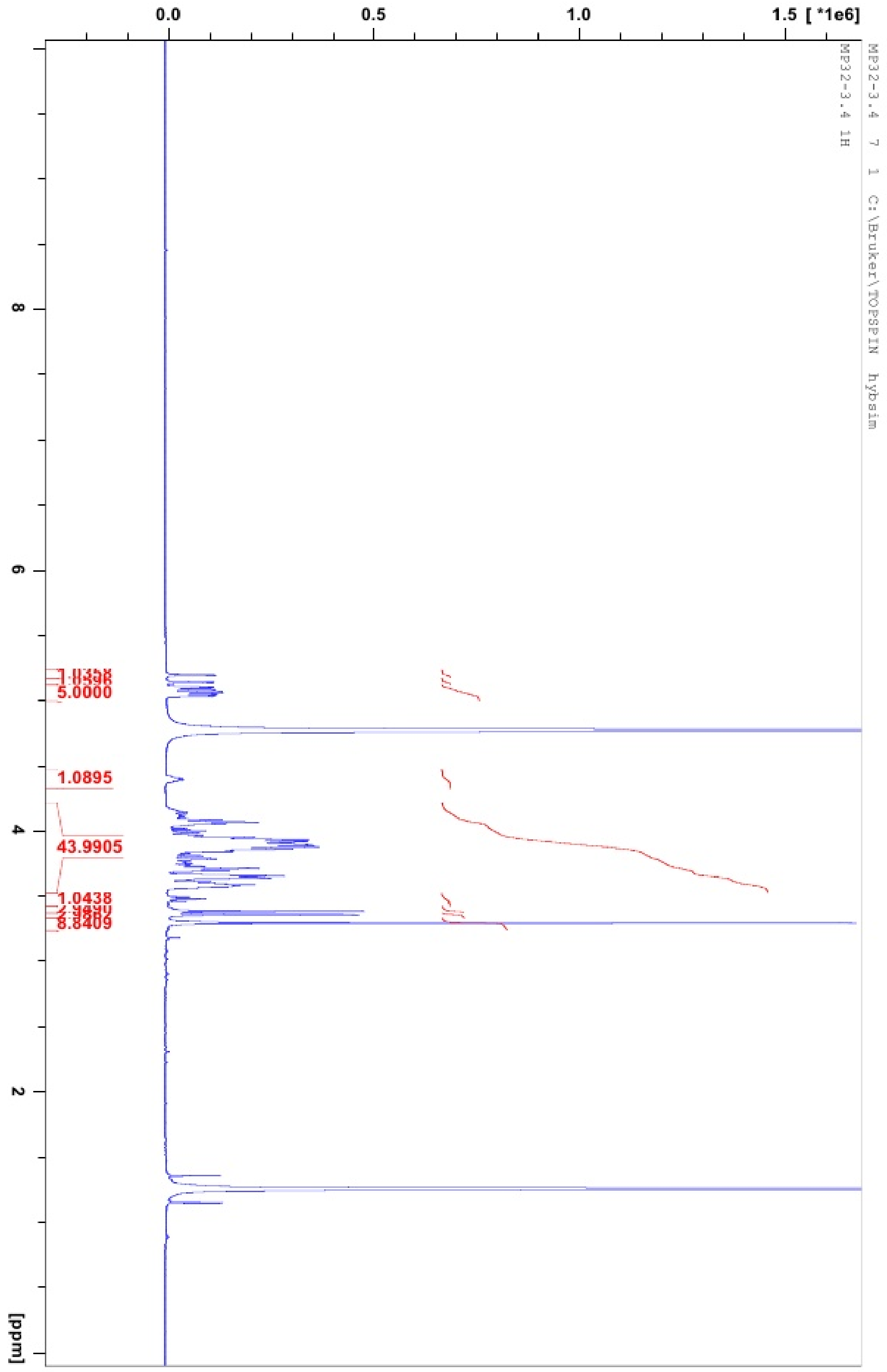

^{13}C NMR of compound **12** (150 MHz, D_2O)

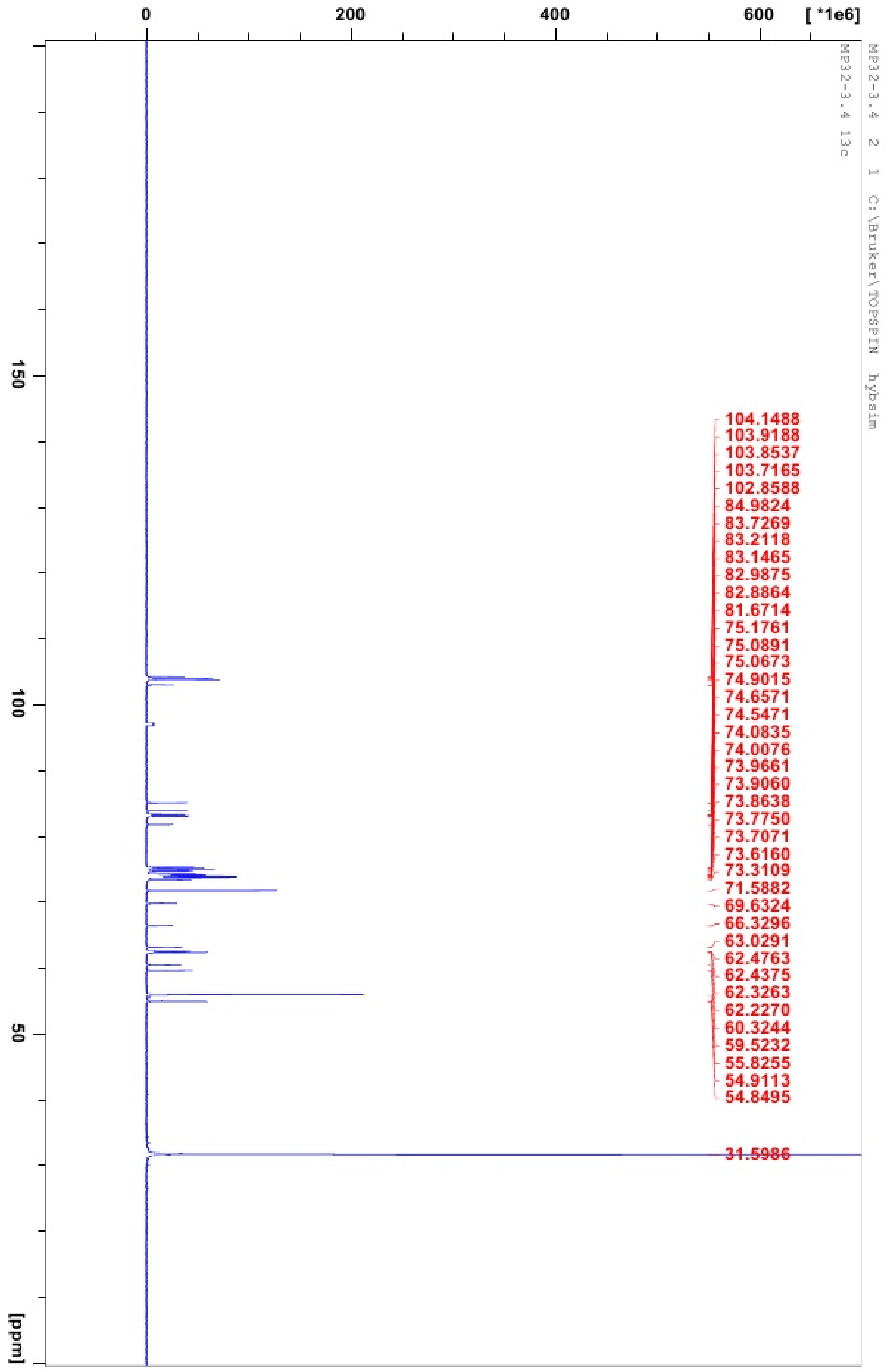

^{1}H NMR of compound **13** (600 MHz, D_2O)

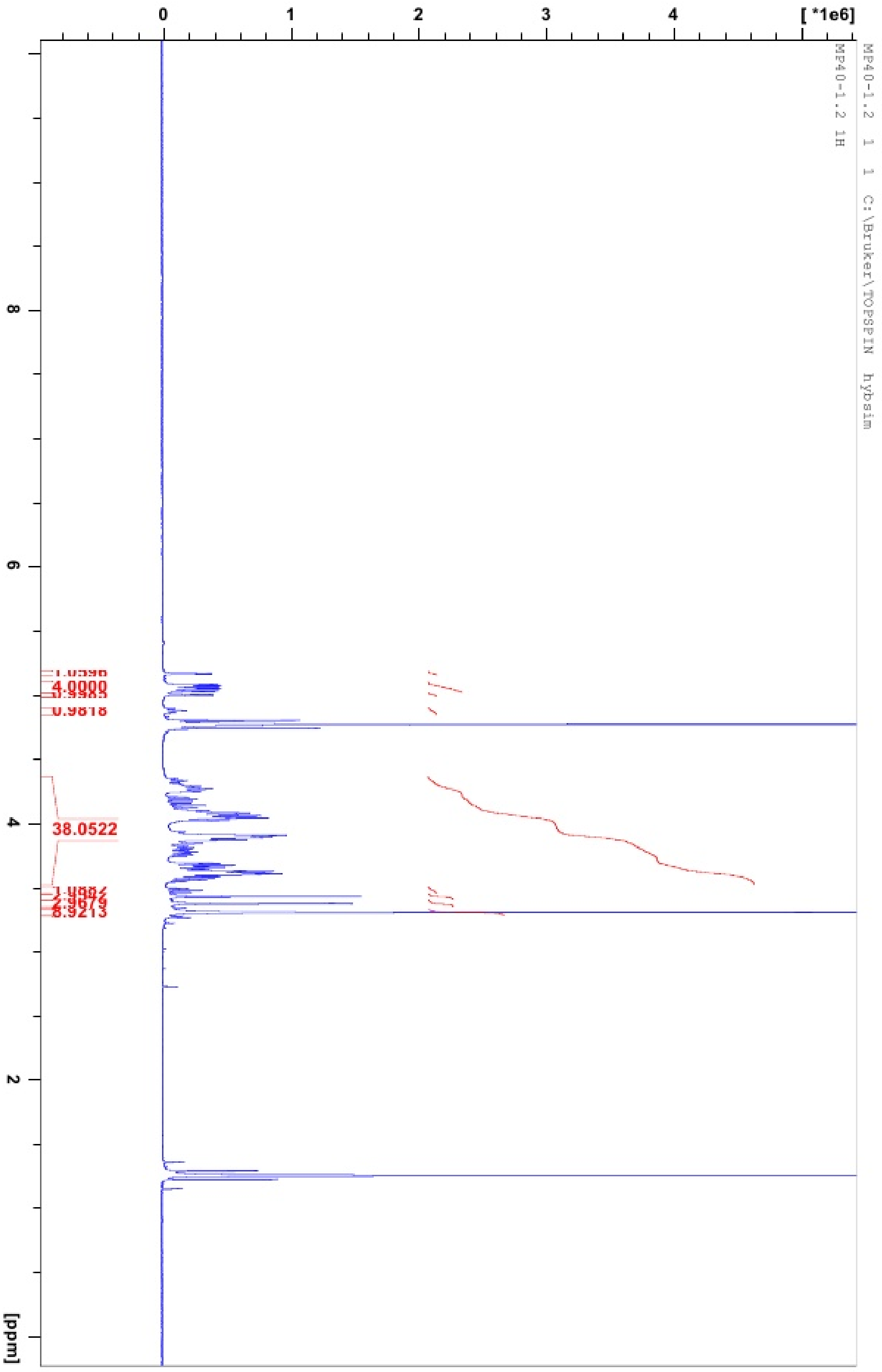

^{13}C NMR of compound **13** (150 MHz, D_2O)

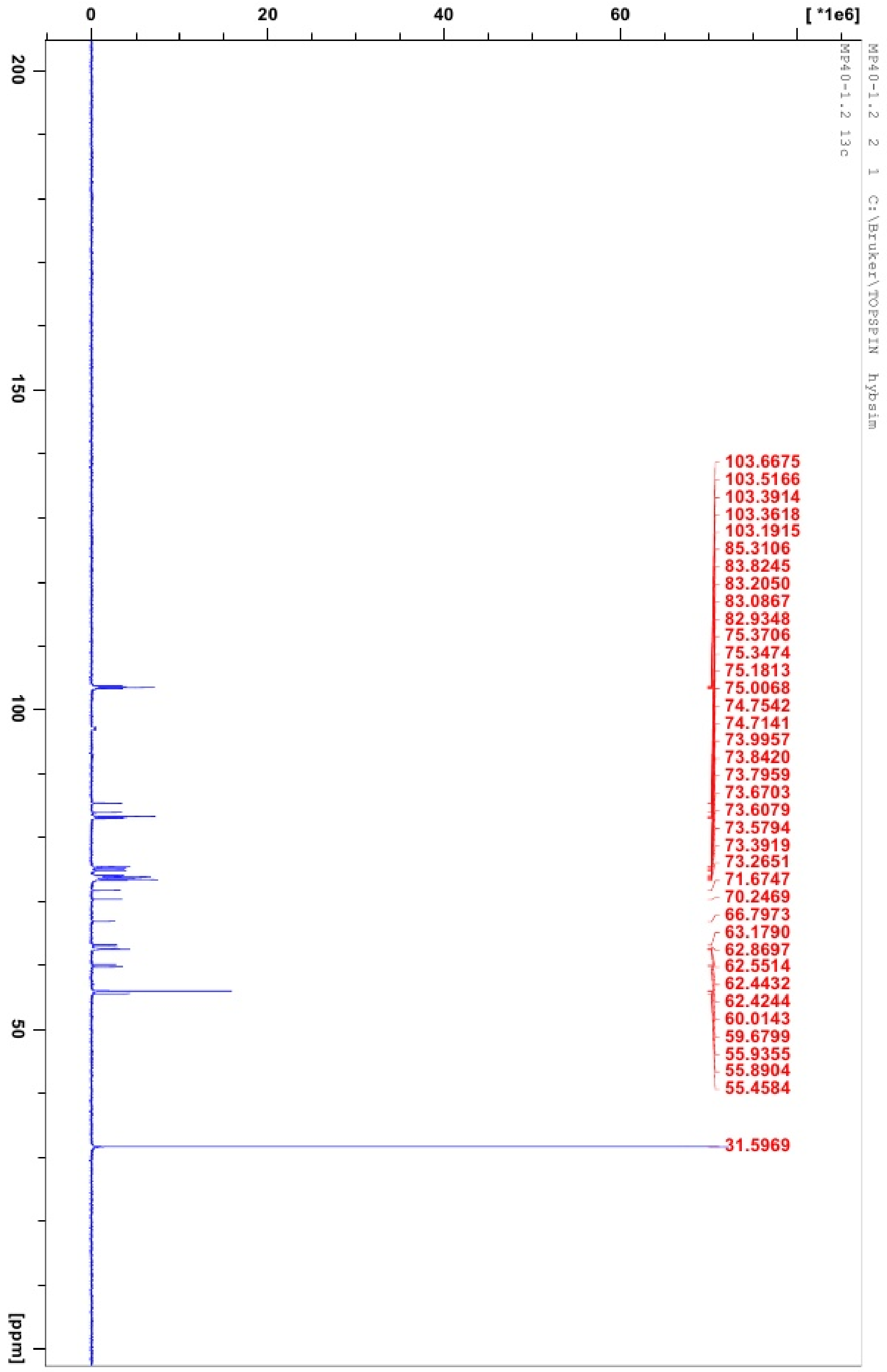

^{1}H NMR of compound **14** (600 MHz, D_2O)

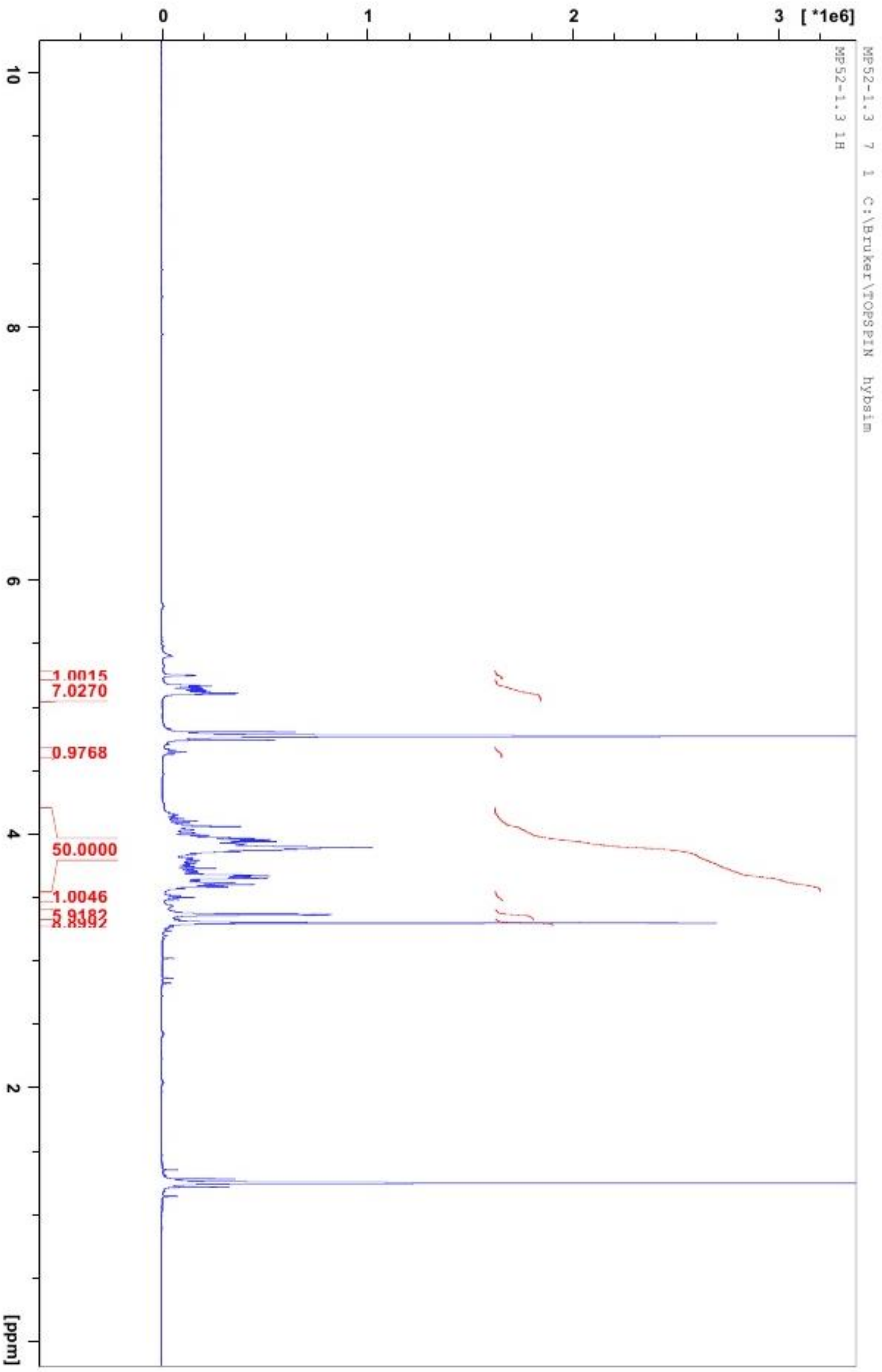

^{13}C NMR of compound **14** (150 MHz, D_2O)

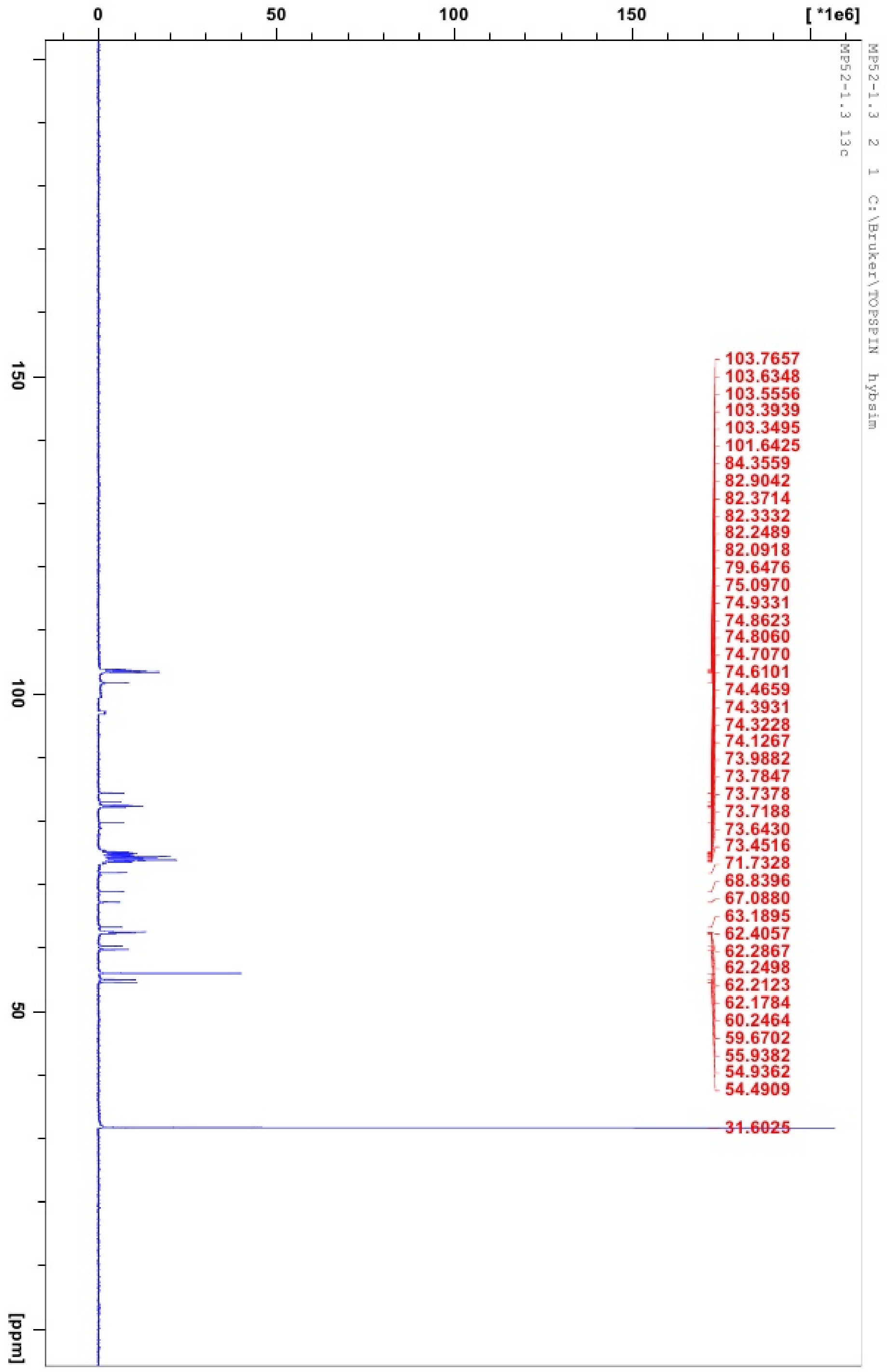

^{1}H NMR of compound **15** (600 MHz, DMSO-d_6)

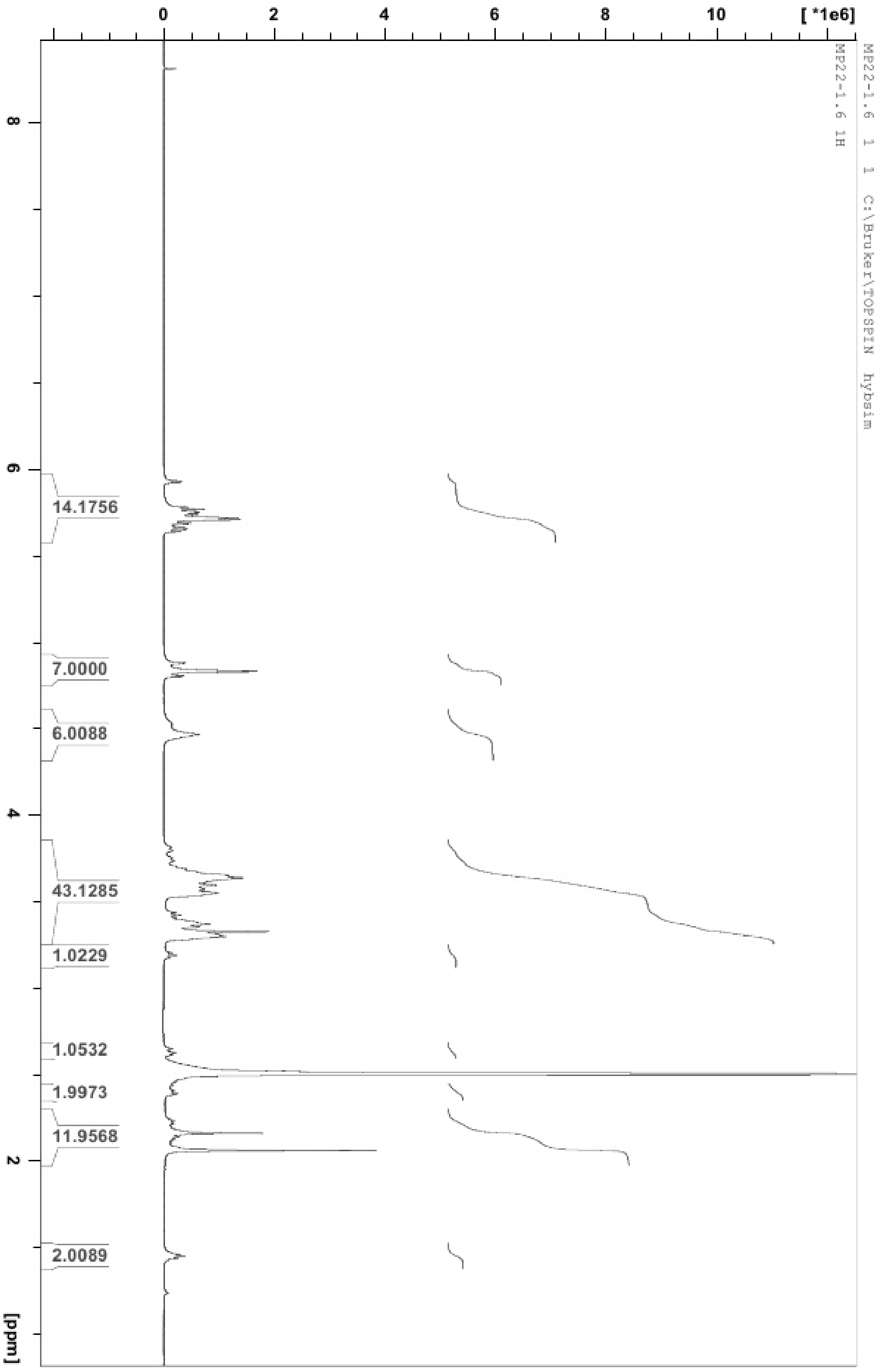

^{13}C NMR of compound **15** (150 MHz, DMSO-d_6)

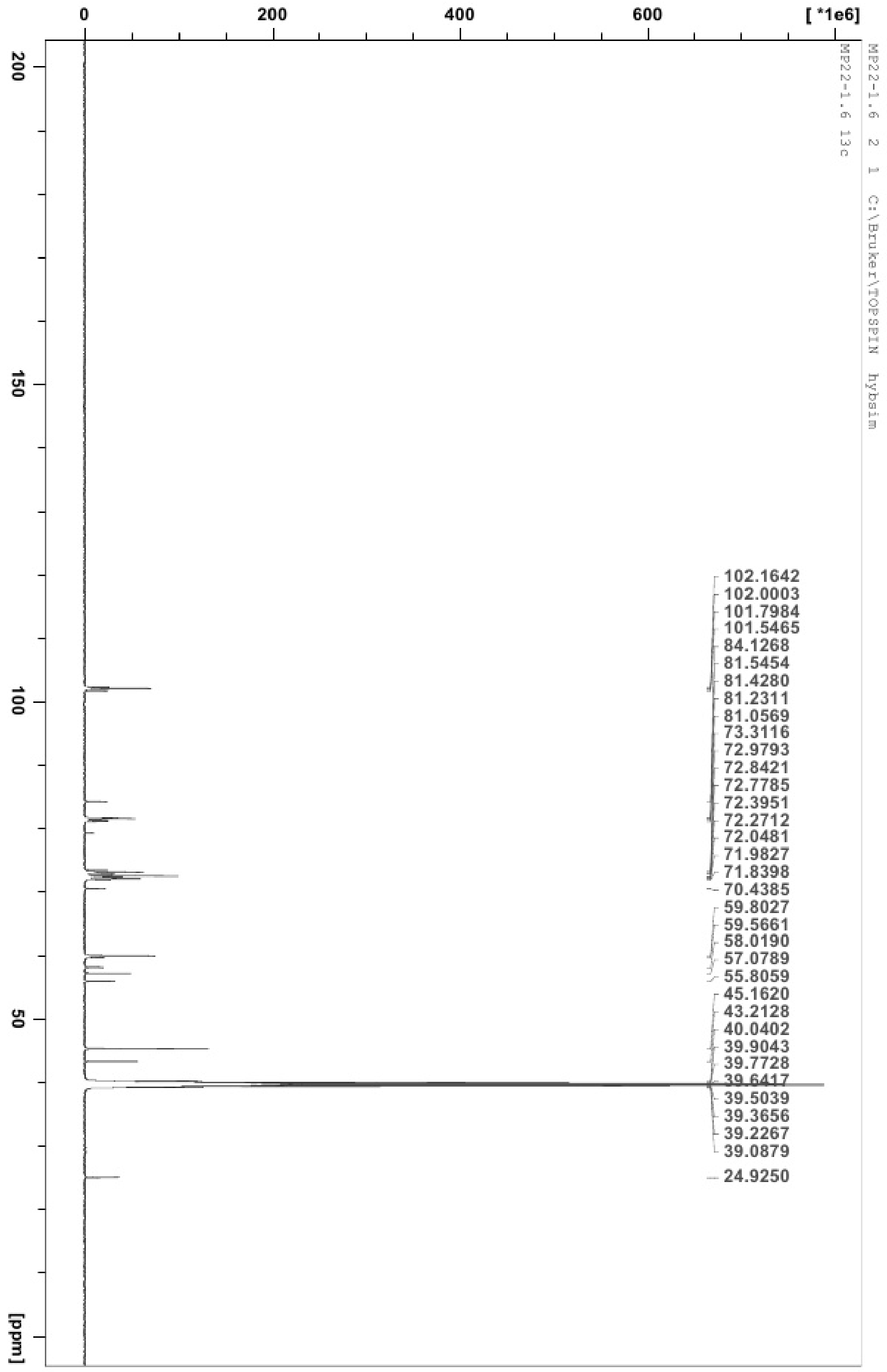

^{1}H NMR of compound **16** (600 MHz, D_2O)

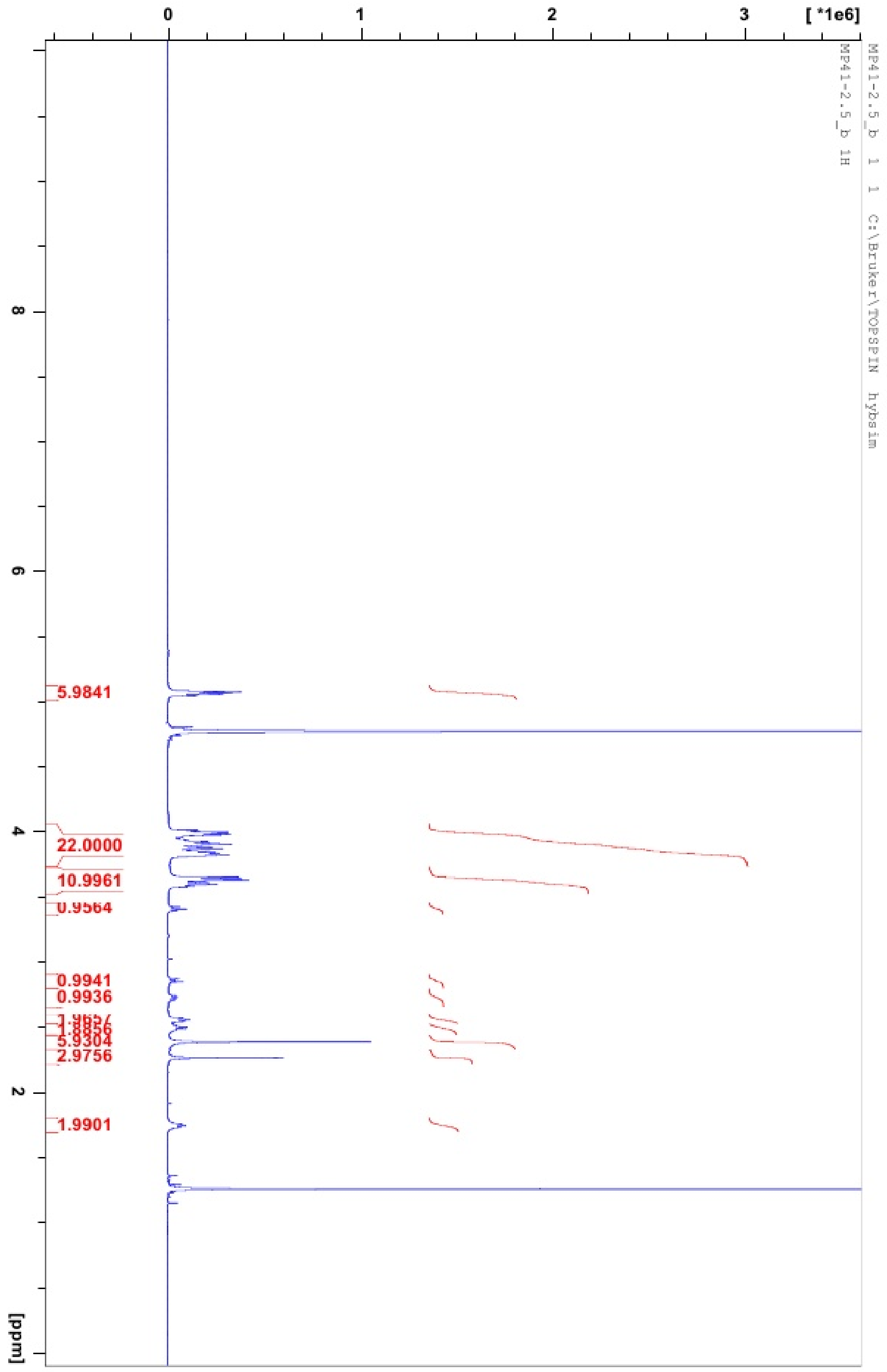

^{13}C NMR of compound **16** (150 MHz, D_2O)

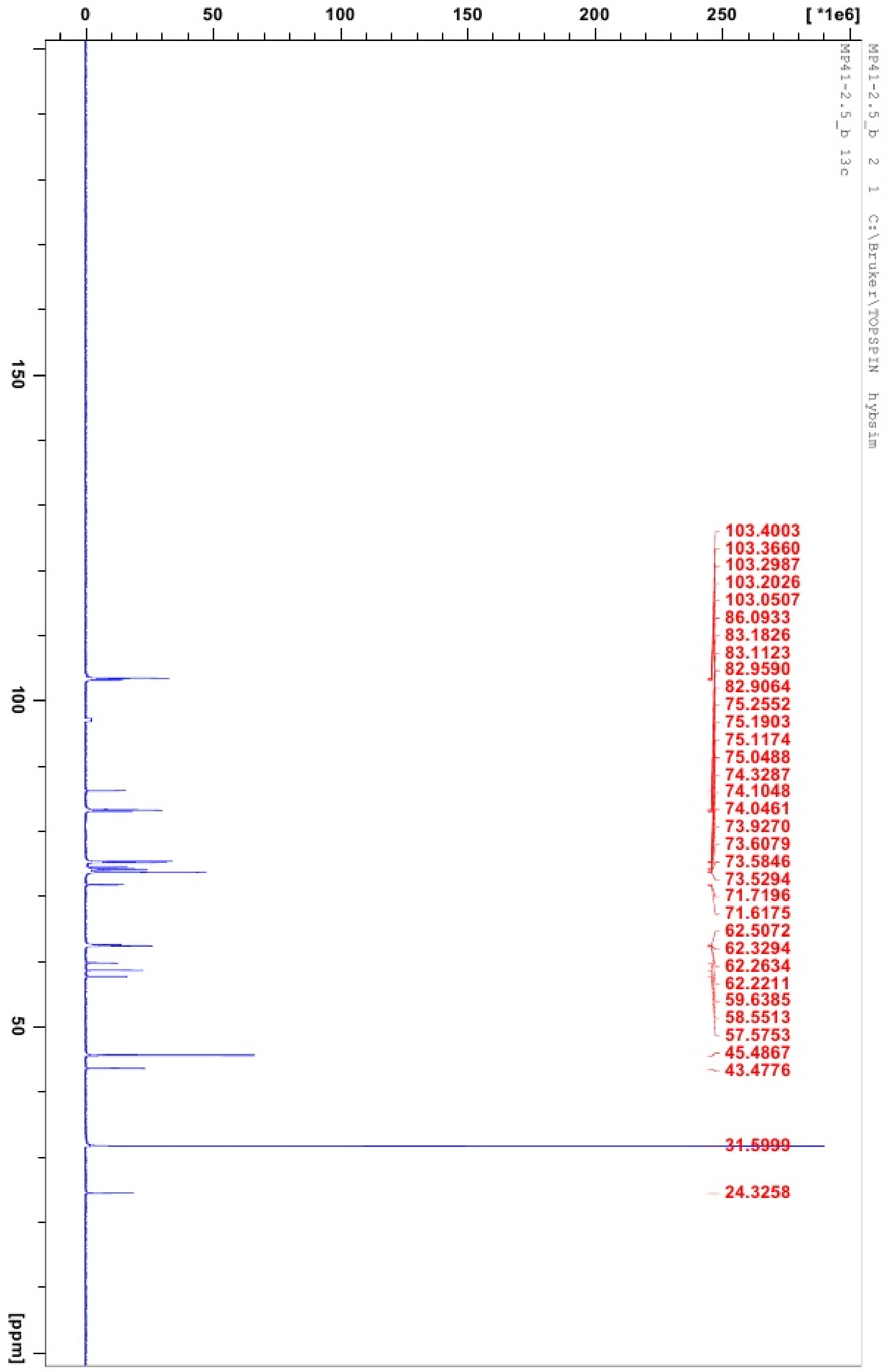

^{1}H NMR of compound **17** (600 MHz, D_2O)

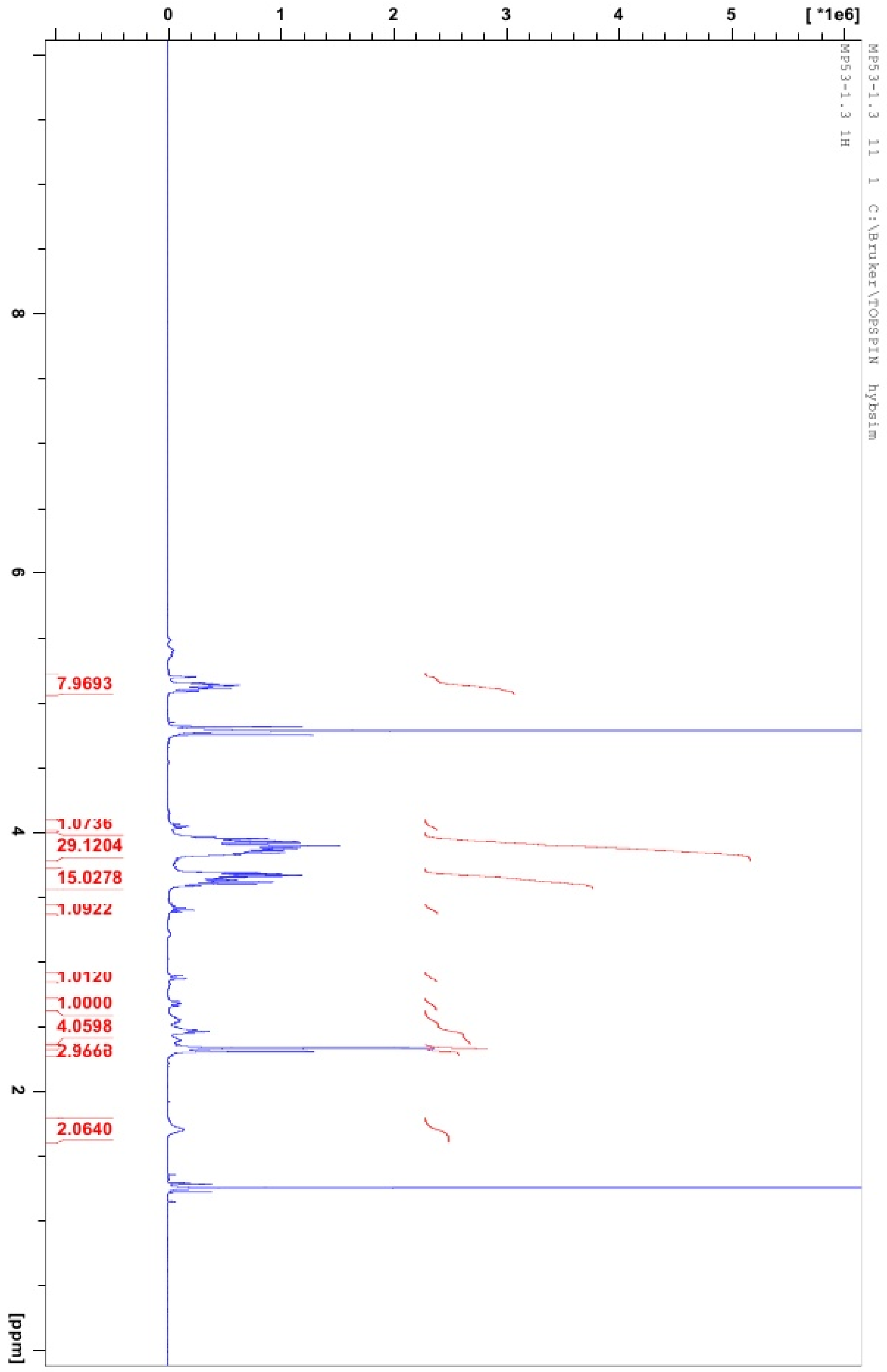

^{13}C NMR of compound **17** (150 MHz, D_2O)

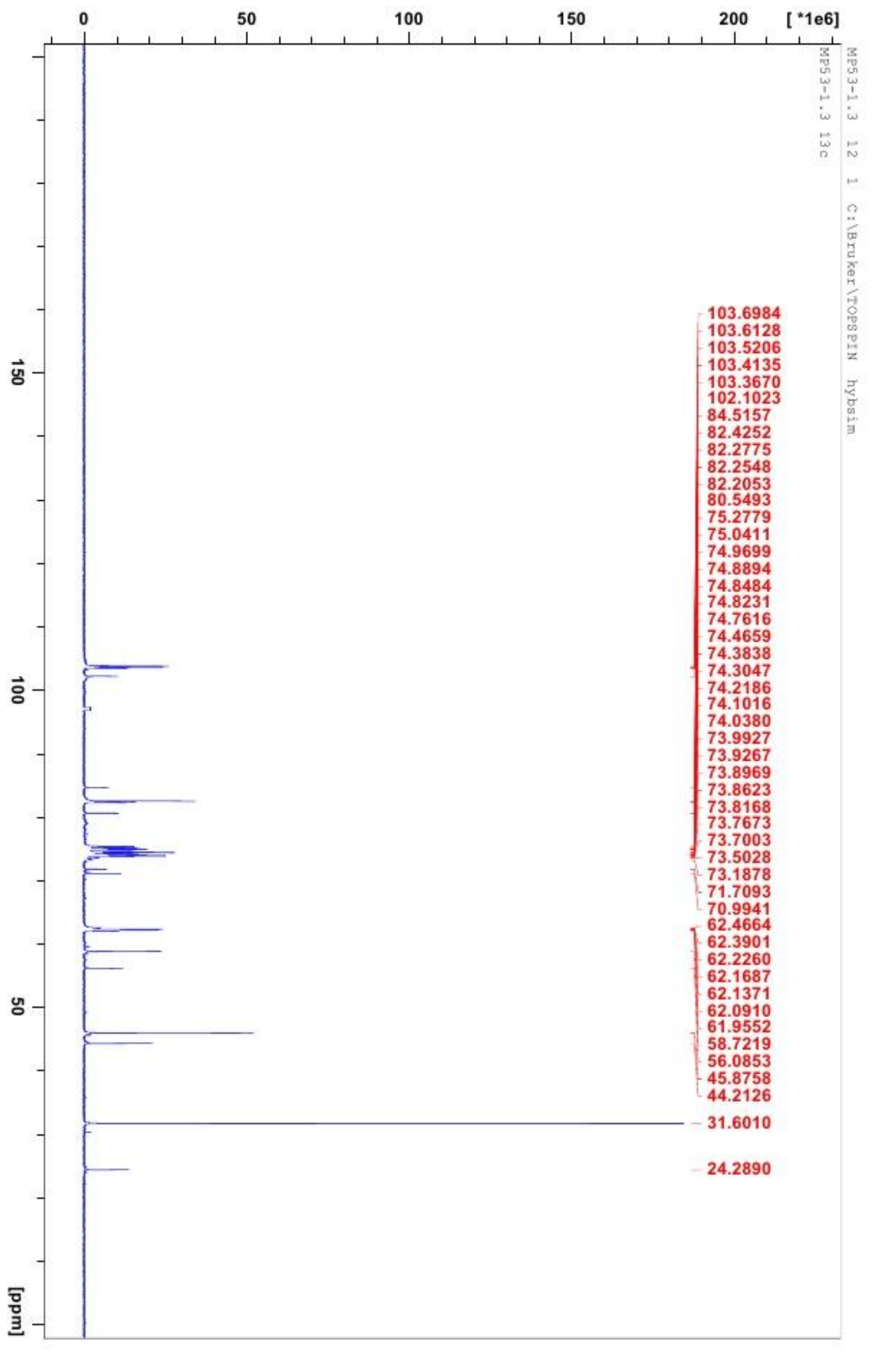

^{1}H NMR of compound **18** (600 MHz, D_2O)

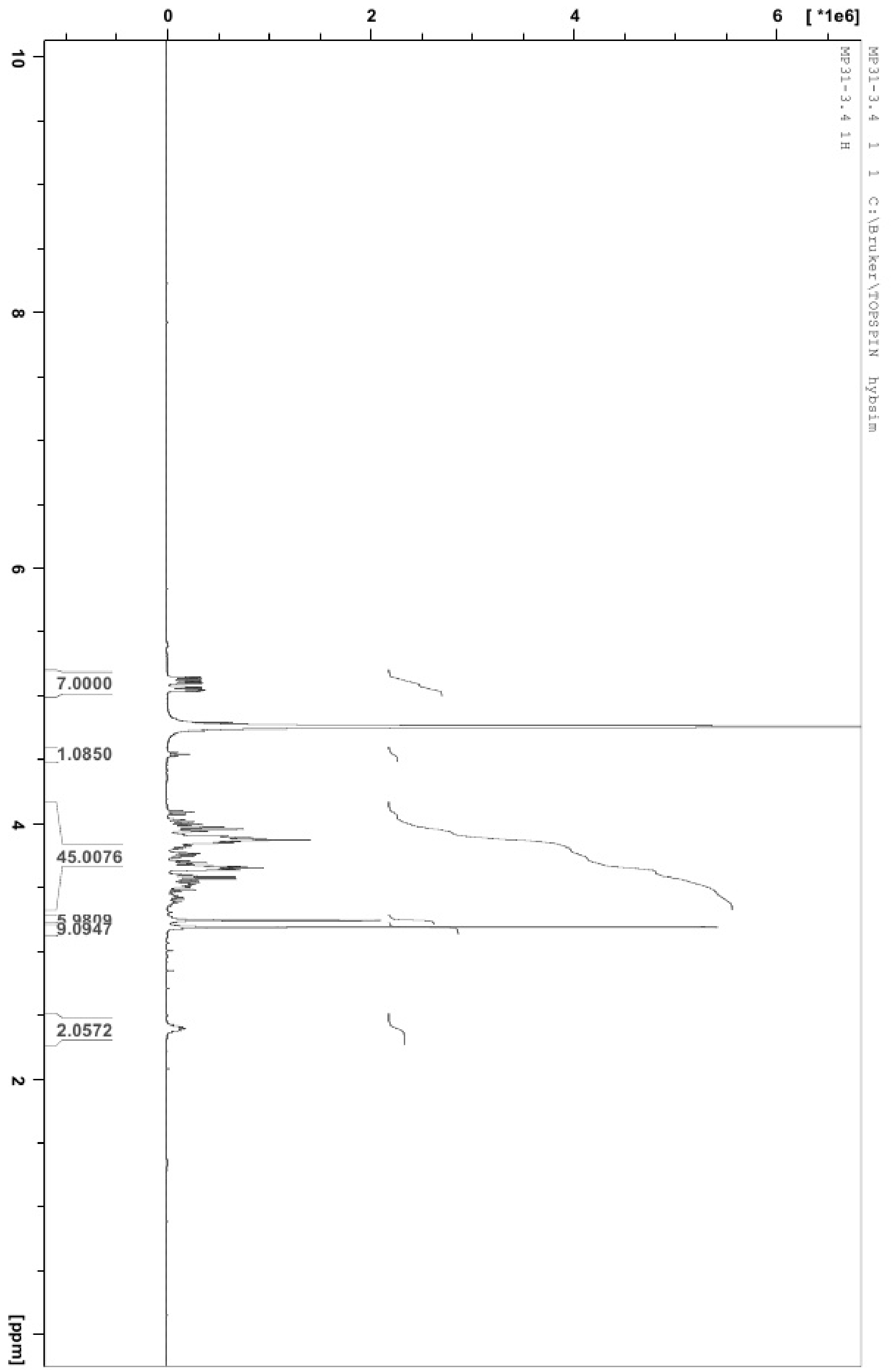

^{13}C NMR of compound **18** (150 MHz, D_2O)

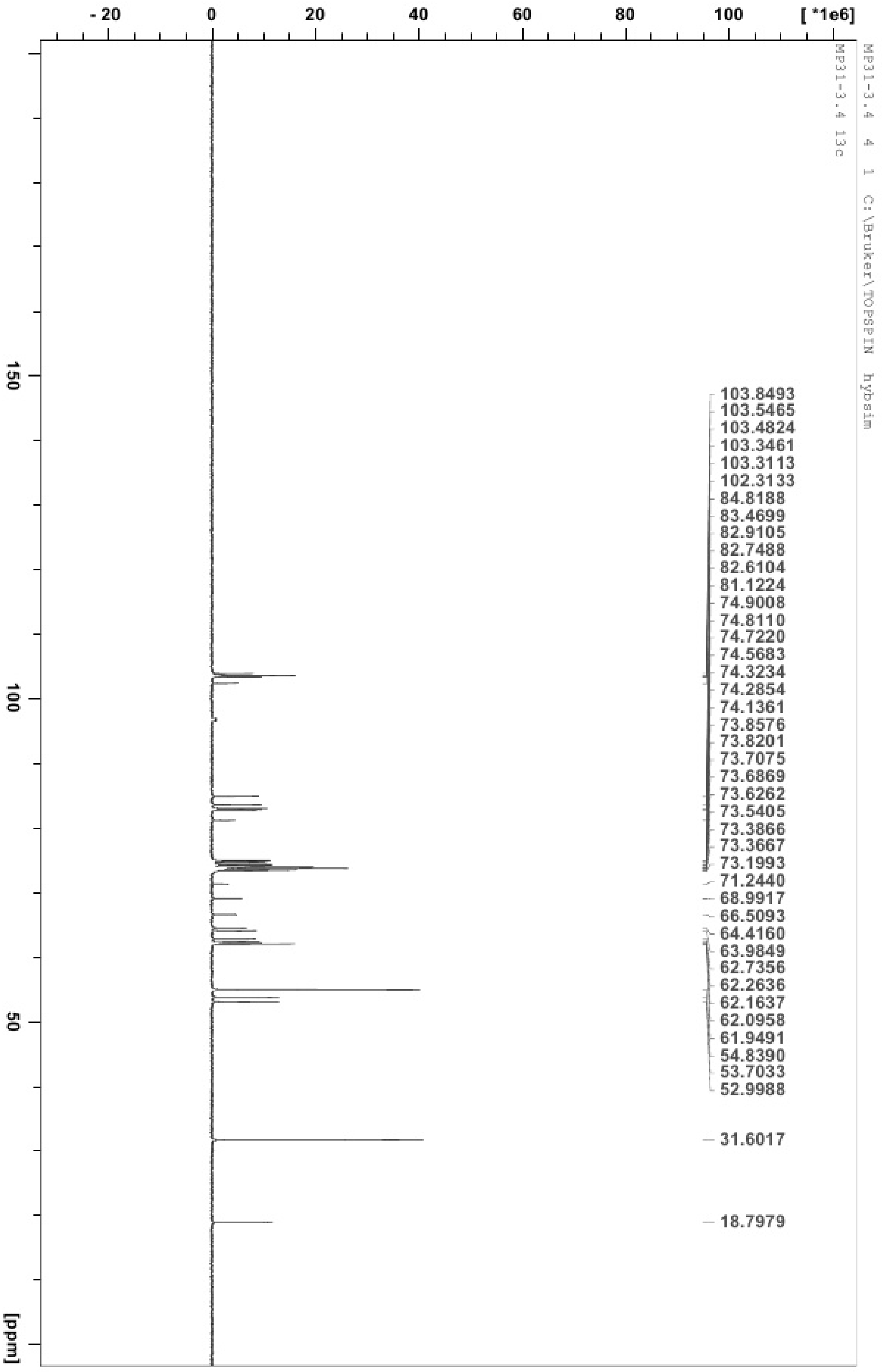

^{1}H NMR of compound **19** (600 MHz, D_2O)

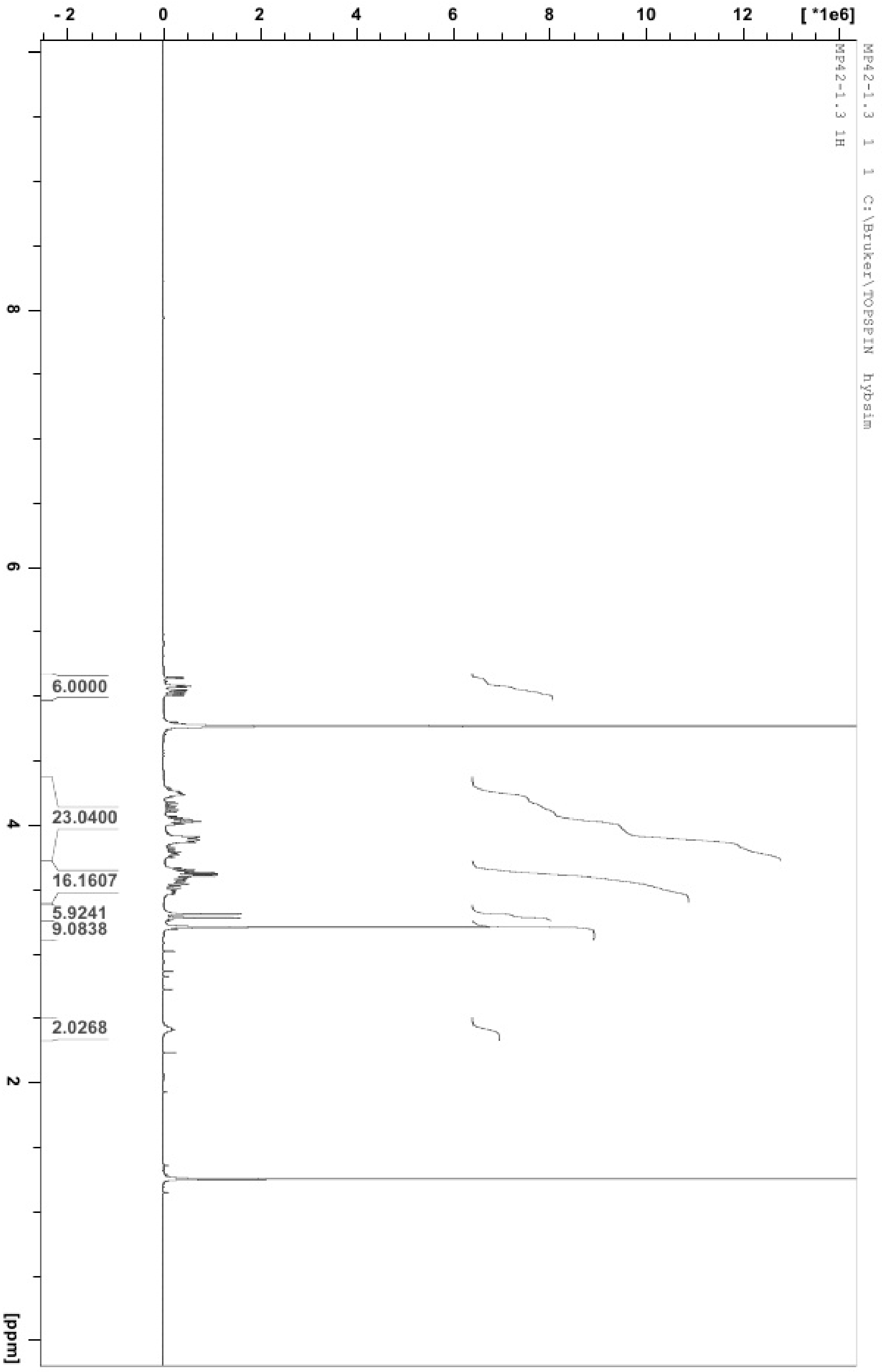

^{13}C NMR of compound **19** (150 MHz, D_2O)

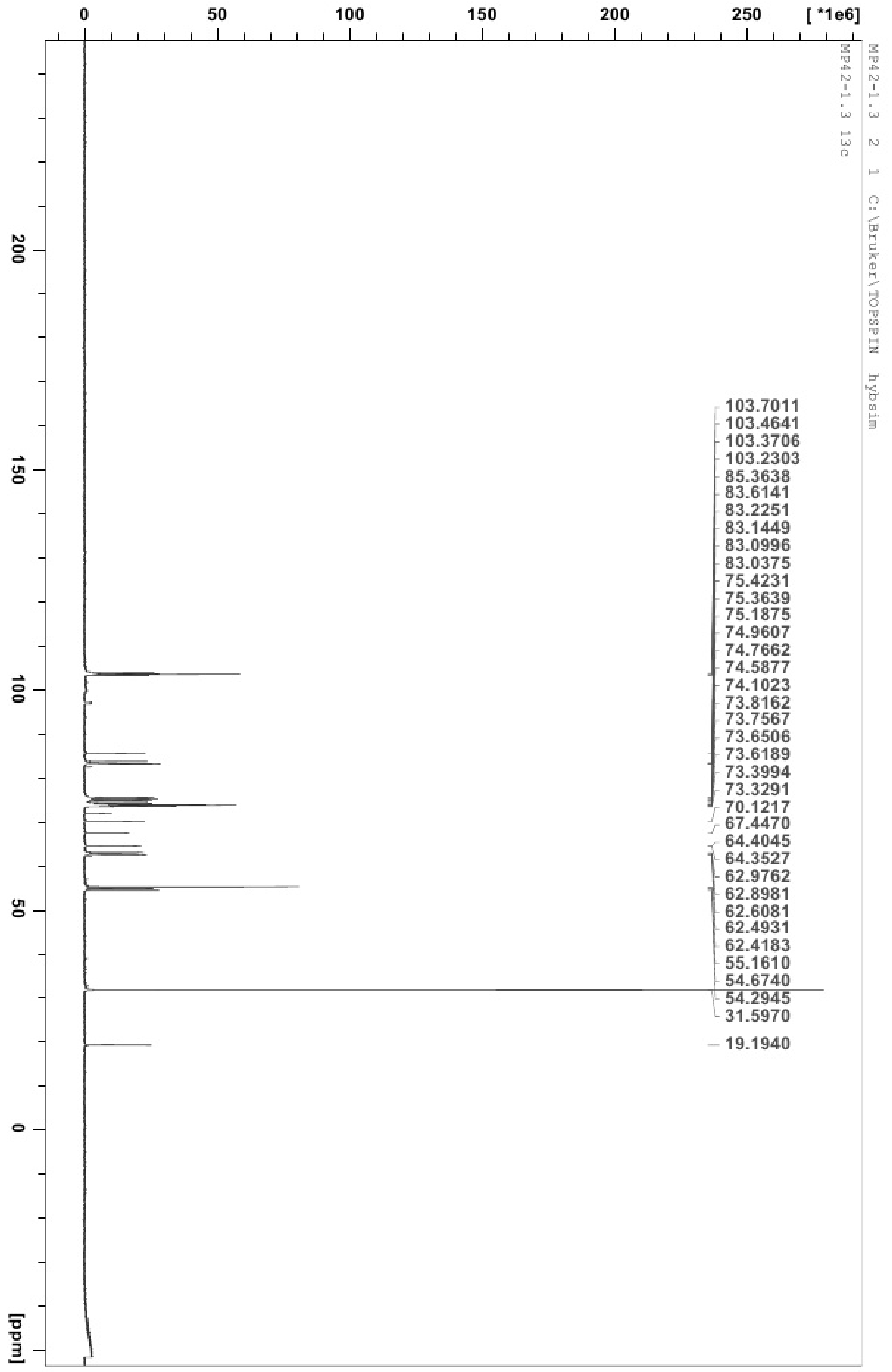

^{1}H NMR of compound **20** (600 MHz, D_2O)

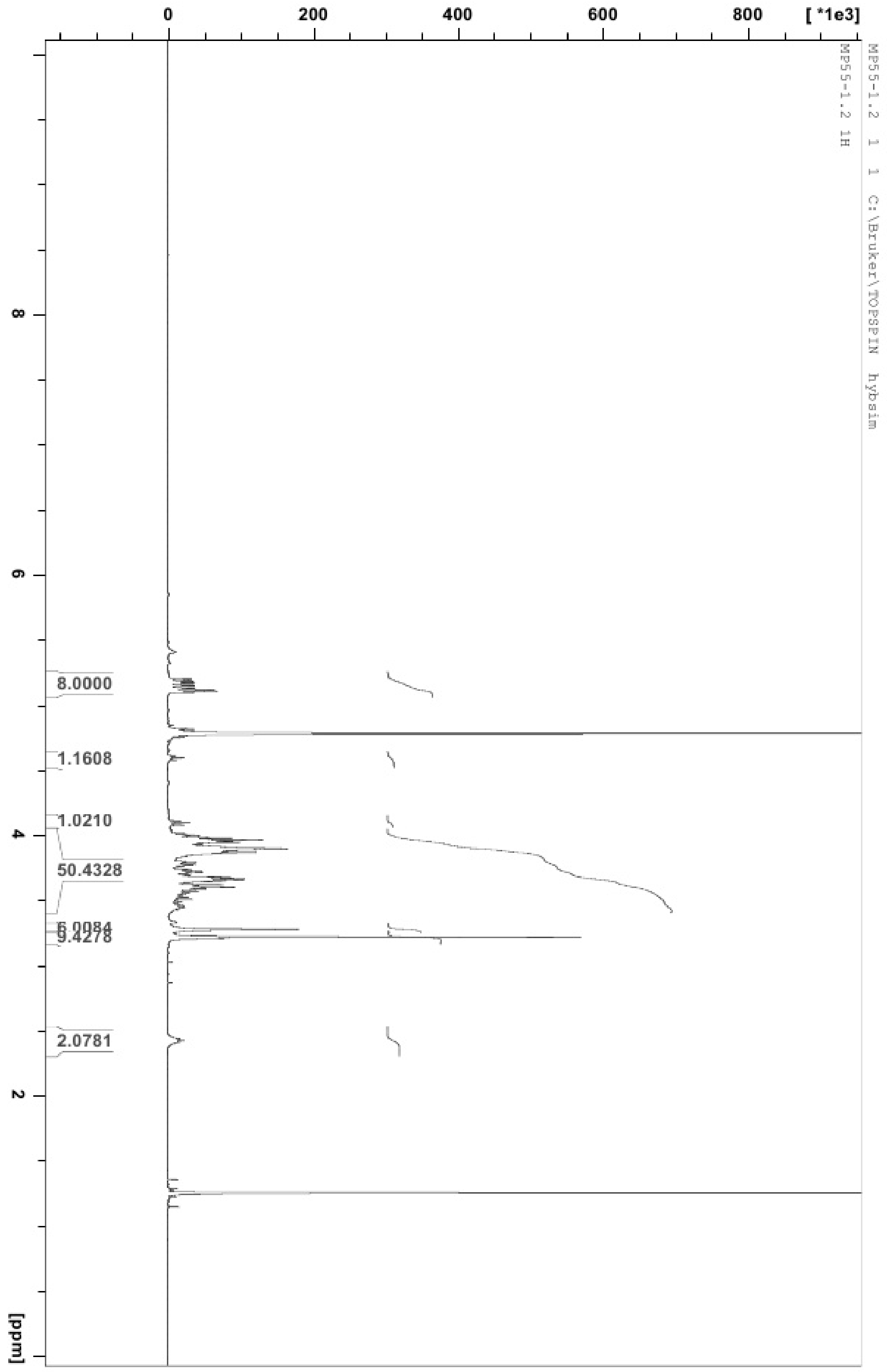

^{13}C NMR of compound **20** (150 MHz, D_2O)

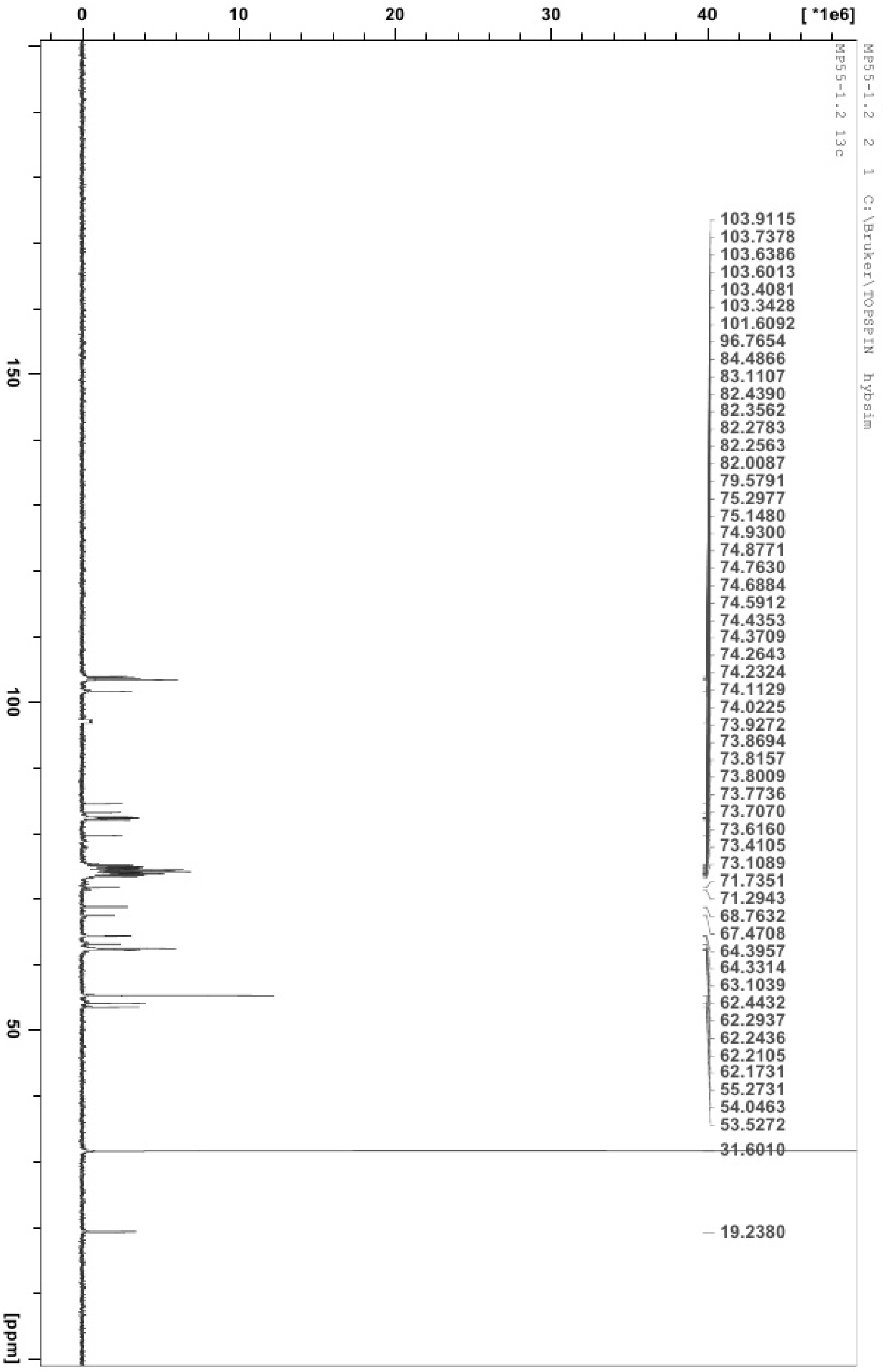

^{1}H NMR of compound **25** (600 MHz, D_2O)

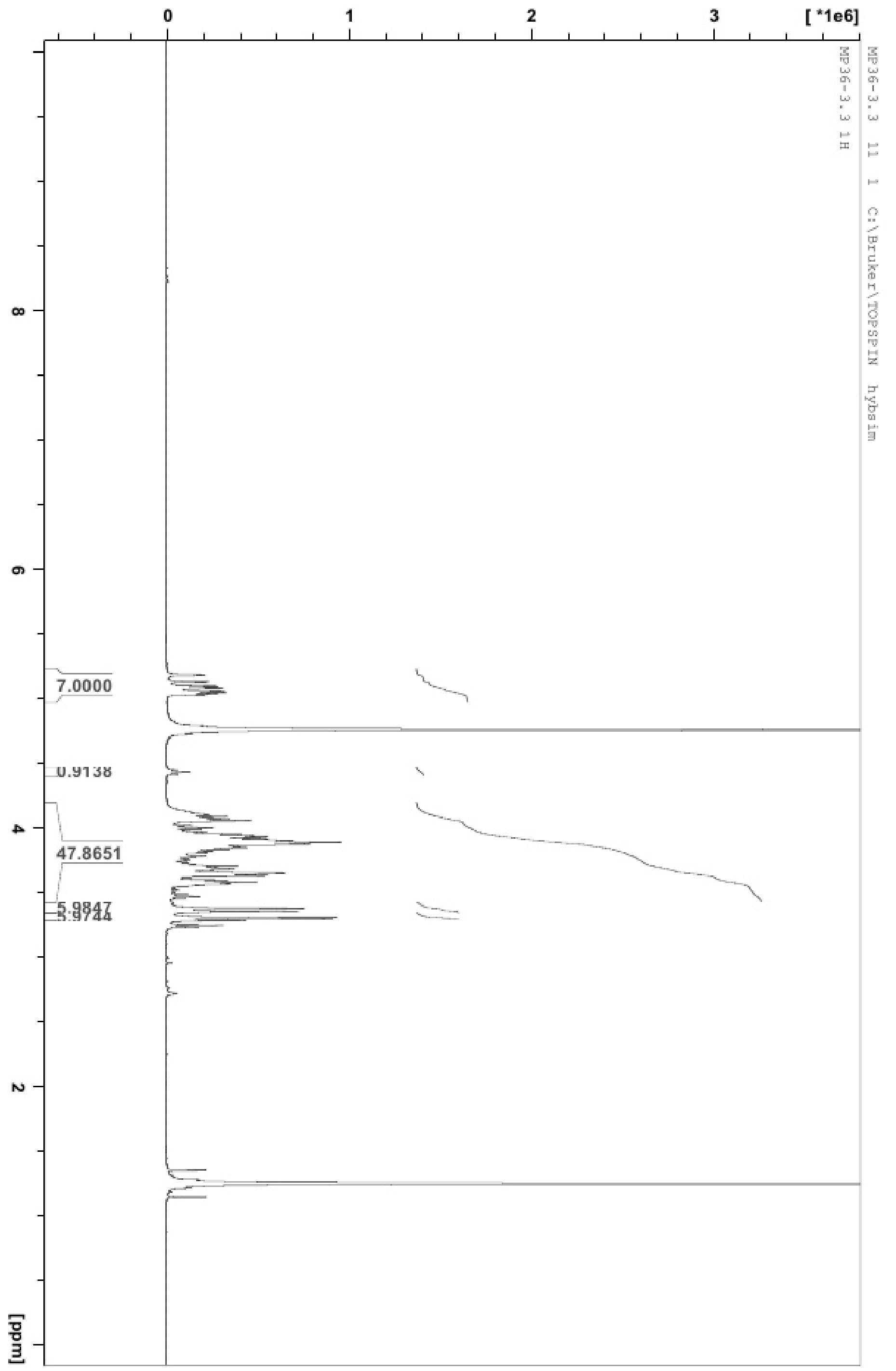

^{13}C NMR of compound **25** (150 MHz, D_2O)

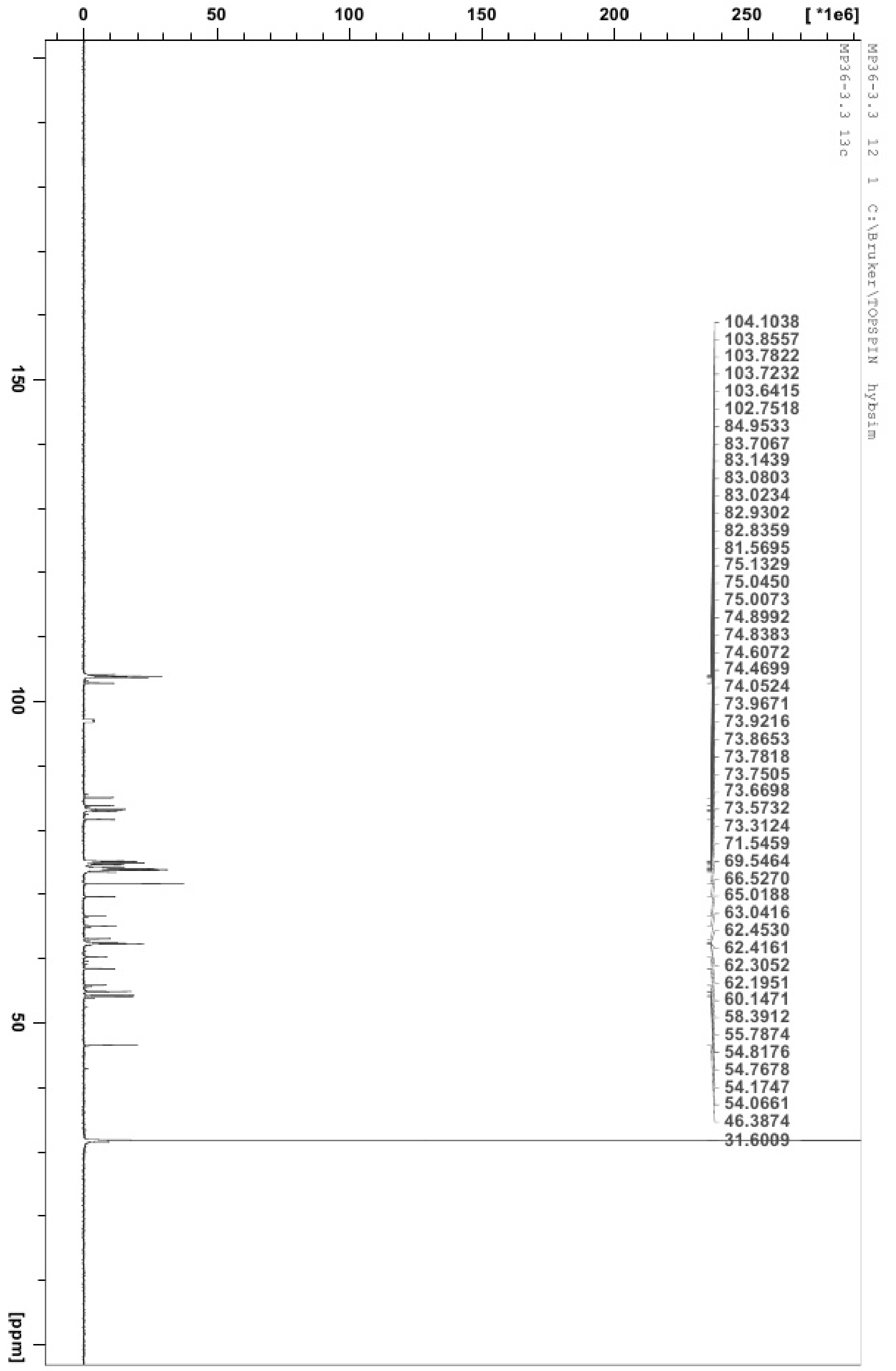

^{1}H NMR of compound **26** (600 MHz, D_2O)

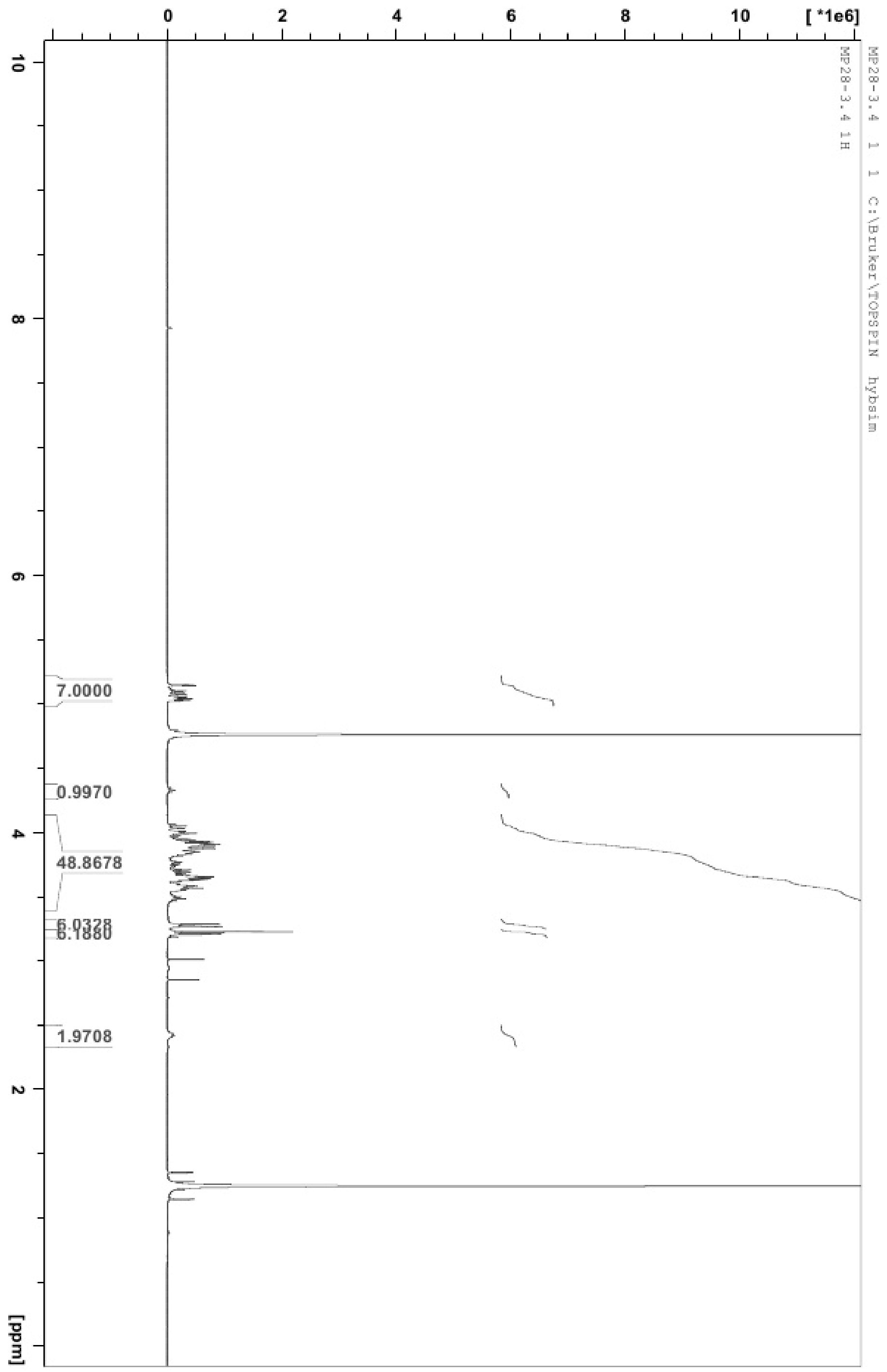

^{13}C NMR of compound **26** (150 MHz, D_2O)

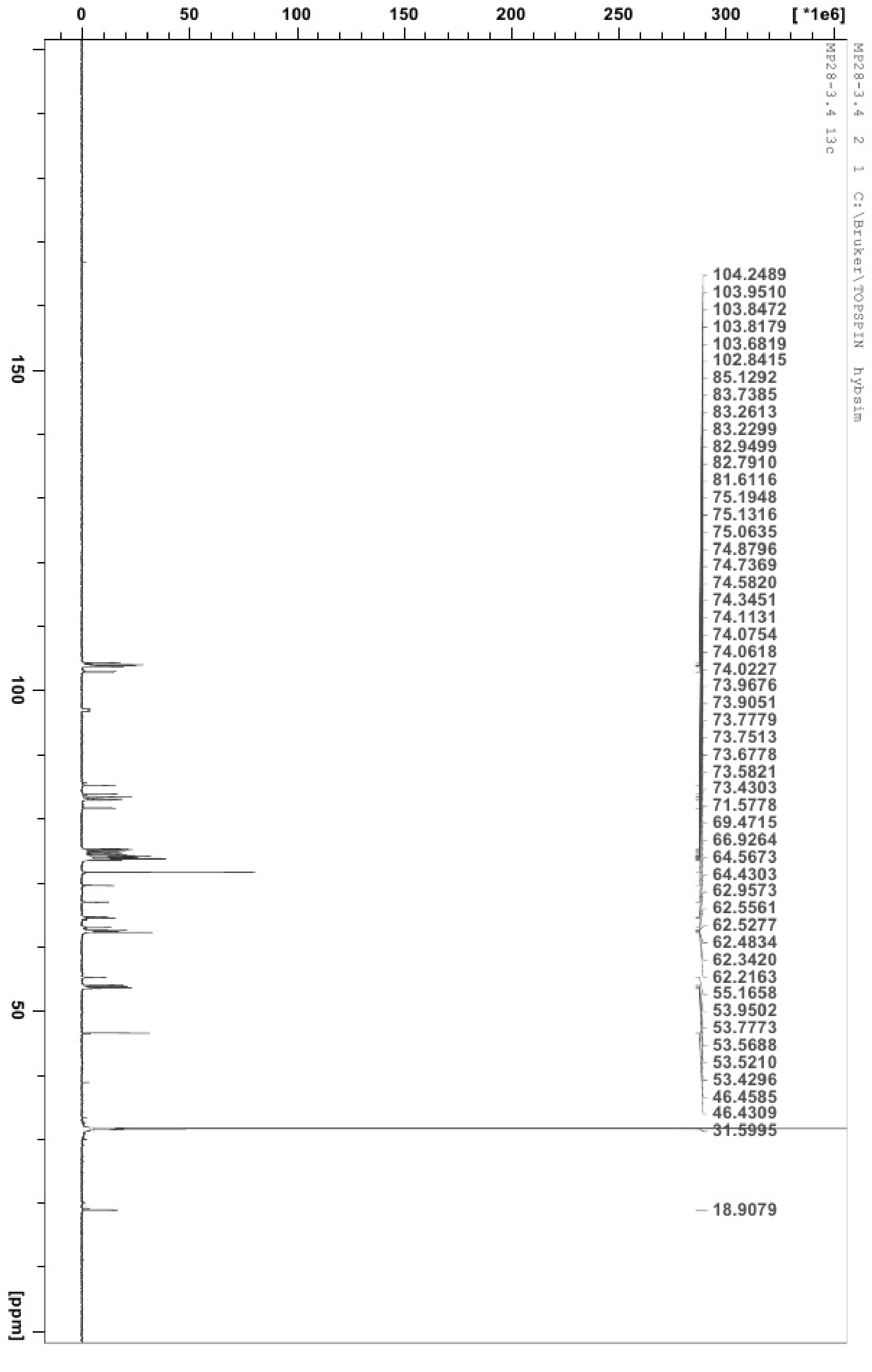

^{1}H NMR of compound **27** (600 MHz, D_2O)

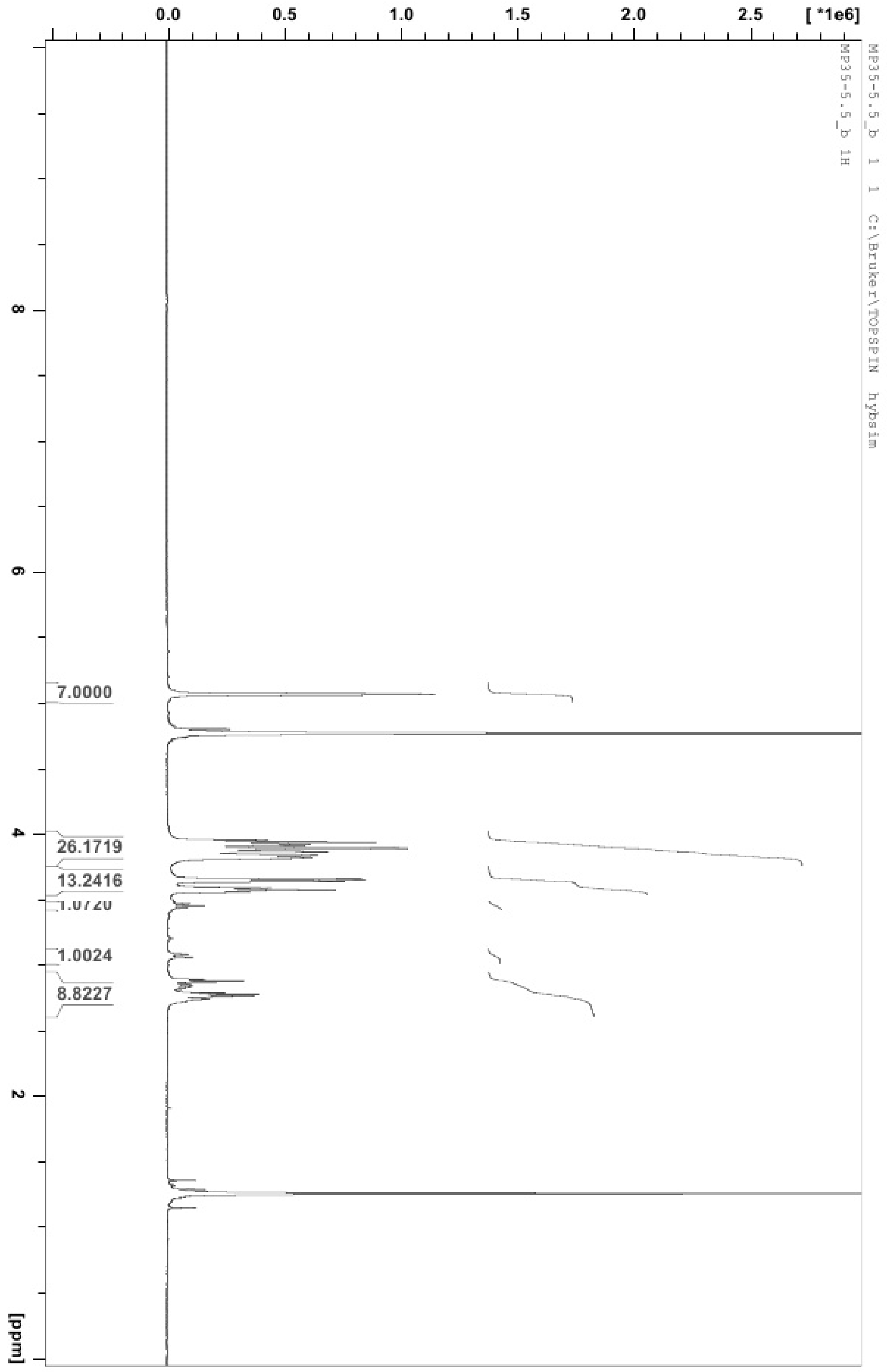

^{13}C NMR of compound **27** (150 MHz, D_2O)

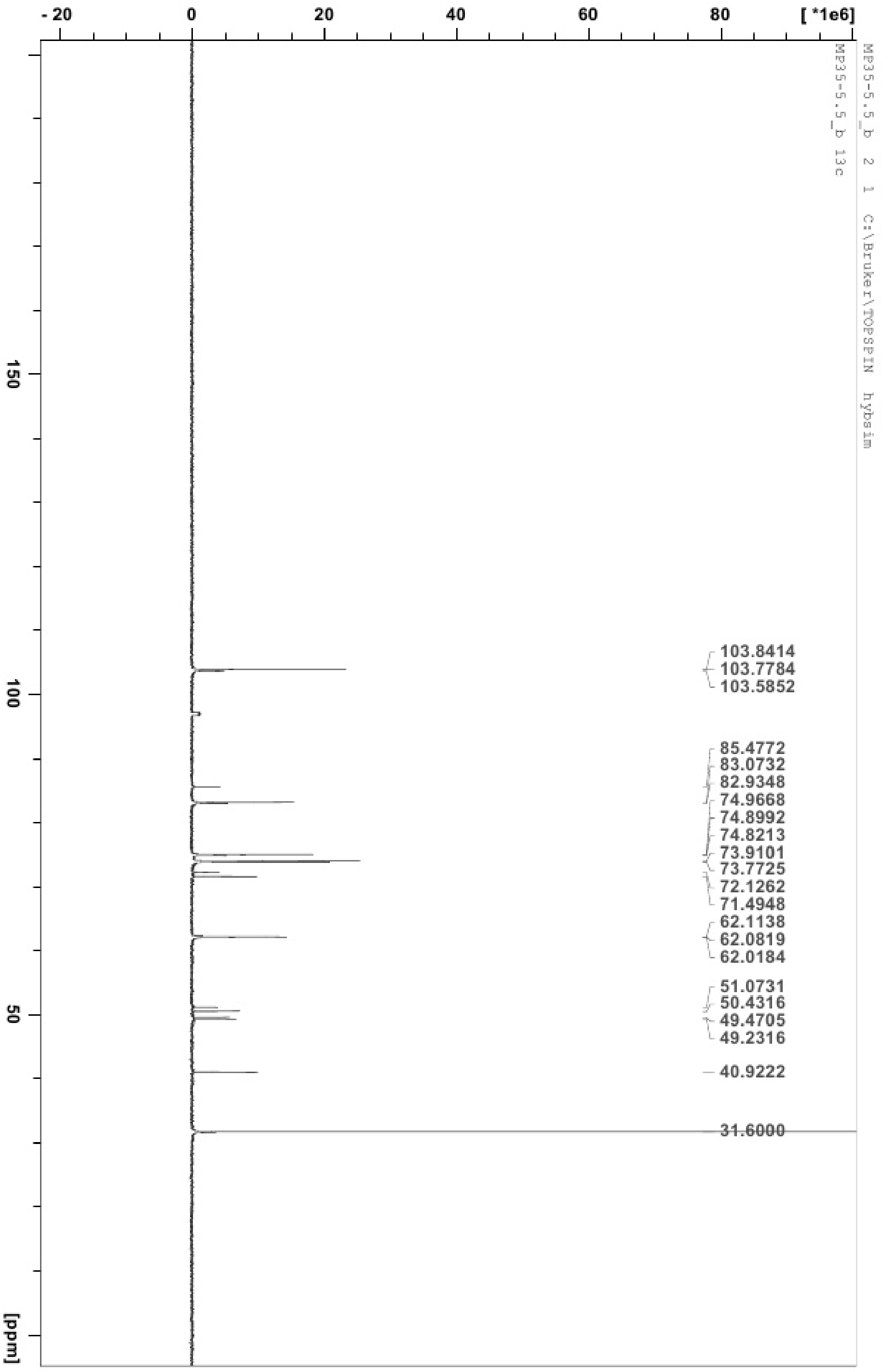

^1H NMR of compound **28** (600 MHz, D_2O)

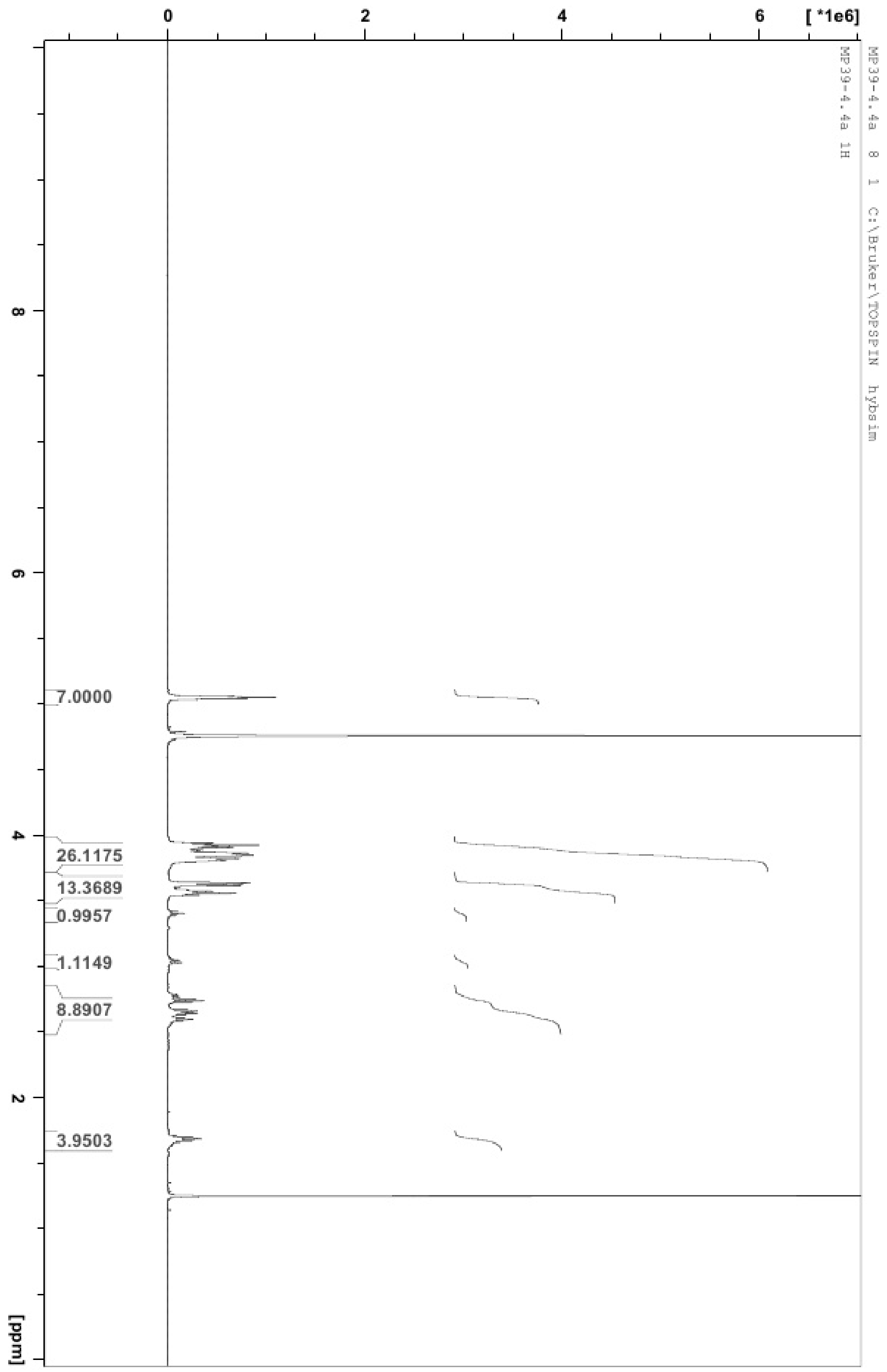

^{13}C NMR of compound **28** (150 MHz, D_2O)

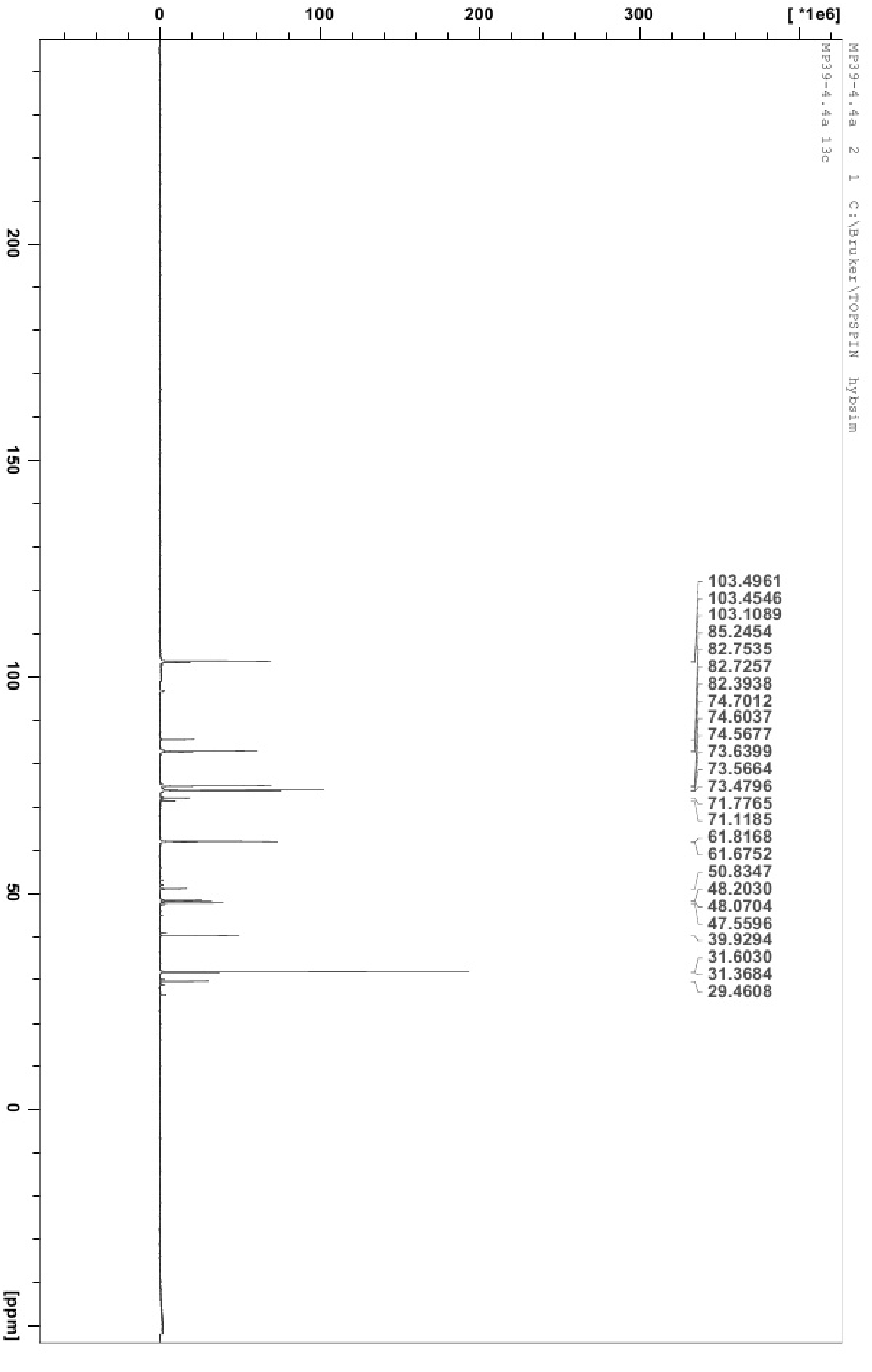

^{1}H NMR of compound **29** (600 MHz, D_2O)

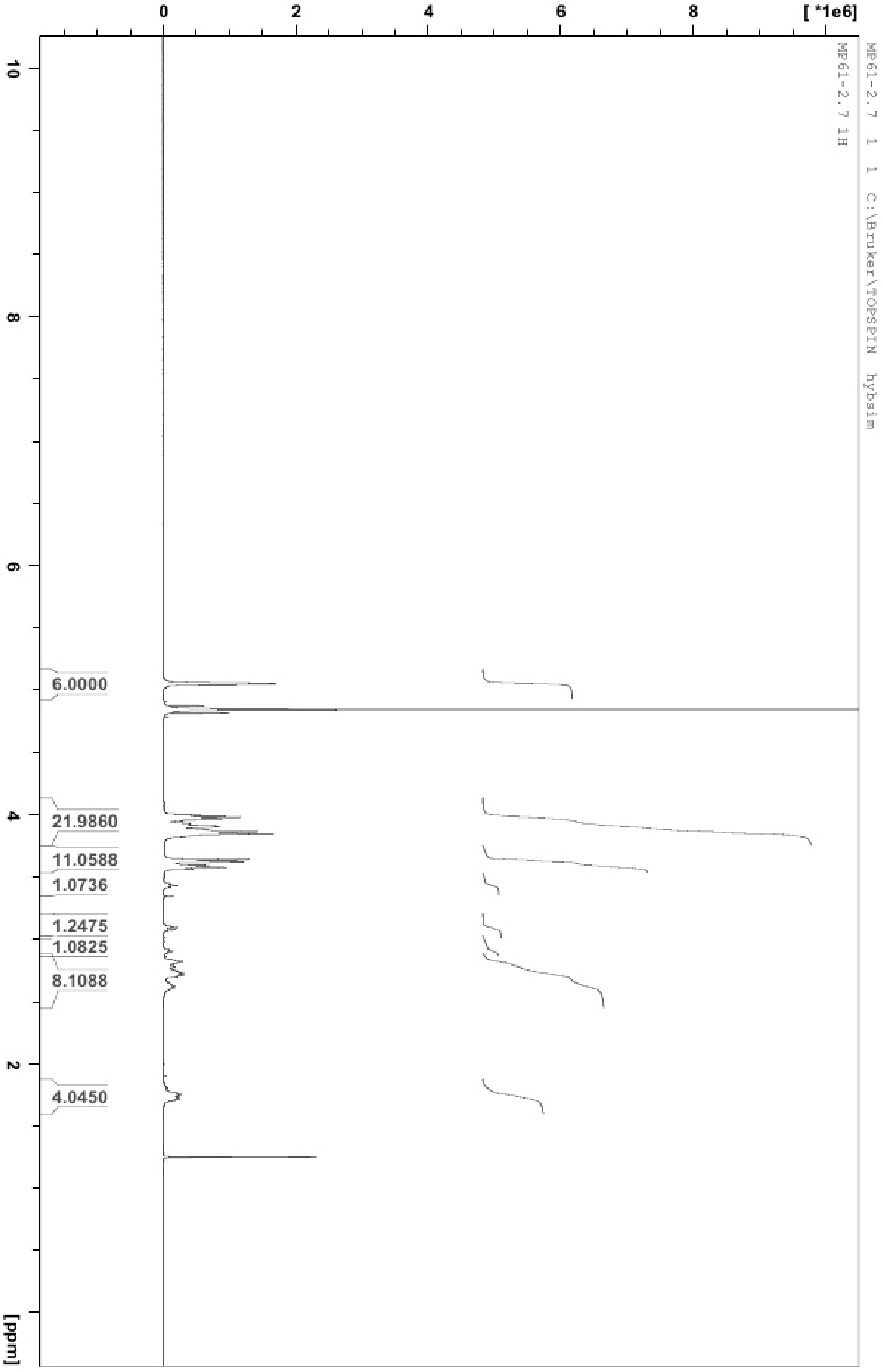

^{13}C NMR of compound **29** (150 MHz, D_2O)

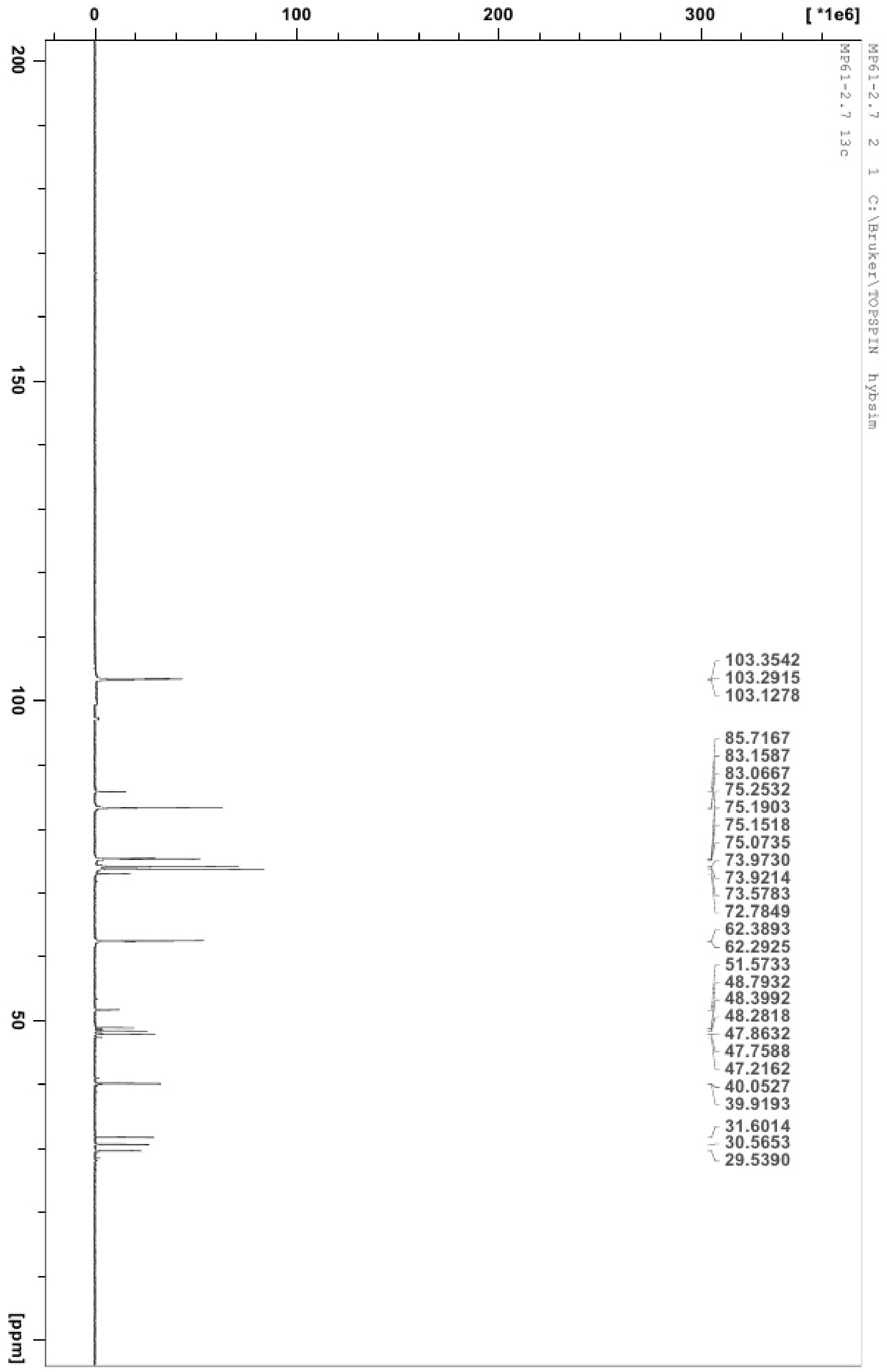

^{1}H NMR of compound **30** (600 MHz, D_2O)

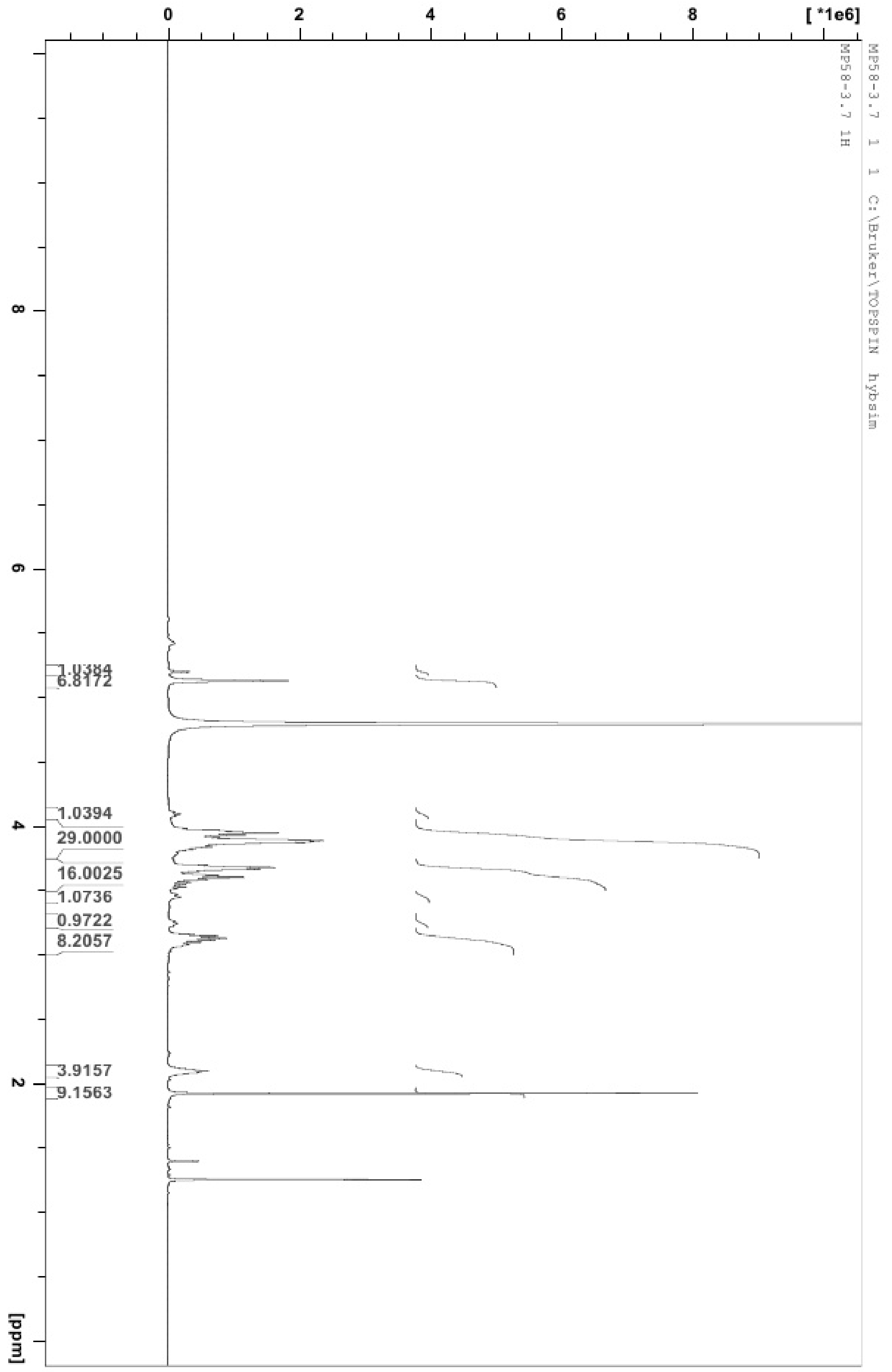

^{13}C NMR of compound **30** (150 MHz, D_2O)

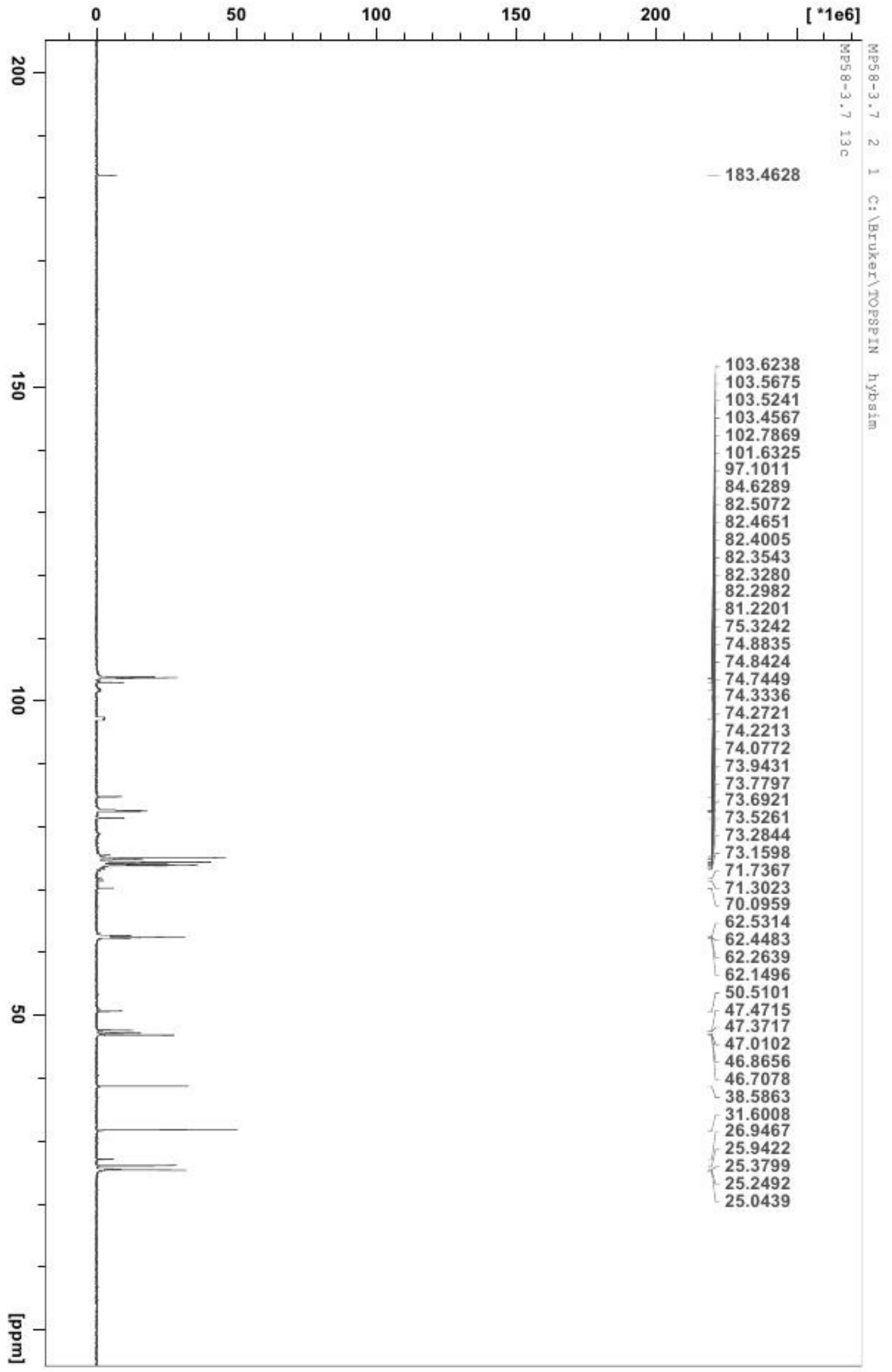

^{1}H NMR of compound **31** (600 MHz, D_2O)

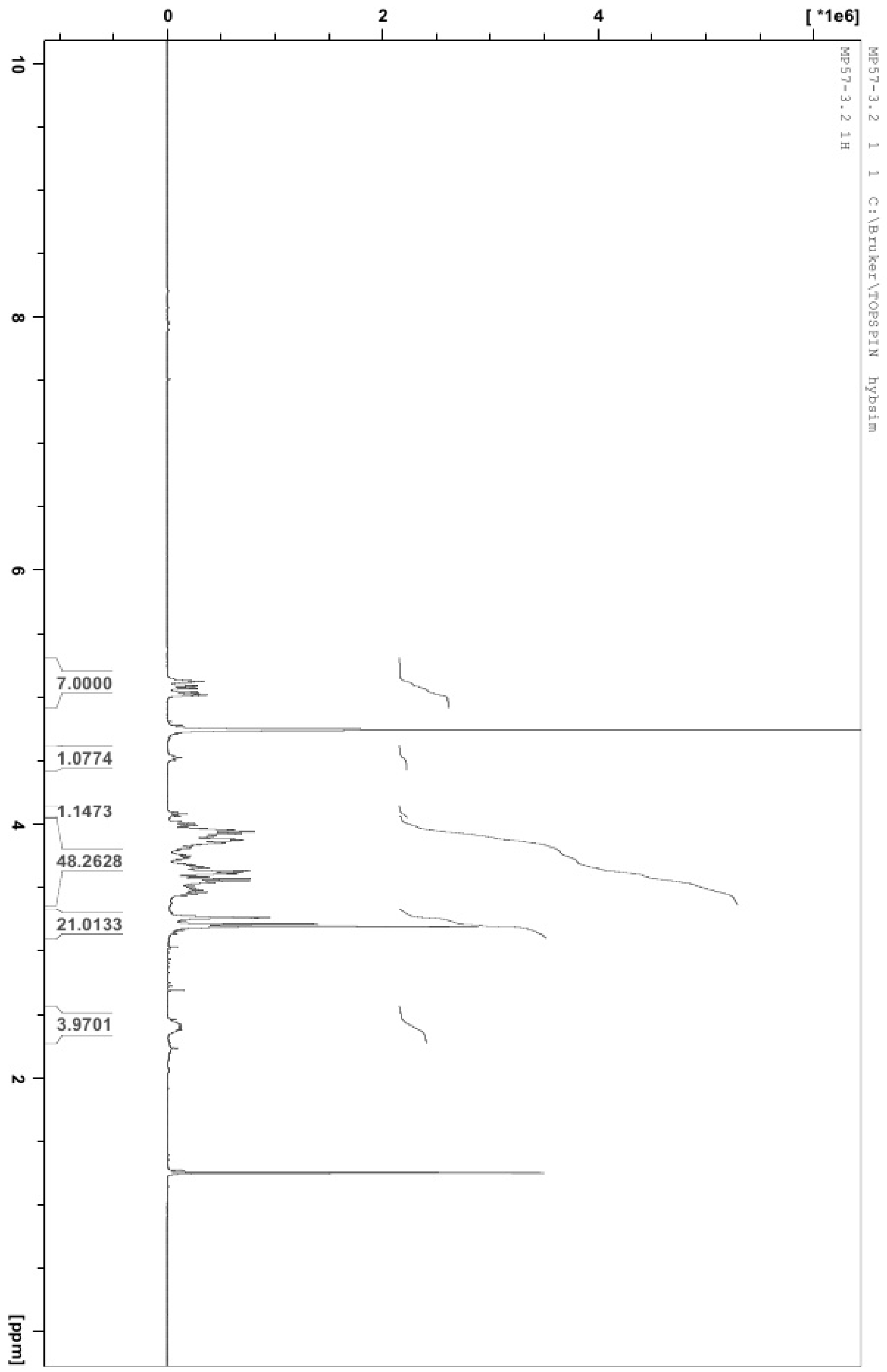

^{13}C NMR of compound **31** (150 MHz, D_2O)

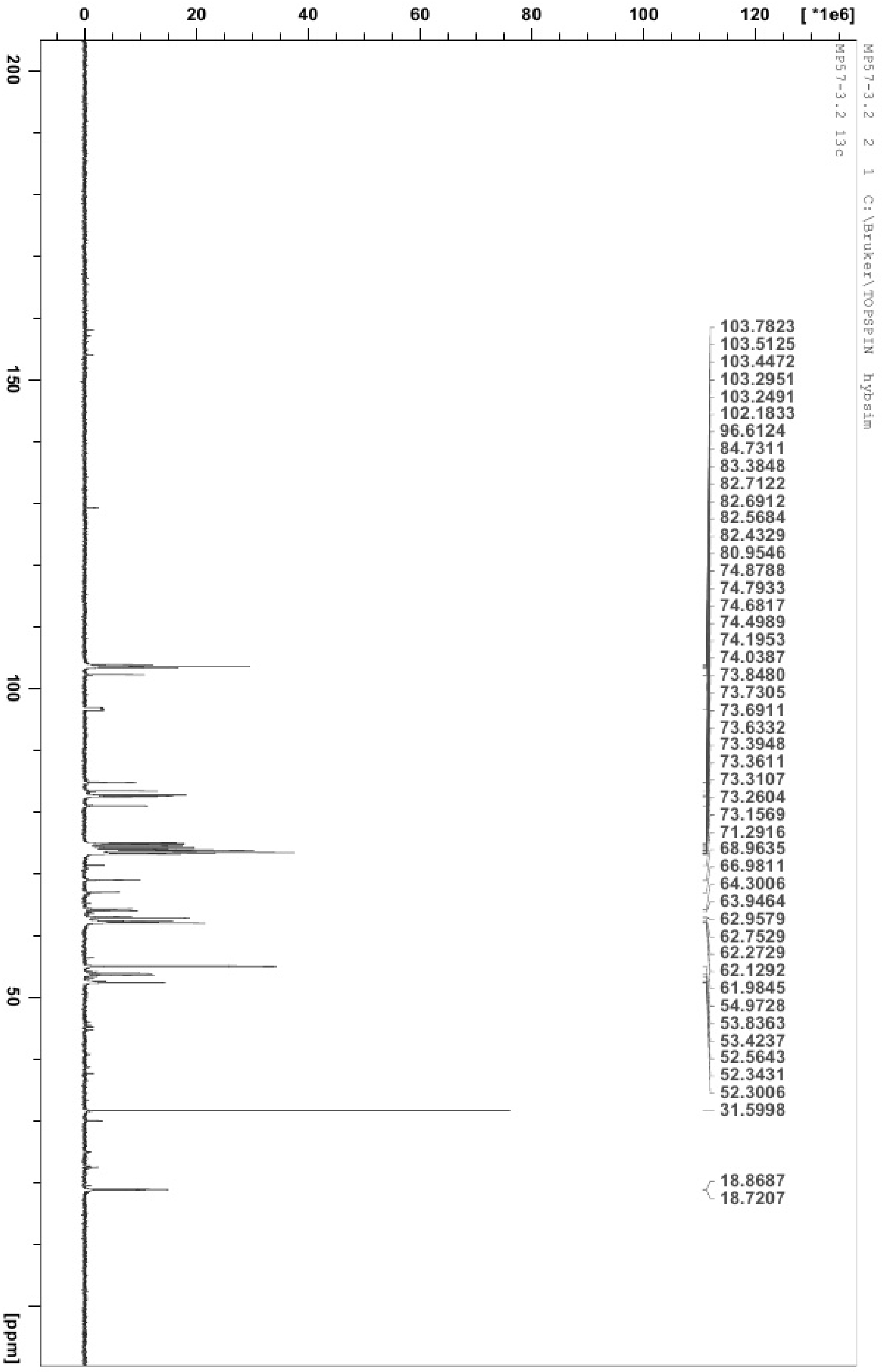

^{1}H NMR of compound **32** (600 MHz, D_2O)

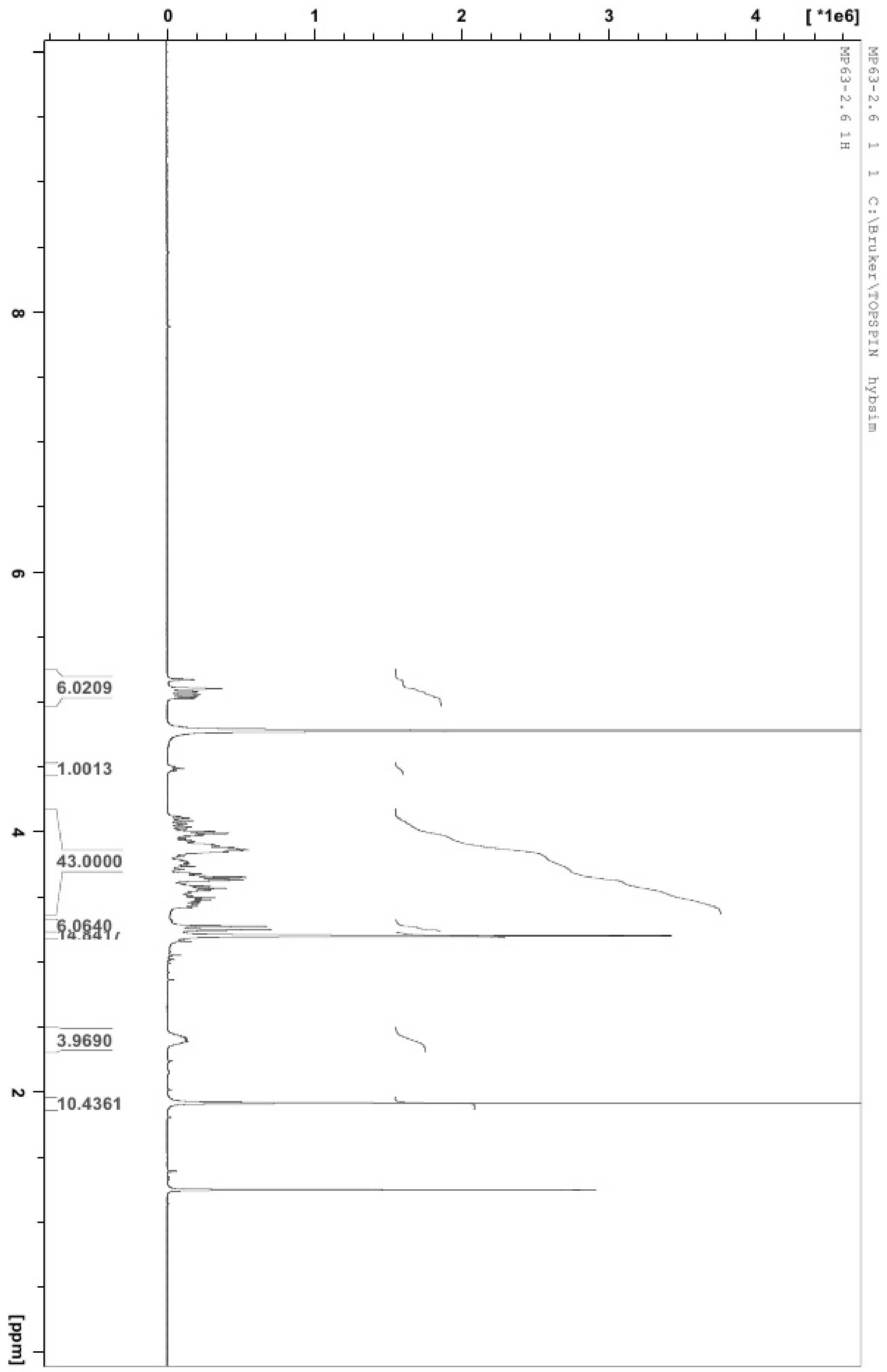

^{13}C NMR of compound **32** (150 MHz, D_2O)

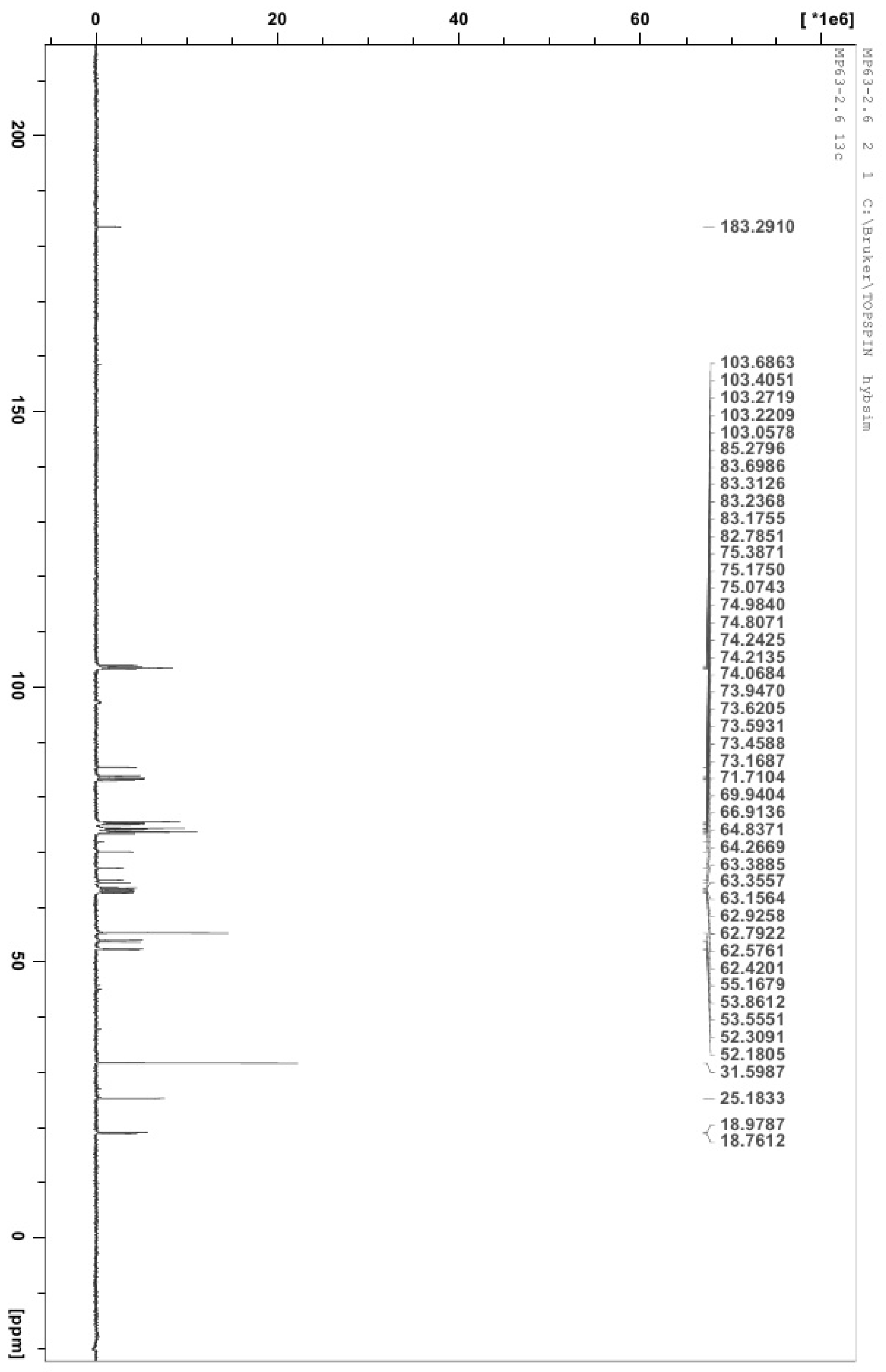

^{1}H NMR of compound **33** (600 MHz, D_2O)

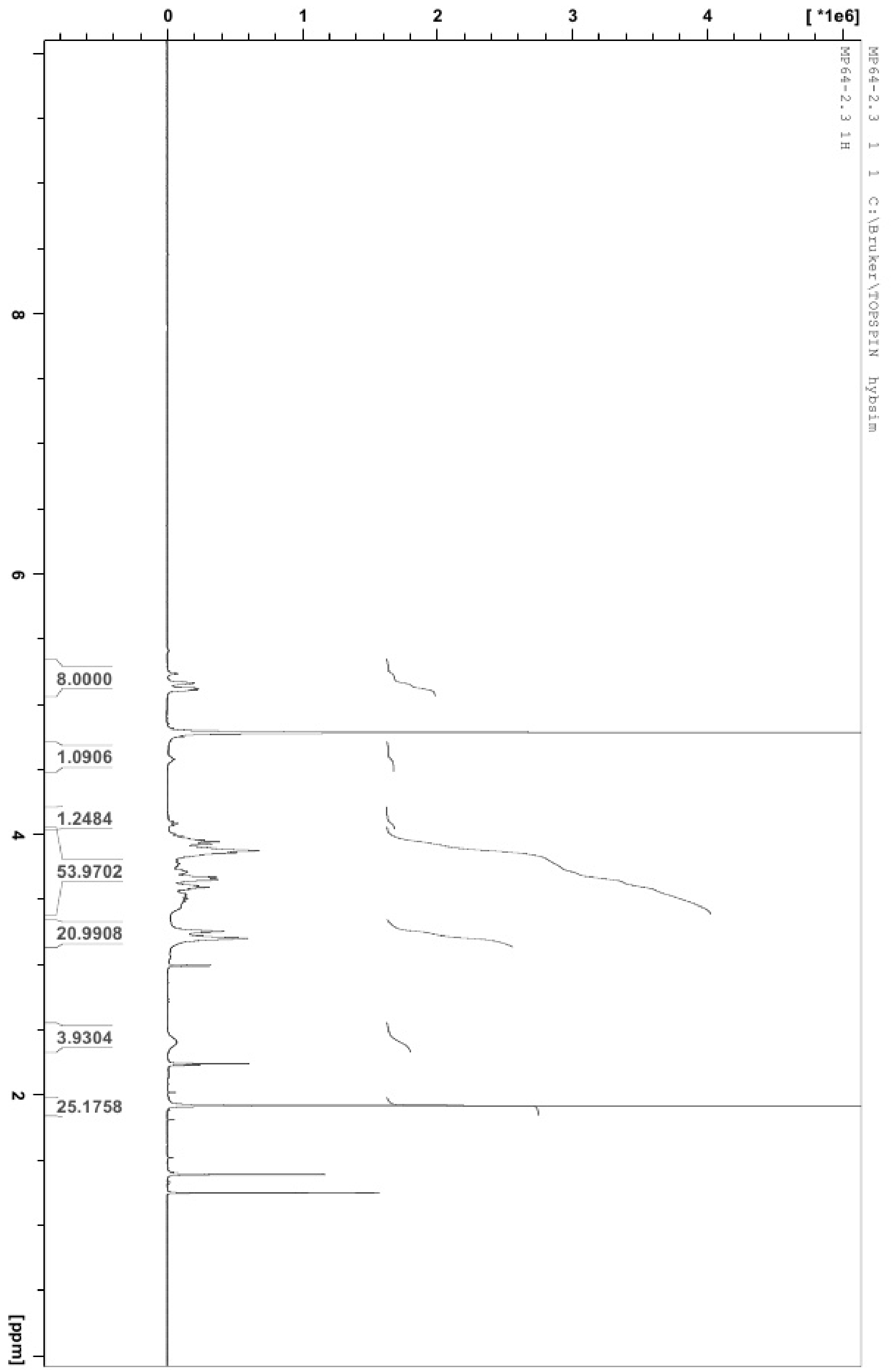

^{13}C NMR of compound **33** (150 MHz, D_2O)

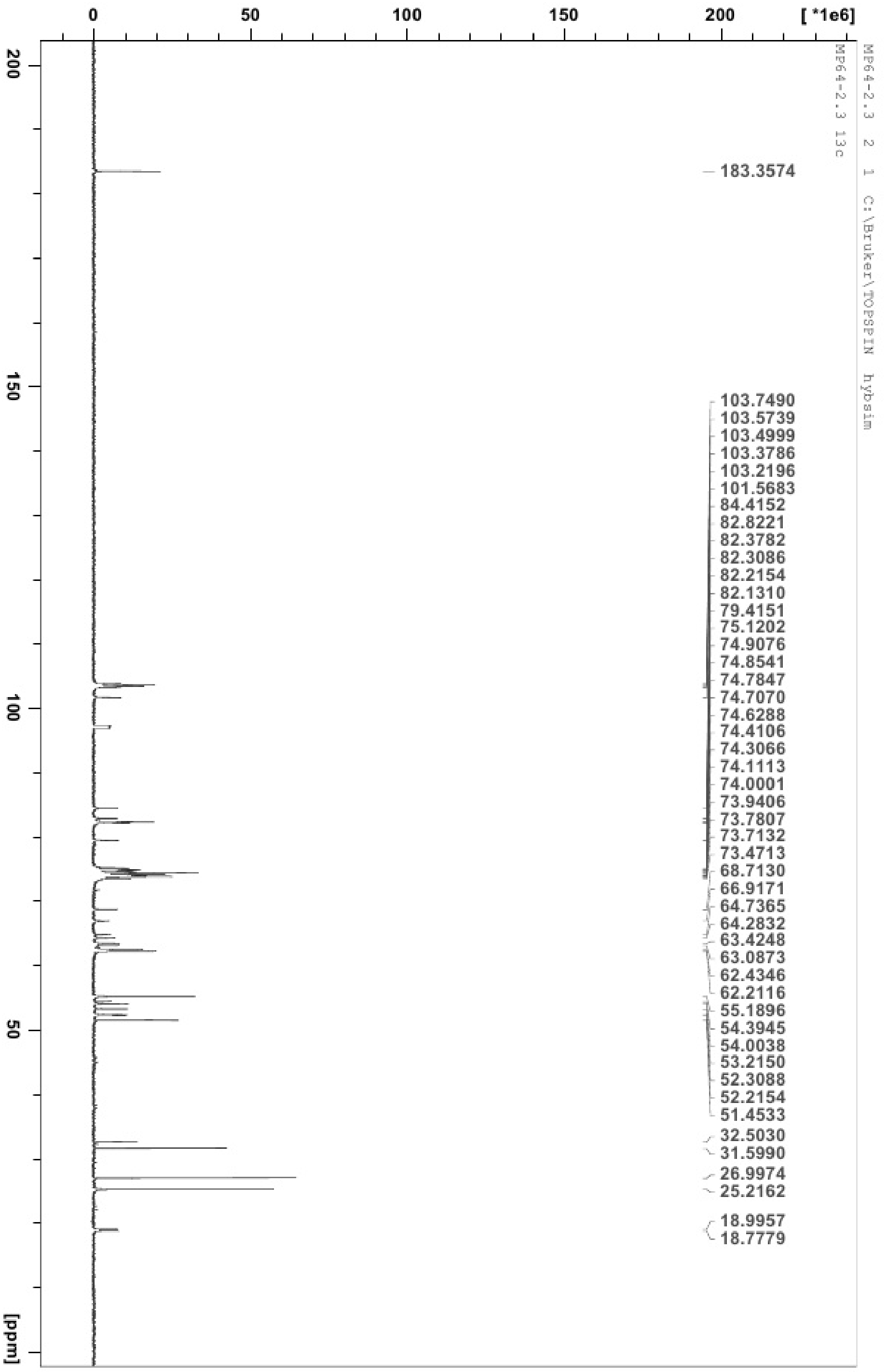

Characterization of compounds by 2D NMR

Structure (i.e. on which nitrogen atom of the substituent is the CD unit attached) of compound **26** (Figure S1) was confirmed by the combination of 2D NMR techniques HSQC and HMBC. Using HSQC CH_3 groups on N-1 can be distinguished. In HMBC (Figure S2) we can see cross-peaks between hydrogens of C**H**$_3$a and carbons **C**H$_3$b, C-1' and C-6^I. Also cross-peaks between hydrogens of C**H**$_3$b and carbons **C**H$_3$a, C-1' and C-6^I can be observed. Mutual interaction of CH_3a and CH_3b confirms their attachment to the same nitrogen atom. Their correlation with cyclodextrin C-6^I confirms the binding of the substituent to the nitrogen bearing two CH_3 groups. Also both, CH_3a and CH_3b, have cross-peak with C-1' carbon of the substituent. In HMBC we can see mutual correlation of CH_3 groups attached to N-2 and also with carbons C-3'a C-4'. All these facts confirm the proposed structure **26**. Very similar considerations were applied for characterization of compounds **25, 31**, **32** or **33** (2D NMR spectra for **32** attached).

Note: The signal marked as impurity in the Figure S2 belongs to the tetramethylammonium cation which is present in the final product in the amount below 2%. It is a side product most probably created by methylation of dimethylamine which is formed during the reaction by the partial decomposition of the DMF (which is used as a solvent). The content of this impurity increases when the reaction is performed at higher temperature.

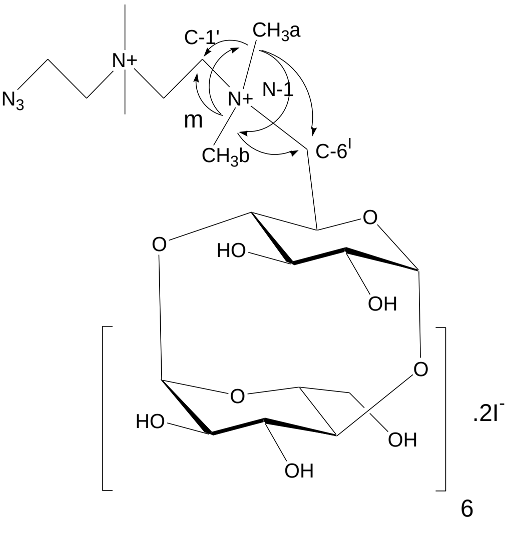

m = 2 **26**

Figure S1: Structure of compound 6 with numbering of the discussed atoms. Interactions observed in the 2D spectra are indicated by arrows.

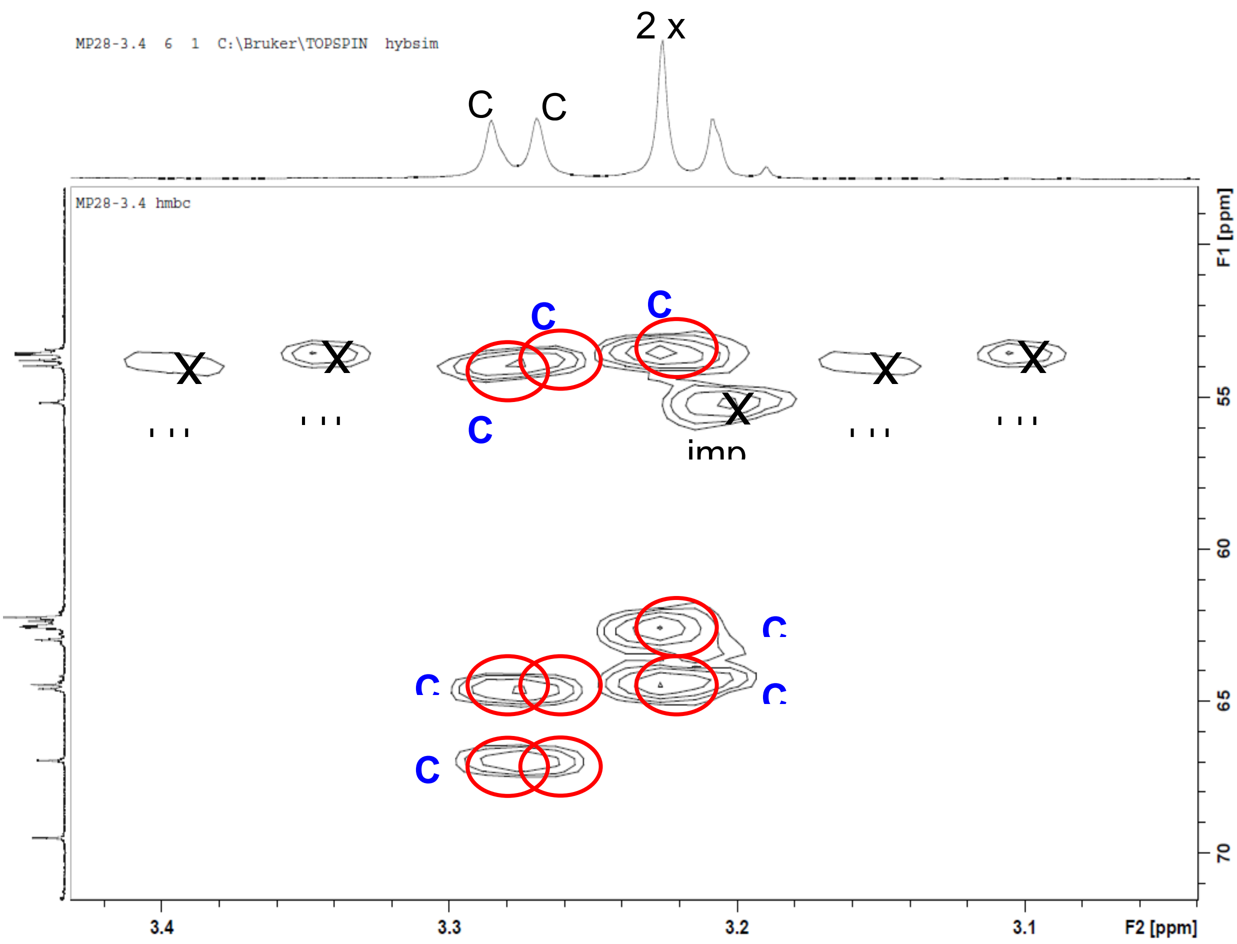

Figure S2: HMBC detail important for the determination of the structure of compound 26.

2D NMR Spectra

Compound **26** HMBC

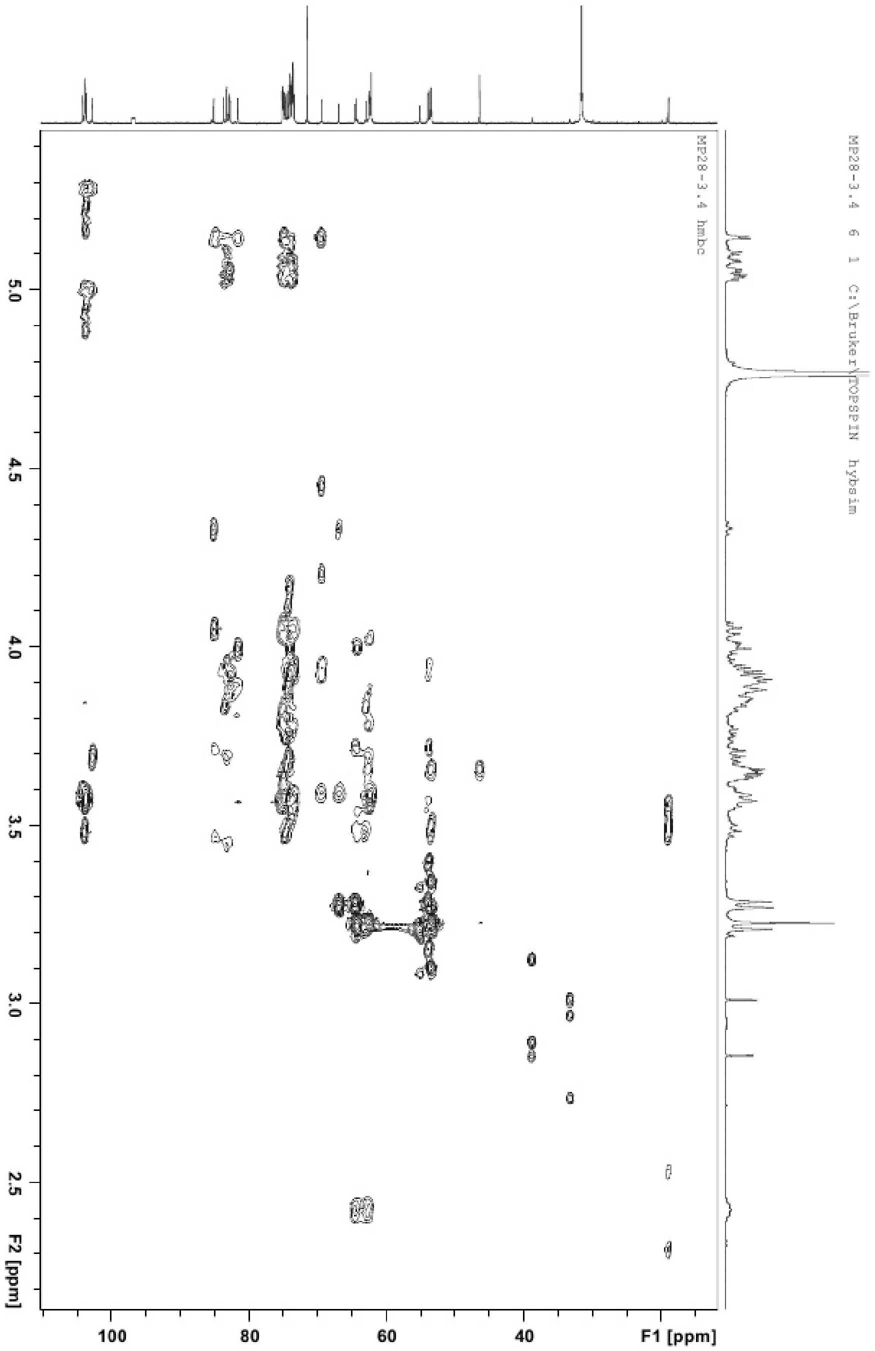

Compound **26** HMBC – detail

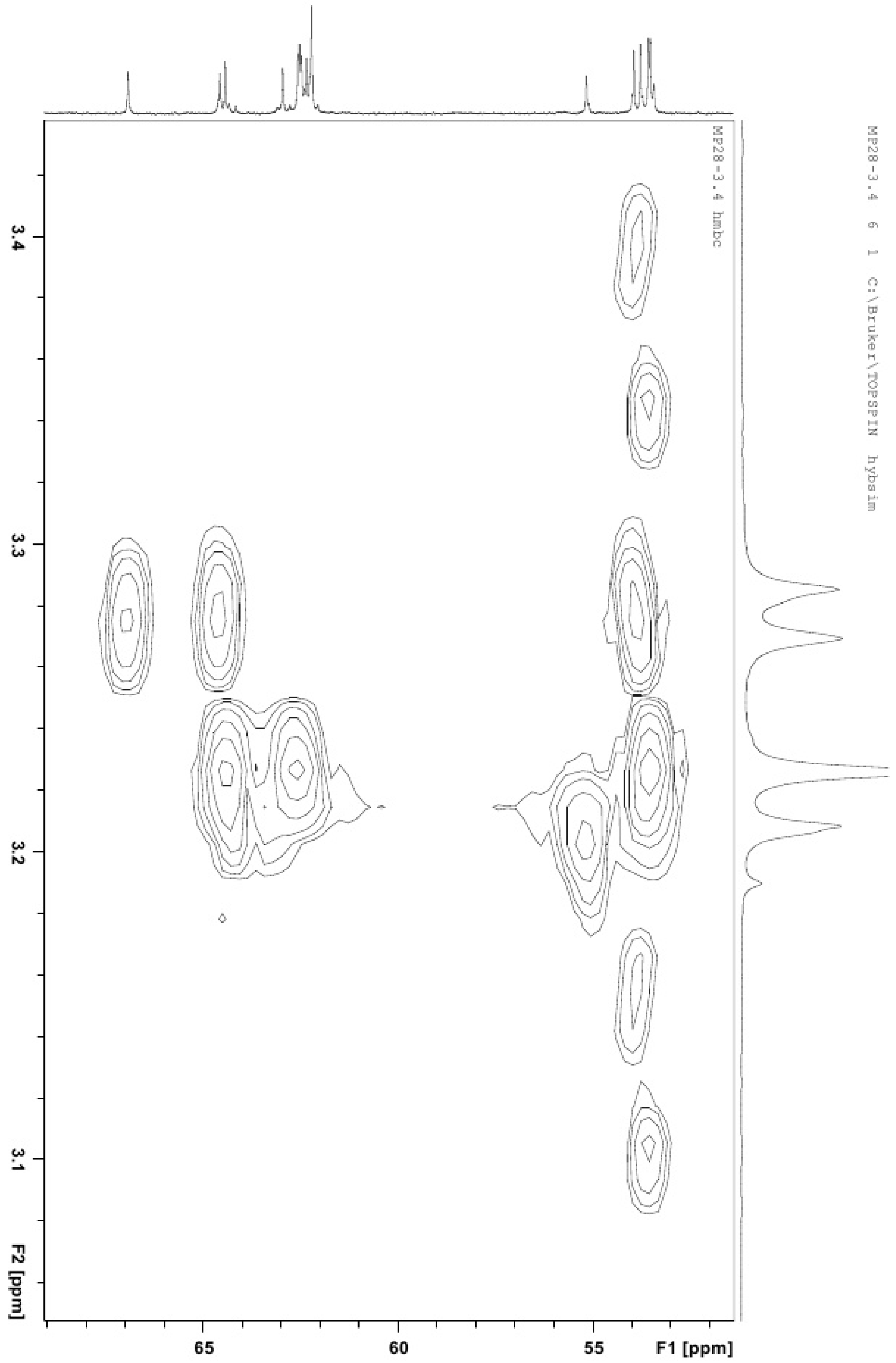

Compound **26** HMBC – detail 2

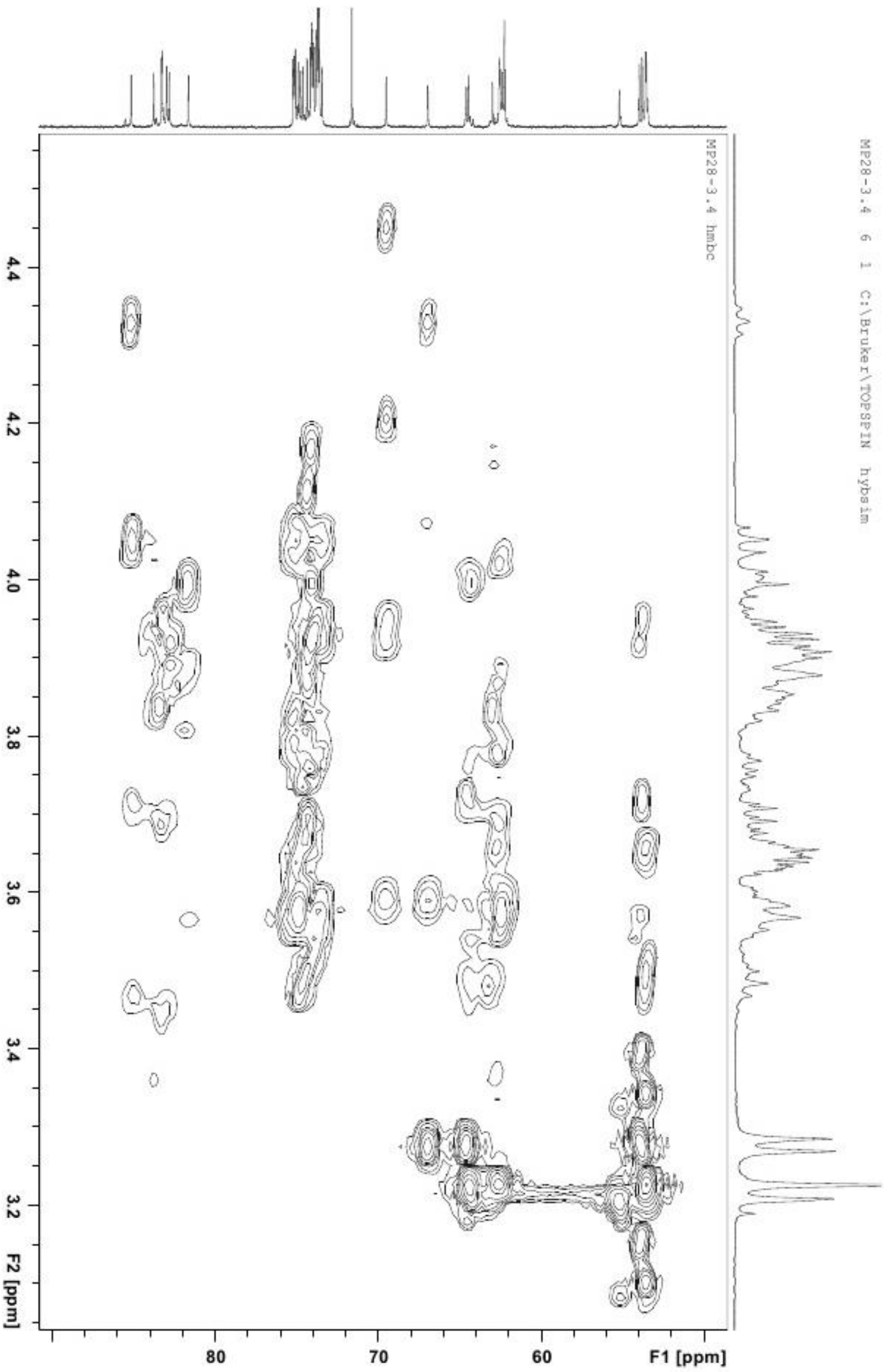

Compound **26** HSQC

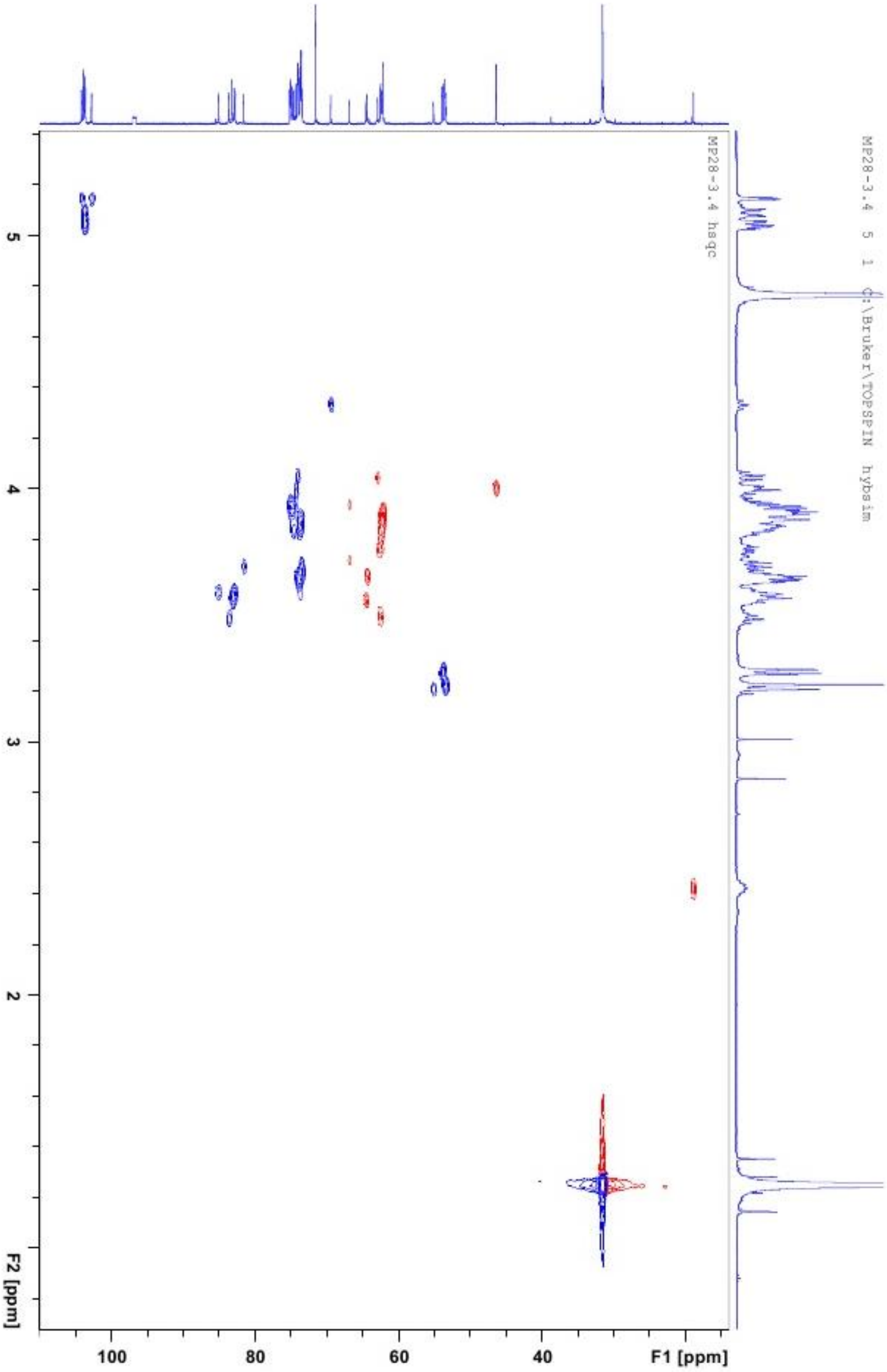

Compound **26** HSQC – detail

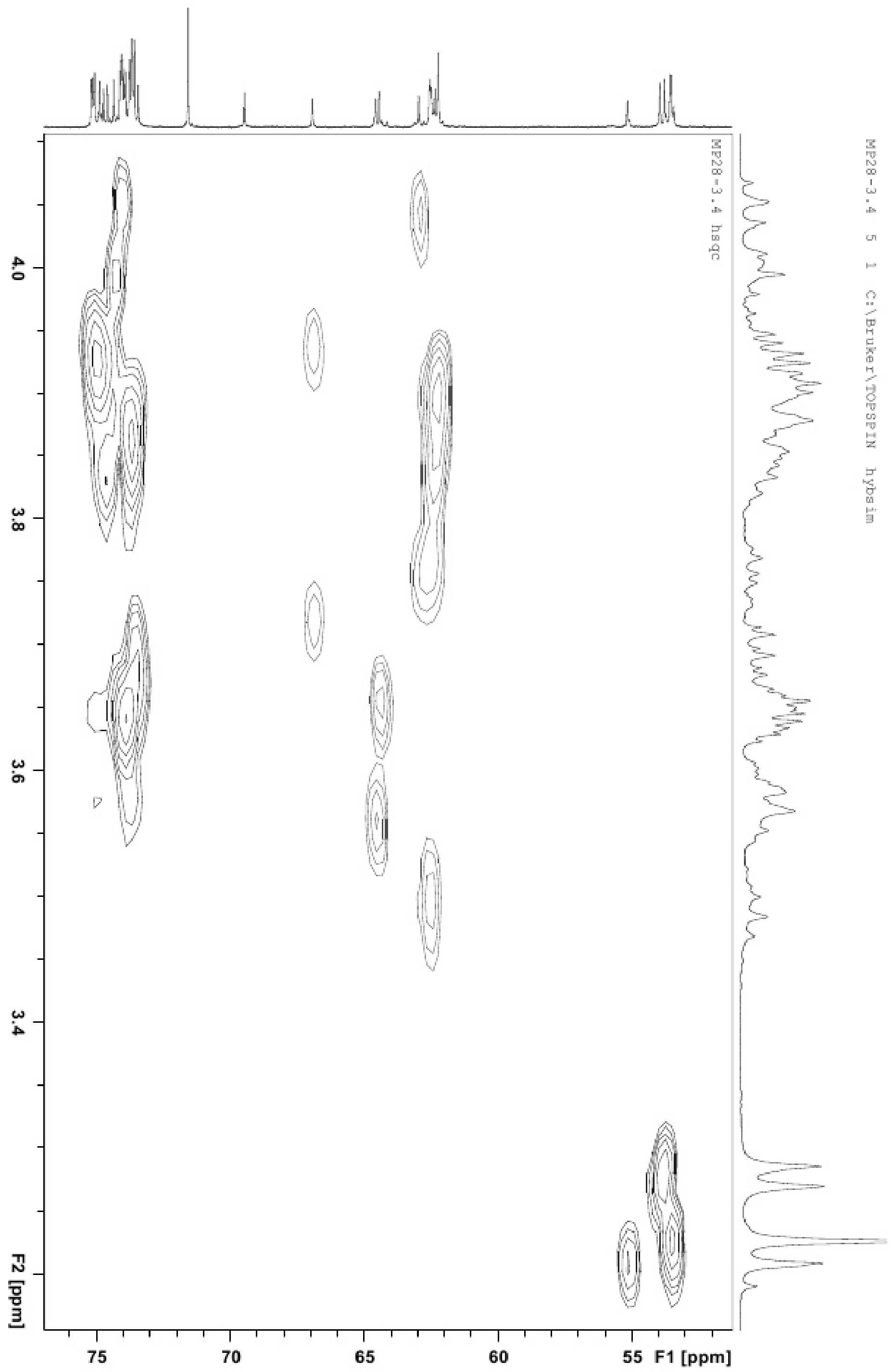

Compound **26** HSQC – detail 2

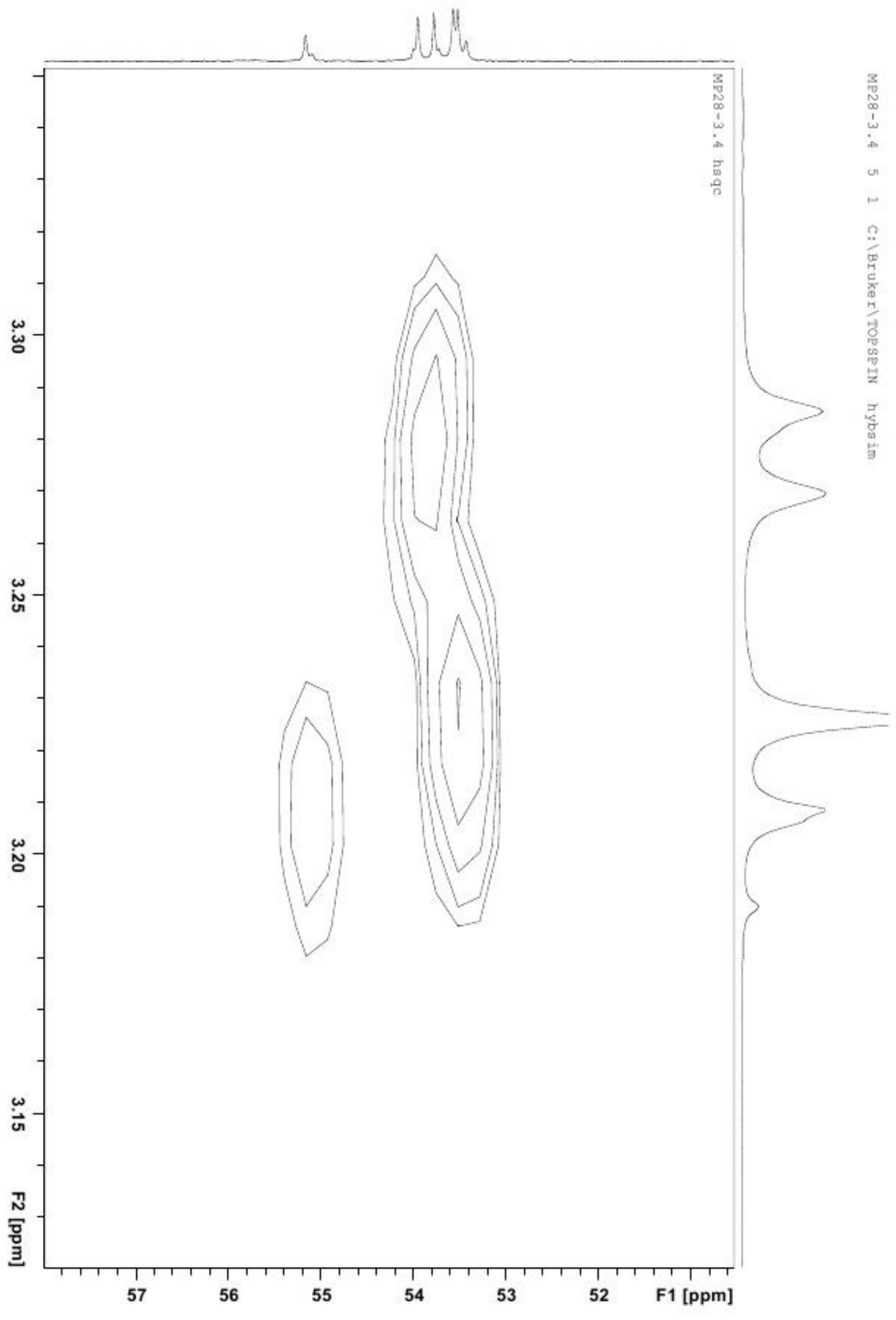

Compound **32** HMBC

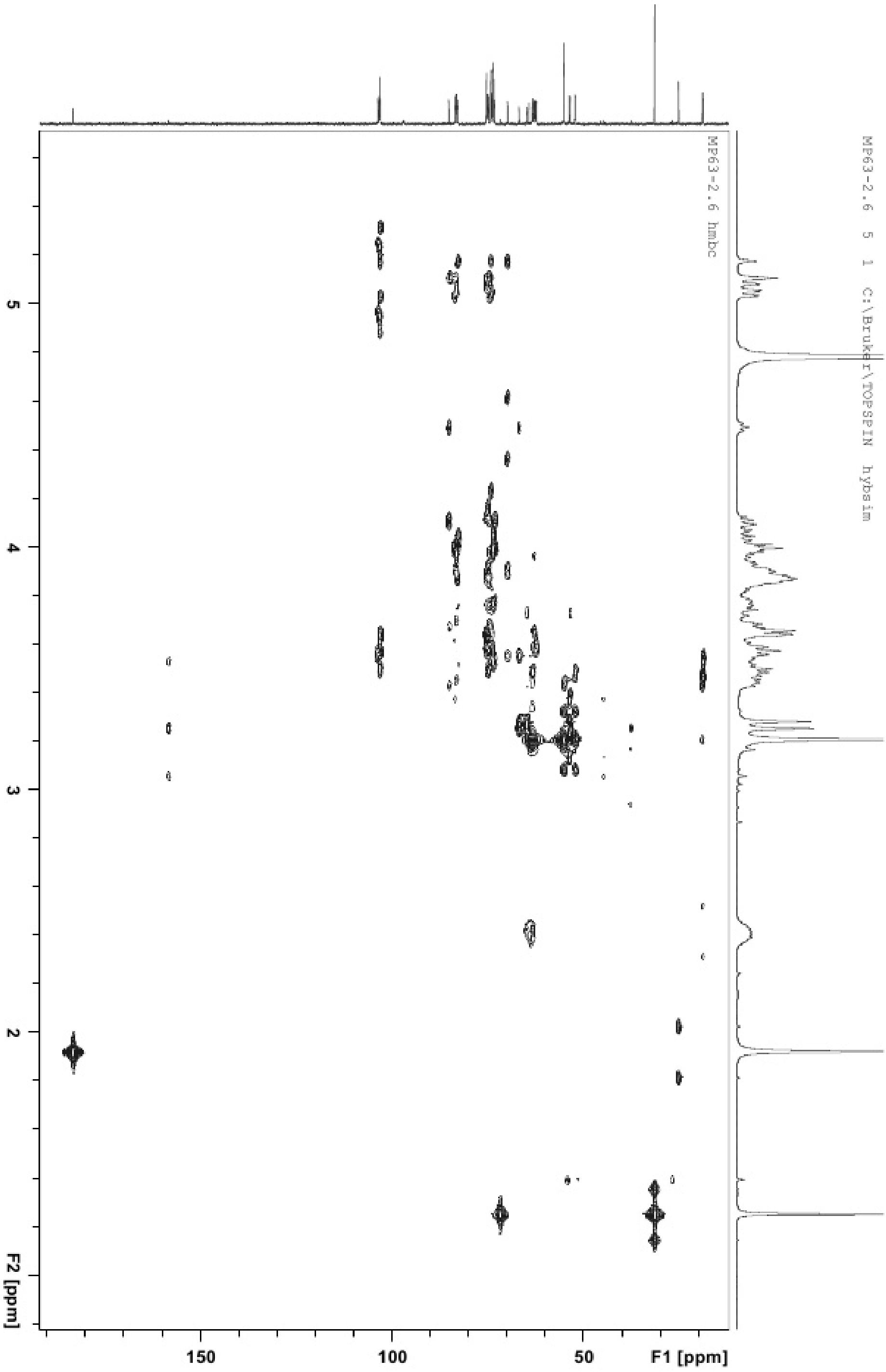

Compound **32** HSQC

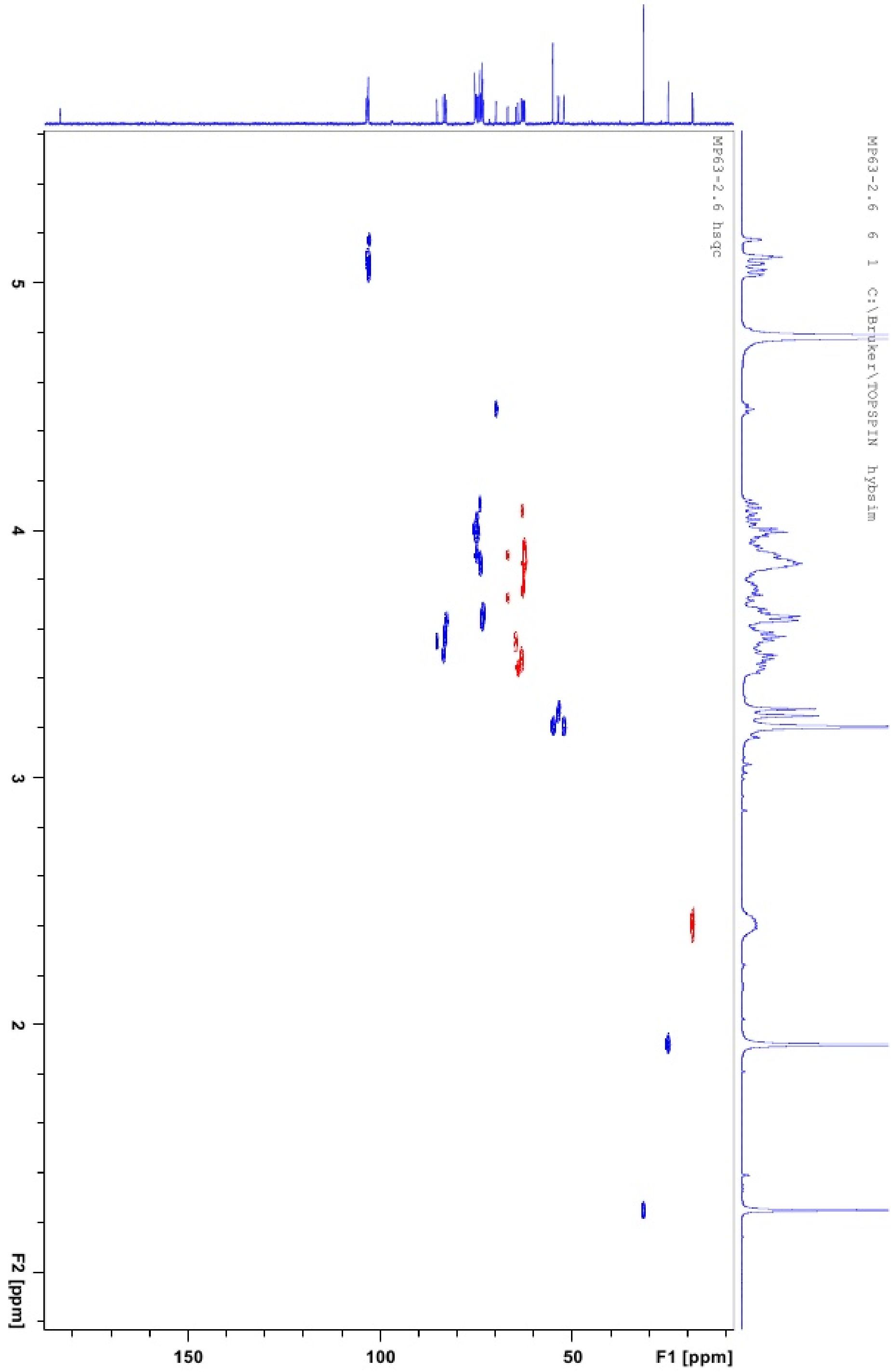

Supplement: File 1 — File Name: S1.pdf. Experimental part and copies of 1H and 13C NMR spectra of prepared compounds. [file Beilstein_J_Org_Chem-10-1390-s001.pdf]
